# Transcriptomic analysis of developmental features of *Bombyx mori* wing disc during metamorphosis

**DOI:** 10.1186/1471-2164-15-820

**Published:** 2014-09-27

**Authors:** Jun Ou, Hui-Min Deng, Si-Chun Zheng, Li-Hua Huang, Qi-Li Feng, Lin Liu

**Affiliations:** Laboratory of Molecular and Developmental Entomology, Guangdong Provincial Key Lab of Biotechnology for Plant Development, School of Life Sciences, South China Normal University, Guangzhou, 510631 China

**Keywords:** *B. mori*, Wing disc, Metamorphosis, Transcriptome, RNA-Seq

## Abstract

**Background:**

Wing discs of *B. mori* are transformed to pupal wings during the larva-to-pupa metamorphosis with dramatic morphological and structural changes. To understand these changes at a transcriptional level, RNA-seq of the wing discs from 6-day-old fifth instar larvae (L5D6), prepupae (PP) and pupae (P0) was performed.

**Results:**

In total, 12,254 transcripts were obtained from the wing disc, out of which 5,287 were identified to be differentially expressed from L5D6 to PP and from PP to P0. The results of comprehensive analysis of RNA-seq data showed that during larvae-to-pupae metamorphosis, many genes of 20E signaling pathway were up-regulated and those of JH signaling pathway were down-regulated. Seventeen transcription factors were significantly up-regulated. Cuticle protein genes (especially wing cuticle protein genes), were most abundant and significantly up-regulated at P0 stage. Genes responsible for the degradation and *de novo* synthesis of chitin were significantly up-regulated. There were A and B two types of chitin synthases in *B. mori*, whereas only chitin synthase A was up-regulated. Both trehalose and D-fructose, which are precursors of chitin synthesis, were detected in the hemolymph of L5D6, PP and P0, suggesting *de novo* synthesis of chitin. However, most of the genes that are related to early wing disc differentiation were down-regulated.

**Conclusions:**

Extensive transcriptome and DGE profiling data of wing disc during metamorphosis of silkworm have been generated, which provided comprehensive gene expression information at the transcriptional level. These results implied that during the larva-to-pupa metamorphosis, pupal wing development and transition might be mainly controlled by 20E signaling in *B. mori*. The 17 up-regulated transcription factors might be involved in wing development. Chitin required for pupal wing development might be generated from both degradation of componential chitin and *de novo* synthesis. Chitin synthase A might be responsible for the chitin synthesis in the pupal wing, while both trehalose and D-fructose might contribute to the *de novo* synthesis of chitin during the formation of pupal wing.

**Electronic supplementary material:**

The online version of this article (doi:10.1186/1471-2164-15-820) contains supplementary material, which is available to authorized users.

## Background

The domestic silkworm, *B. mori*, is not only an important economic insect, but also a model insect of Lepidoptera. The genome of silkworm has been reported [1, 2], and the genomic resources are available in Silkworm Genome Database: http://silkworm.genomics.org.cn/ and KAIKObase: http://sgp.dna.affrc.go.jp/KAIKObase/ [3, 4].

Wing disc is an imaginal disc of insect wing during larval stage. It consists of undifferentiated and proliferating cells [5]. In *B. mori,* as larvae develop to pupae, the wing discs undergo dramatic morphological changes and evaginate from the body to form the pupal wing. These changes and processes are regulated by insect hormones and hundreds of genes [6].

20-hydroxyecdysone (20E) and juvenile hormone (JH) are two major hormones in insects and regulate different biological processes, including growth, molting, and reproduction [7, 8]. Both 20E and JH are present at larval stages and they are antagonistic in many aspects. For example, 20E induces periodic ecdysis while JH maintains the "status quo" status of insects at larval stage [9, 10]. Insect metamorphosis is initiated by a surge of 20E at the end of last larval stage when JH titer is low [11]. In the 20E signaling pathway, ecdysone receptor (EcR) binds with ultraspiracle (USP) to form a heterodimer, which binds to the response element of early response genes, such as *Broad complex* (*BR-C*), *E74* and *E75* [12], to initiate the expression. Early-late response genes, including *HR3*, *HR39* and *E78*, are also involved in the 20E signal transduction pathway [13]. In the JH signal transduction pathway, methoprene-tolerant (Met) was regarded as a potential candidate receptor for JH [14–17]. Knock-out *Met* led to a down-regulation of JH-response gene *Krüppel homolog 1* (*Kr-h1*), and the insect showed a precocious phenotype in *Tribolium castaneum* [17]. In *B. mori*, Met has two isoforms (Met1 and Met2). Met2 combines with steroid receptor coactivator (SRC, also known as "FISC" or "Taiman") to form a heterodimer and induces the expression of *Kr-h1* [18]. In *B. mori*, 20E and JH signaling pathways have been found to be involved in normal wing development [19–21], but no detailed analyses of the two signaling pathways was performed at the transcriptional level.

Genes related to wing disc development at the embryonic stage and early stages were well elucidated in *Drosophila*. Transcription factors, such as *Engrailed* (*En*), *Apterous* (*Ap*), *Scalloped* (*Sd*) and *Vestigial* (*Vg*), secreted proteins, such as *Hedgehog* (*Hh*), *Decapentaplegic* (*Dpp*), *Wingless* (*Wg*) and *Fringe* (*Fng*), receptors such as *Epidermal growth factor receptor* (*Egfr*) and *Notch* (*N*), and their ligands such as *Serrate* (*Ser*) and *Delta* (*Dl*) were found to be involved in the early differentiation and embryonic development of *Drosophila* wing discs [5, 22–27]. However their roles in wing transition development from wing disc to pupal wing were not reported.

Cuticle protein and chitin are main structural components of cuticle in insects [28]. Thirteen wing cuticle protein genes (WCP) have been identified in *B. mori* wing disc, including *WCP1a*, *WCP1b*, *WCP2*, *WCP3*, *WCP4*, *WCP5*, *WCP6*, *WCP71a*, *WCP7b*, *WCP8*, *WCP9*, *WCP10* and *WCP11* [29–31]. Chitin degradation and synthesis in insects were well-studied [28]. Chitinase, β-N-acetylglucosaminidase and chitin deacetylase are three main enzymes that involved in chitin degradation, while four critical enzymes, trehalase, hexokinase, glucose-6-phosphate isomerase and glutamine and fructose-6-phosphate aminotransferase, are needed for the *de novo* synthesis of chitin (KEGG pathway map: ko00520). There are two kinds of chitin synthases in insects: chitin synthase 1 (class A) is responsible for the chitin synthesis in the epidermis and trachea, while chitin synthase 2 (class B) is critical for the chitin synthesis in the gut peritrophic membrane [28].

For the past decade, a variety of methods such as expressed sequence tags (ESTs) [32, 33], serial analysis of gene expression (SAGE) [34, 35], microarrays [36, 37], and RNA-seq technology [38] have been applied to gene expression analysis in the silkworm, but no information was available on detailed analysis of wing disc development during metamorphosis at the transcriptional level. In this study, RNA-seq method [39, 40] was used to get a comprehensive view on the gene expression of *B. mori* wing disc during metamorphosis.

## Results

### Transcriptome sequencing and statistics of gene expression

In total, 4,842,606, 4,741,356 and 4,852,047 clean reads were obtained from the wing disc at 6-day-old fifth instar larvae (L5D6), prepupae (PP) and pupae (P0) stages, respectively. These clean reads were mapped to reference sequences from Silkworm Genome Database using SOAPaligner/soap2 software [41] and their nucleotide sequences were assembled. A total of 12,254 assembled transcripts were obtained and the average length of the transcripts was 1,226 base pairs.

**Figure 1:**
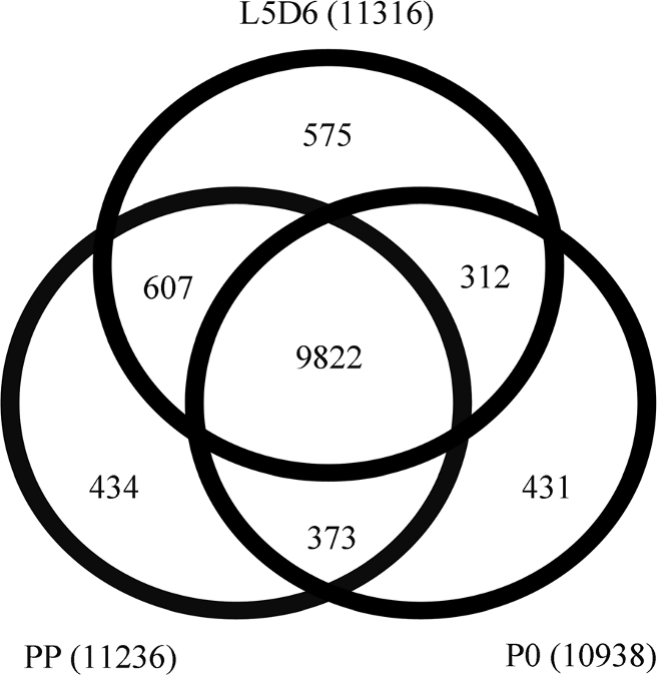
**Statistics of transcript numbers and distribution at L5D6, PP and P0.**

At L5D6, PP and P0 stages 11,316, 11,236, 10,938 assembled transcripts were obtained, respectively (Figure 1). Of the 12,254 assembled transcripts, 9,822 transcripts are commonly shared by the three stages; 607, 373, 312 transcripts are shared by two stages (L5D6 and PP, PP and P0, L5D6 and P0), respectively; 575, 434, 431 transcripts are specifically expressed at L5D6, PP and P0 stage, respectively (Figure 1). These results showed that most transcripts were commonly expressed in each stage, while the stage-specific transcripts might be important for the wing disc development at the specific stage.

### Differentially expressed transcripts and gene ontology (GO) classification

**Figure 2:**
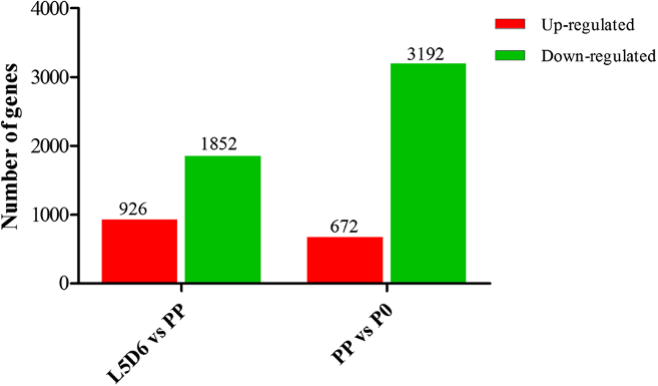
**Statistics of differentially expressed transcripts.**

In total, 5,287 differentially expressed transcripts were identified based on the standard process (see Methods). From L5D6 to PP (L5D6 vs PP), 2,778 transcripts were identified as differentially expressed, among which 926 were up-regulated and 1,852 were down-regulated (Figure 2). From PP to P0 (PP vs P0), 3,864 transcripts were identified as differentially expressed, of which 672 were up-regulated and 3,192 were down-regulated (Figure 2). It is surprising that there were more down-regulated transcripts than up-regulated transcripts during the transition from L5D6 to PP and from PP to P0 (Additional files 1 and 2), implying that with the transitional development of the wing disc during metamorphosis, expression of more and more genes was turned off.

**Figure 3:**
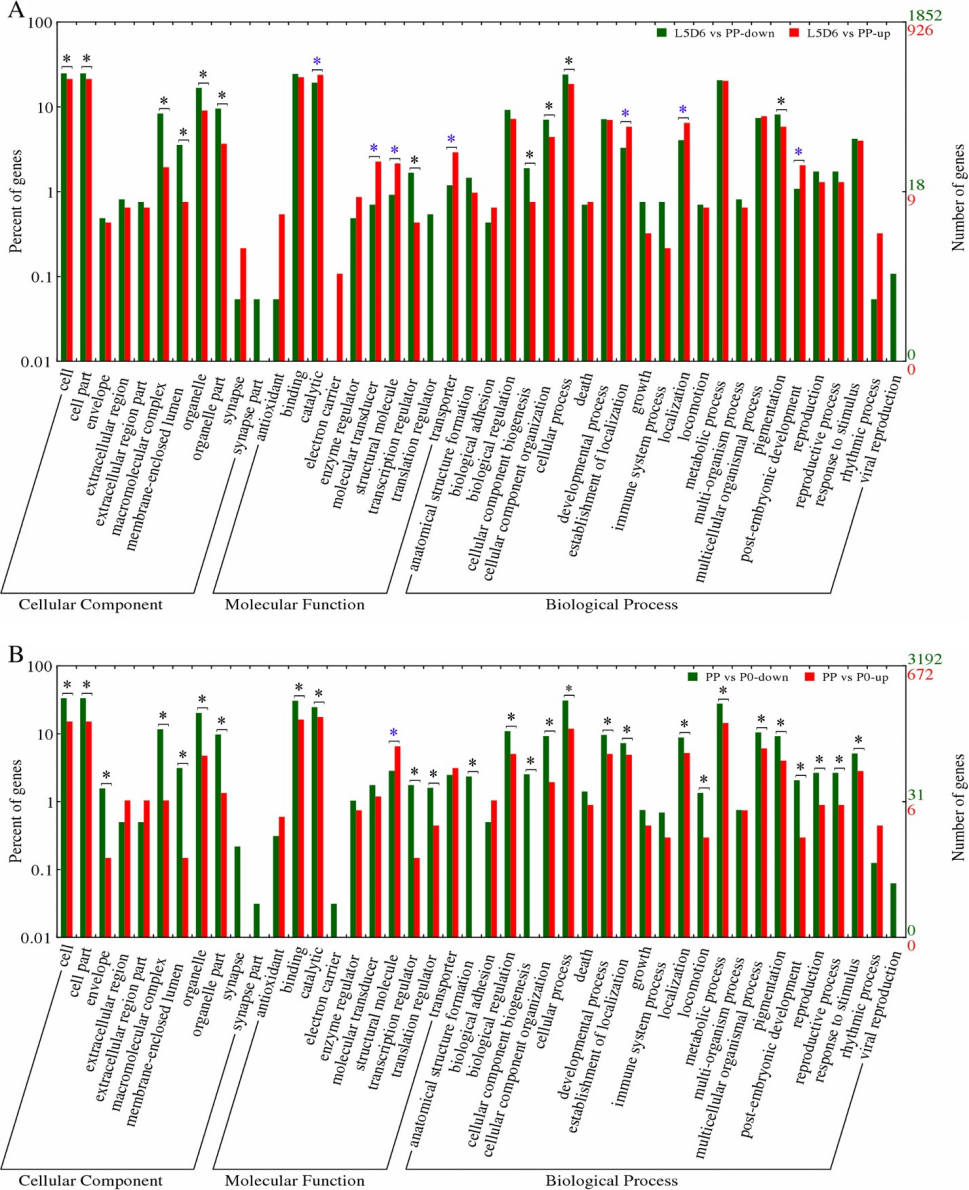
**GO classification of the differentially expressed transcripts.** The GO classification map was done by uploading the GO ID numbers of genes to BGI WEGO homepage mirror (GO archive: 2009-10-01). Asterisks indicate that the differences between the two stages are significant (p < 0.05). **(A)** GO classification of the differentially expressed transcripts in L5D6 vs PP. **(B)** GO classification of the differentially expressed transcripts in PP vs P0.

It is reasonable to hypothesize that developmental features of the wing disc depend on the activities of differentially expressed transcripts. Therefore, GO classification analysis of differentially expressed transcripts may explain the process of wing disc development. The GO ID numbers of differentially expressed transcripts were obtained from Blast2go software and the GO classification histogram was obtained by uploading GO ID number to BGI WEGO online plot software (see Methods). The results showed that binding and catalytic functions were the most abundant GO function items (>10%), both from L5D6 to PP and PP to P0 developmental stages (Figure 3A and 3B). Specifically, in the cellular component ontology, no dominant GO items of the up-regulated transcripts were found for L5D6 vs PP, while several dominant GO items of the down-regulated transcripts were found, including cell, cell part, macromolecular complex, membrane-enclosed lumen, organelle and organelle part (Figure 3A). The dominant GO items of the significantly up-regulated transcripts over down-regulated transcripts for L5D6 vs PP are catalytic, molecular transducer, structural molecular and transporter in molecular function ontology (Figure 3A). The significantly dominant GO item of the down-regulated transcripts over the up-regulated transcripts for L5D6 vs PP in molecular function ontology was transcription regulator, while the down-regulated transcripts of other functional GO items were not significantly more than the up-regulated transcripts in the same catalogs, including translation regulator, in which no up-regulated transcripts were detected for L5D6 vs PP (Figure 3A). For biological process ontology for L5D6 vs PP, the dominant GO items of the up-regulated transcripts included establishment of localization, localization and post-embryonic development, while the dominant GO items of the down-regulated transcripts included cellular component biogenesis, cellular component organization, cellular process and pigmentation (Figure 3A). Although some GO items in the three ontologies, such as synapse, synapse part, antioxidant, electron carrier and translation regulator, showed obviously distinctive differences in the numbers of transcript expression, they were not statistically significant (p < 0.05) by Pearson test [42] (Figure 3A).

On the other hand, for PP vs P0, the dominant GO items of significantly down-regulated transcripts in cellular component ontology included most of catalogues except extracellular (part) and synapse (part), while no dominant GO items of significantly up-regulated transcripts were found (Figure 3B). In molecular function ontology, the only dominant GO item of the significantly up-regulated transcripts was structural molecule; the dominant GO items of the significantly down-regulated transcripts included binding, catalytic, transcription regulator and translation regulator, while the other GO items were not significantly different between up- and down-regulated transcripts (Figure 3B). For cellular process, no dominant GO items of the significantly up-regulated transcripts were detected, while most catalogues were significantly dominant for the down-regulated transcripts over the up-regulated transcripts (Figure 3B).

### The 20E signal transduction pathway was more active than the JH signal transduction pathway during metamorphosis

To investigate activity of the 20E and JH signal transduction pathways in the wing disc during metamorphosis, the expression patterns of the genes that are involved in the 20E and JH signal transduction pathways were identified and analyzed.

The results revealed that most of the genes that are associated with the 20E signaling pathway, such as *E74*, *E75* and *HR3,* were significantly up-regulated during metamorphosis, whereas most of the genes that are involved in the JH signaling pathway, such as *SRC* and *Kr-h1,* were significantly down-regulated (Tables 1 and 2). The expression levels of the 20E-related genes appeared to be higher than those of the JH-related genes. This implied that 20E signal is actively involved in the wing disc development during the larva-to-pupa metamorphosis. The assembled nucleotide sequences of transcripts (RNA-seq ID) in Tables 1 and 2 were provided in Additional files 3 and 4, respectively.

**Table 1:** **Changes in expression levels of the genes involved in the 20E signal transduction pathway**

Gene	RNA-Seq ID	L5D6-RPKM	PP-RPKM	P0-RPKM	L5D6 vs PP	PP vs P0
*USP* ^1^	Bm_nscaf2847_250	34.71	16.10	19.17	-	+
	Bm_nscaf2847_251	20.51	14.60	21.78	-	+
*ECR A*	Bm_nscaf2855_215	0.93	4.57	0.93	+	-
*ECR B2*	Bm_nscaf2855_217	21.95	2.09	8.55	-3.40*	+
*ECR B1*	Bm_nscaf2855_219	31.24	24.62	18.58	-	-
*E74* ^1^	Bm_nscaf2888_430	none	3.07	162.43	+	5.73*
	Bm_nscaf2888_433	19.63	5.34	64.70	-	3.60*
*E75B*	Bm_nscaf2859_001	45.67	100.29	168.69	-1.13*	+
*E75C*	Bm_nscaf2859_003	13.21	none	4.83	-	+
*HR3*	Bm_nscaf2964_066	2.03	268.66	4.91	7.05*	-5.78*
*BR-C A-NZ1*	Bm_nscaf2970_078	178.90	235.58	67.39	+	-
*BR-C Z4*	Bm_nscaf2970_079	71.94	122.93	38.45	+	-
*β FTZ-F1* ^1^	Bm_nscaf1690_227	4.32	9.94	none	+	-13.28*
	Bm_nscaf1690_228	8.48	7.36	1.71	-	-

^1^These genes have more than one RNA-Seq sequences. Gene expression level was calculated by RPKM method. "none" means that the expression of the gene was not detected. Asterisks indicate the gene, whose expression level had more than two folds difference between two stages and False Discovery Rate (FDR) was less than 0.001, was identified as differentially expressed at this developmental stage. "+" represents up-regulation, and "-" represents down-regulation. And the values in the last two columns were calculated by Log_2_(PP-RPKM/L5D6-RPKM) or Log_2_(P0-RPKM/PP-RPKM) and only those differentially expressed genes are given.

**Table 2:** **Changes in expression level of the genes involved in the JH signal transduction pathway**

Gene	RNA-Seq ID	L5D6-RPKM	PP-RPKM	P0-RPKM	L5D6 vs PP	PP vs P0
*Met1*	Bm_nscaf2828_039	45.41	11.65	8.92	-1.96*	-
*Met2* ^1^	Bm_nscaf1690_114	1.42	none	none	-	→
	Bm_nscaf1690_115	1.85	1.64	0.19	-	-
*SRC* ^1^	Bm_nscaf3078_04	none	3.35	none	+	-
	Bm_nscaf3078_05	8.99	10.38	3.22	+	-1.69 *
*Kr-h1*	Bm_nscaf2589_179	95.84	2.77	1.77	-5.11 *	-

^1^These genes have more than one RNA-Seq sequences. Expression level of the genes was calculated by RPKM method. "none" means that the expression of the genes was not detected. "+" represents up-regulation, "-" represents down-regulation, and "→" represents no change. Asterisks indicate the gene, whose expression level had more than two folds difference between two stages and False Discovery Rate (FDR) was less than 0.001, was identified as differentially expressed at this developmental stage. And the values in the last two columns were calculated by Log_2_(PP-RPKM/L5D6-RPKM) or Log_2_(P0-RPKM/PP-RPKM) and only those differentially expressed genes are given.

### Expression of transcription factors in the wing disc during metamorphosis

**Figure 4:**
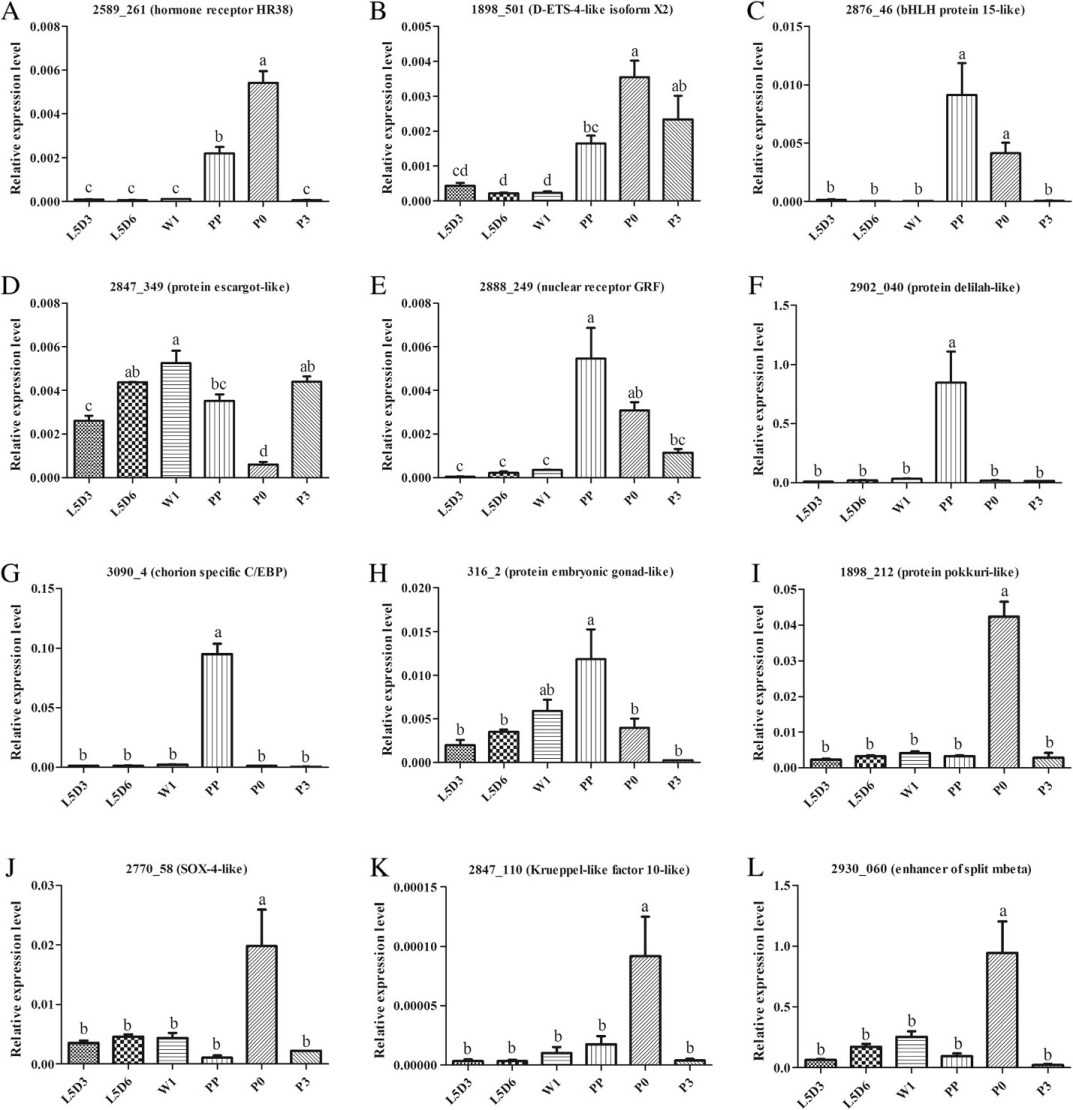
**Expression of the selected 12 transcription factors by qRT-PCR.** Expression patterns of the selected 12 transcription factors in the wing disc at indicated six developmental stages, which are 3-day-old fifth instar larvae (L5D3), L5D6, 1-day-old wandering (W1), PP, P0 and 3-day-old pupae (P3). **(A) Bm_nscaf2589_261** (probable nuclear hormone receptor HR38). **(B) Bm_nscaf1898_501** (D-ETS-4-like isoform X2). **(C) B** **m_nscaf2876_46** (bHLH protein 15-like). **(D) Bm_nscaf2847_349** (protein escargot-like). **(E) Bm_nscaf2888_249** (nuclear receptor GRF). **(F) Bm_nscaf2902_040** (helix-loop-helix protein delilah-like). **(G) Bm_nscaf3090_4** (chorion specific C/EBP). **(H) Bm_scaffold316_2** (protein embryonic gonad-like). **(I) Bm_nscaf1898_212** (ets DNA-binding protein pokkuri-like). **(J) Bm_nscaf2770_58** (transcription factor SOX-4-like). **(K) Bm_nscaf2847_110** (Krueppel-like factor 10-like). **(L) Bm_nscaf2930_060** (enhancer of split mbeta). The relative expression levels were normalized to the *Bmβ-actin* levels. The values are mean ± SEM (n = 3). Different letters indicate statistical significance (p < 0.05).

Transcription factors play critical roles in regulation of expression of their target genes. To identify transcription factors that may regulate the development of *B. mori* wing discs during metamorphosis, transcription factors were identified by KEGG analysis. In total, 147 out of 12,254 assembled transcripts were identified as transcription factors, 60 of which were differentially expressed during metamorphosis. Among the 60 differentially expressed transcription factors, 17 were up-regulated at PP and/or P0 stage (Table 3). Some of them were selected for confirmation by qRT-PCR analysis. The results showed that the transcription factors Bm_nscaf2589_261 (hormone receptor HR38), Bm_nscaf1898_501 (D-ETS-4-like isoform X2), Bm_nscaf2876_46 (bHLH protein 15-like), Bm_nscaf2888_249 (nuclear receptor GRF), Bm_nscaf2902_040 (protein delilah-like), Bm_nscaf3090_4 (chorion specific C/EBP) and Bm_scaffold316_2 (protein embryonic gonad-like) were significantly up-regulated at PP stage, which were well consistent with the RNA-Seq results (Table 3 and Figure 4A-C, E-H). The transcription factors Bm_nscaf1898_212 (protein pokkuri-like), Bm_nscaf2770_58 (SOX-4-like), Bm_nscaf2847_110 (Krueppel-like factor 10-like) and Bm_nscaf2930_060 (enhancer of split mbeta) were significantly up-regulated at P0 stage, which were also consistent with its RNA-Seq results (Table 3 and Figure 4I-L). The transcription factor Bm_nscaf2847_349 (protein escargot-like) was highly expressed at W1 stage, which was not consistent with the RNA-Seq result (Table 3 and Figure 4D). The qRT-PCR results showed that the RNA-Seq results were generally consistent. Bm_nscaf2859_001 (E75 isoform B), Bm_nscaf2964_066 (hormone receptor 3C) and Bm_nscaf2888_433 (transcription factor E74) were significantly up-regulated at PP or P0 stage (Table 3), which was consisted with the reported results [43, 44]. ASH2 was essential for scales formation in *B. mori* wing during pupal stage [45] and we found this transcription factor (Bm_nscaf1898_167) was significantly up-regulated at P0 stage. The assembled nucleotide sequences of transcripts (RNA-seq ID) in Table 3 were provided in Additional file 5, and primers for qRT-PCR were provided in Additional file 6.

**Table 3:** **Up-regulated transcription factors during the larva-to-pupa metamorphosis**

RNA-Seq ID	log _2_(PP/L5D6)	log _2_(P0/PP)	Gene description
**Bm_nscaf2589_261**	5.93*	1.43*	probable nuclear hormone receptor HR38
**Bm_nscaf1898_501**	2.55*		D-ETS-4-like isoform X2
Bm_nscaf2859_001	1.13*		E75 isoform B
**Bm_nscaf2876_46**	15.42*		bHLH protein 15-like
Bm_nscaf2734_12	6.05*	-6.63*	hepatic leukemia factor-like isoform X4
**Bm_nscaf2847_349**	1.77*	-2.46*	protein escargot-like
**Bm_nscaf2888_249**	8.43*	-1.46*	nuclear receptor GRF
**Bm_nscaf2902_040**	4.45*	-4.90*	helix-loop-helix protein delilah-like
Bm_nscaf2964_066	7.05*	-5.78*	hormone receptor 3C
**Bm_nscaf3090_4**	6.65*	-17.02*	chorion specific C/EBP
**Bm_scaffold316_2**	2.86*	-2.64*	protein embryonic gonad-like
Bm_nscaf1898_167		2.41*	achaete-scute-like protein ASH2
**Bm_nscaf1898_212**		3.88*	ets DNA-binding protein pokkuri-like
**Bm_nscaf2770_58**		2.82*	transcription factor SOX-4-like
**Bm_nscaf2847_110**		3.76*	Krueppel-like factor 10-like
Bm_nscaf2888_433		3.60*	transcription factor E74
**Bm_nscaf2930_060**		2.00*	enhancer of split mbeta

Asterisks indicate that the gene, whose expression level had more than two folds difference between two stages and False Discovery Rate (FDR) was less than 0.001, was identified as differentially expressed at this developmental stage. The values in the second and third columns were calculated by Log_2_(PP-RPKM/L5D6-RPKM) or Log_2_(P0-RPKM/PP-RPKM) and only those differentially expressed genes are given. Genes with black bold ID were selected for qRT-PCR analysis.

### Changes in expression level of cuticle protein genes in wing disc during metamorphosis

**Figure 5:**
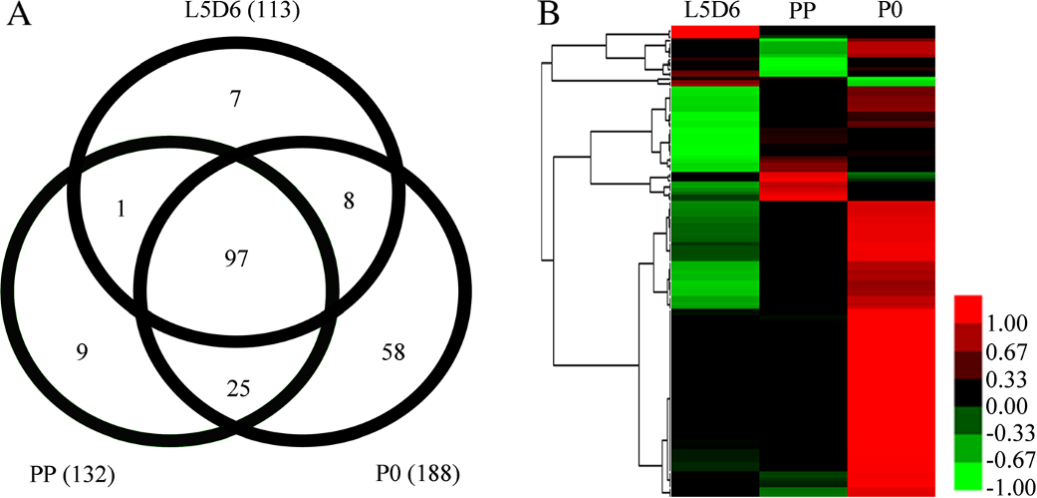
**Expression analysis of cuticular or cuticle protein coding genes.** The RPKM values of undetected genes are viewed as 0.001. Cluster 3.0 software was used to plot the heat map (Similarity Metric: Euclidean distance, Clustering method: Complete linkage), and Treeview software was used to generate the heat map. The colors in the map display the relative values at the given 3 developmental stages. Green indicates the lowest level of expression, black indicates the intermediate level of expression, and red indicates the highest level of expression. **(A)** Distribution of cuticular or cuticle protein coding genes at the three stages. **(B)** Heat map of hierarchical clustering of 148 differentially expressed cuticle protein genes.

Cuticle proteins are important component of wing disc in the post-larva development. To study the relationship between expression of cuticle proteins and wing development, cuticle protein genes were analyzed. Totally 205 cuticle protein transcripts were identified using Blast search. Ninety-seven transcripts were expressed at all the three stages, while 7, 9 and 58 transcripts were specifically expressed at L5D6, PP and P0 stage respectively (Figure 5A). One hundred forty eight out of 205 transcripts were differentially expressed, with most of them being up-regulated during the larva-to-pupa metamorphosis, particularly from PP to P0 stage (Figure 5B). This result was consistent with the results by microarray analysis [36]. By similarity analysis of sequences, 10 WCPs genes were identified in our RNA-Seq dataset. Expression of *WCP1a*, *WCP1b*, *WCP3*, *WCP4*, *WCP5*, *WCP8* and *WCP9* was slightly up-regulated at the PP stage and then greatly increased at the P0 stage. Expression of *WCP10* was slightly up-regulated at the PP stage and then greatly decreased at the P0 stage, whereas the expression of *WCP11* was decreased at the PP stage and then greatly increased at the P0 stage (Table 4).

**Table 4:** **Expression level of WCP genes**

WCPs	RNA-Seq ID	L5D6-RPKM	PP-RPKM	P0-RPKM
WCP1a	Bm_nscaf1681_169	2.72	3.76	5361.40
WCP1b	Bm_nscaf1681_171	1.10	72.00	4902.09
WCP2	Bm_nscaf1681_167	1.35	29.18	6441.87
WCP3	Bm_nscaf1681_163	3.68	176.15	16652.38
WCP4	Bm_nscaf1681_161	none	7.91	744.39
WCP5	Bm_nscaf1681_160	21.79	88.31	48948.29
WCP8	Bm_nscaf1681_192	2.38	161.58	10340.63
WCP9	Bm_nscaf1681_048	19.65	143.49	15882.70
WCP10	Bm_nscaf2136_095	437.87	699.39	17.69
WCP11	Bm_nscaf1681_049	487.93	1.05	197.21

The gene expression level was calculated by RPKM method. "none" means that the expression of the gene was not detected.

### Change in expression level of chitin degradation and synthesis related genes

**Figure 6:**
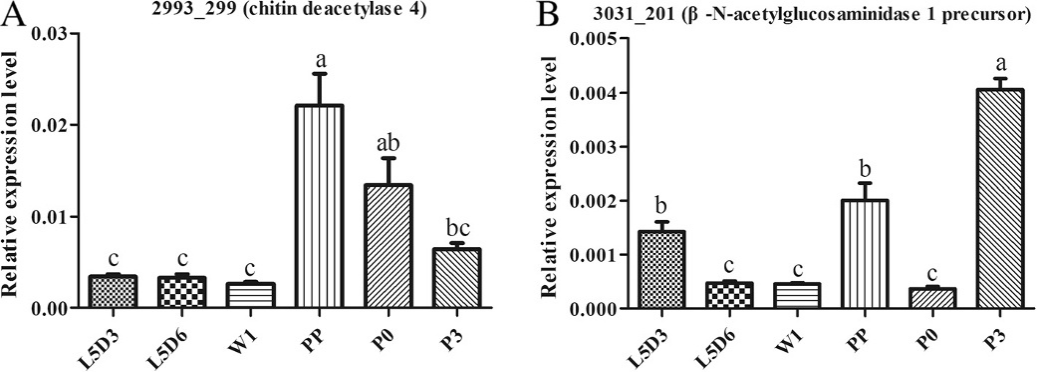
**qRT-PCR results of expression of the chitin degradation related genes.** The expression patterns of two chitin degradation genes in the wing disc at six developmental stages, which are 3-day-old fifth instar larvae (L5D3), L5D6, 1-day-old wandering (W1), PP, P0 and 3-day-old pupae (P3) The relative expression levels were normalized to the *Bmβ-actin* levels. **(A) Bm_nscaf2993_229** (chitin deacetylase 4). **(B) Bm_nscaf3031_201** (β-N-acetylglucosaminidase 1 precursor). The values are the mean ± SEM (n = 3) of three repeat experiments using qRT-PCR. Different letters indicate statistical significance (p < 0.05).

**Figure 7:**
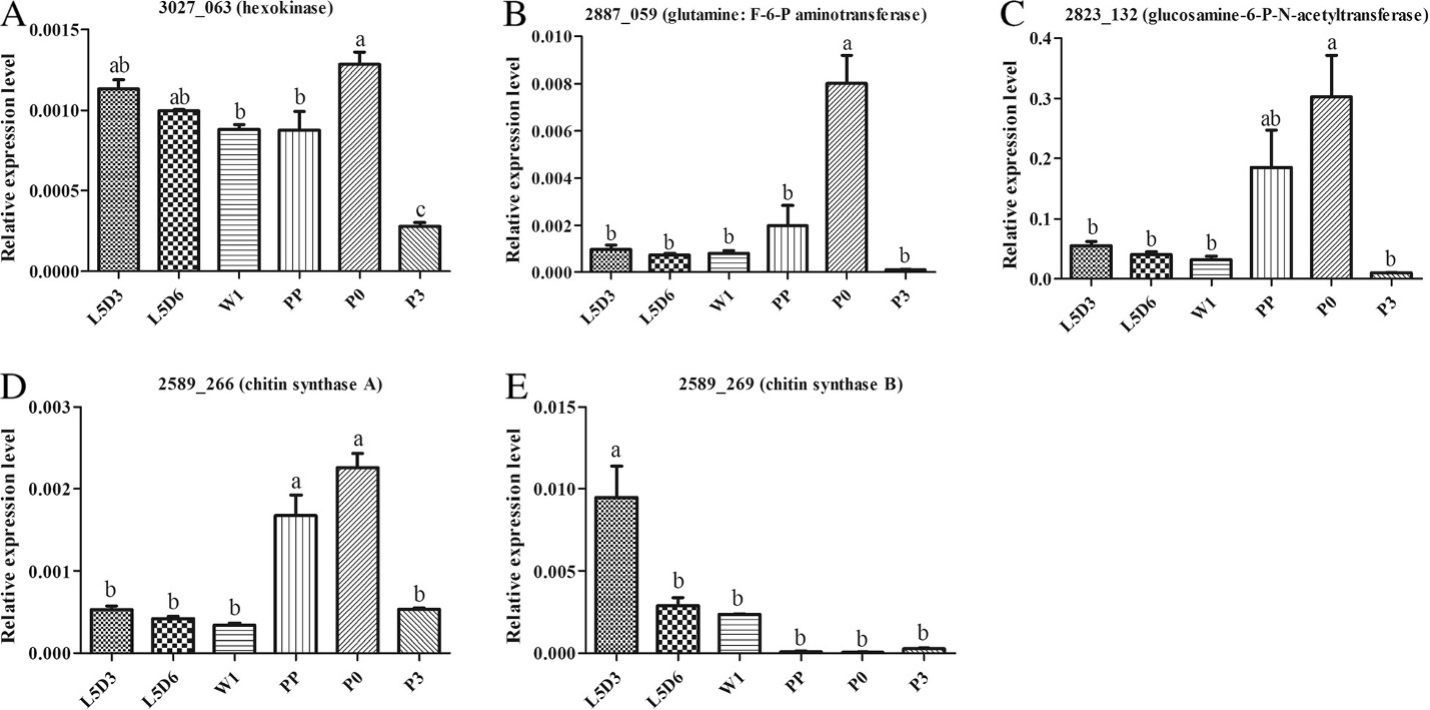
**qRT-PCR results of 5 genes relevant to chitin synthesis.** The expression patterns of five selected chitin synthesis related genes in the wing disc at six developmental stages. which are 3-day-old fifth instar larvae (L5D3), L5D6, 1-day-old wandering (W1), PP, P0 and 3-day-old pupae (P3). **(A) Bm_nscaf3027_063** (hexokinase type 2-like isoform X3). **(B) Bm_nscaf2887_059** (glutamine: fructose-6-phosphate aminotransferase). **(C) Bm_nscaf2823_132** (glucosamine-6-phosphate N-acetyltransferase). **(D) Bm_nscaf2589_266** (chitin synthase A). **(E) Bm_nscaf2589_269** (chitin synthase B). The relative expression levels were normalized to the *Bmβ-actin* levels. The values are the mean ± SEM (n = 3) of three repeat experiments using qRT-PCR. Different letters indicate statistical significance (p < 0.05).

To study the dynamic of chitin in wing disc during the pupal wing transition, the genes that are involved in chitin degradation and synthesis pathways were investigated. Most of the genes that are related to chitin degradation were significantly up-regulated at the PP stage and the genes that are related to chitin synthesis were also up-regulated at the PP or P0 stage (Tables 5 and 6). Two critical chitin degradation related genes [Bm_nscaf2993_299 (chitin deacetylase 4) and Bm_nscaf3031_201 (β-N-acetylglucosaminidase 1 precursor)] were significantly up-regulated at the PP stage, then down-regulated at the P0 stage (Figure 6A, B), which were consistent with the results of RNA-Seq (Table 5). Chitin synthesis related genes including Bm_nscaf3027_063 (hexokinase), Bm_nscaf2887_059 (glutamine:F-6-P aminotransferase), Bm_nscaf2823_132 (glucosamine-6-P-N-acetyltransferase), Bm_nscaf2589_266 (chitin synthase A) were significantly up-regulated at the PP or P0 stage, and Bm_nscaf2589_269 (chitin synthase B) was significantly down-regulated from L5D3 to P3 stage (Figure 7A-E), which were generally consistent with the results of RNA-Seq (Table 6). The assembled nucleotide sequences of transcripts (RNA-seq ID) in Tables 5 and 6 were provided in Additional files 7 and 8, respectively. Primers for qRT-PCR were provided in Additional file 6.

**Table 5:** **Expression level of the chitin degradation related genes**

RNA-Seq ID	L5D6-RPKM	PP-RPKM	P0-RPKM	Gene description
Bm_nscaf2912_25	44.80	5923.77	246.74	chitinase
Bm_nscaf2986_109	45.50	13836.70	477.85	chitinase precursor
Bm_nscaf2829_151	82.40	230.91	246.51	chitinase 3-like isoform X1
Bm_nscaf2829_153	52.70	157.91	173.42	chitinase
**Bm_nscaf2993_229**	28.70	156.08	100.65	chitin deacetylase 4
Bm_nscaf2993_230	44.40	184.62	142.59	chitin deacetylase 4
**Bm_nscaf3031_201**	3.87	186.23	14.43	β-N-acetylglucosaminidase 1 precursor
Bm_nscaf463_07	1.86	none	0.37	β-N-acetylglucosaminidase 2 precursor
Bm_nscaf463_08	0.35	0.34	none	β-N-acetylglucosaminidase 3 precursor

The gene expression level was calculated by RPKM method. "none" means that the expression of the gene was not detected. The genes with black bold ID were selected for qRT-PCR analysis.

**Table 6:** **Expression levels of the chitin synthesis related genes**

RNA-seq ID	L5D6-RPKM	PP-RPKM	P0-RPKM	Gene description
Bm_nscaf2800_23	20.24	12.65	8.76	trehalase-2
Bm_nscaf2800_24	11.56	9.11	13.95	trehalase-2
Bm_nscaf2829_133	0.21	none	1.28	trehalase precursor
Bm_nscaf2829_134	0.42	1.88	none	trehalase 1B
Bm_nscaf3027_062	6.90	15.30	8.68	hexokinase type 2-like isoform X3
**Bm_nscaf3027_063**	47.91	105.58	100.81	hexokinase type 2-like isoform X3
Bm_nscaf2780_10	122.98	145.82	36.81	glucose-6-phosphate isomerase
**Bm_nscaf2887_059**	53.54	51.33	675.08	glutamine:fructose-6-phosphate aminotransferase
**Bm_nscaf2823_132**	77.53	155.12	886.62	glucosamine-6-phosphate N-acetyltransferase
Bm_nscaf3003_158	13.61	56.53	34.73	phosphoacetylglucosamine mutase
Bm_nscaf2176_247	17.11	14.13	436.84	UDP-N-acetylhexosamine pyrophosphorylase
**Bm_nscaf2589_266**	49.30	138.56	277.03	chitin synthase A
**Bm_nscaf2589_269**	3.55	0.27	none	chitin synthase B
Bm_nscaf2589_270	5.42	none	none	chitin synthase B

The gene expression level was calculated by RPKM method. "none" means that the expression of the gene was not detected. The genes with black bold ID were selected for qRT-PCR analysis.

*De novo* synthesis of chitin in insects usually utilizes trehalose as the starting material. However, base on the pathway map of chitin synthesis (KEGG pathway map: ko00520), D-fructose can also be used for chitin *de novo* synthesis. In this study, high performance liquid chromatography (HPLC) was used to determine the levels of trehalose, D-glucose and D-fructose in the hemolymph of the silkworm at L5D6, PP and P0 stage. The results showed that trehalose, D-glucose and D-fructose were presented with high concentrations at L5D6 stage, and then their concentrations rapid decreased at the PP stage (Table 7). The concentrations of the three sugars were slightly decreased from the PP to P0 stage (Table 7). These results imply that the rapid use of the three sugars in the hemolymph might be taking place from L5D6 to PP stage.

**Table 7:** **Concentrations of trehalose, D-glucose and D-fructose in the hemolymph at three stages of** ***B. mori***

Sample	Trehalose (mg/ml)	D-glucose (mg/ml)	D-fructose (mg/ml)
hemolymph of L5D6	5.14	0.33	0.51
hemolymph of PP	2.71	0.27	0.40
hemolymph of P0	2.56	0.23	0.39

### Changes in the expression levels of wing disc development related genes

Expression patterns of the genes homologous to *Drosophila* wing early development were also analyzed in this study. The results showed that most of the genes, such as *En*, *Ap*, *Hh*, reported to be important for embryonic and early development of *Drosophila* wing disc were significantly decreased during metamorphosis (Table 8). However, *Fng* that was necessary for *B. mori* wing development during larvae to pupae metamorphosis [21], *Ash2* that was vital for scales formation of *B. mori* wing during pupal stage [45], and *urbain*, an unknown gene that was up-regulated at PP stage in *B. mori* wing [46], were also significantly up-regulated at the PP or P0 stage in this study. The assembled nucleotide sequences of transcripts (RNA-seq ID) in Table 8 were provided in Additional file 9.

**Table 8:** **Expression patterns of the genes related to wing disc early development**

Gene	RNA-seq ID	L5D6-RPKM	PP-RPKM	P0-RPKM	L5D6 vs PP	PP vs P0
*engrailed*	Bm_nscaf2964_172	57.41	27.17	25.47	-1.08*	-
*Hedgehog*	Bm_nscaf3048_40	13.70	5.56	3.24	-	-
*decapentaplegic*	Bm_nscaf2993_221	38.81	22.75	17.10	-	-
*apterous AE*	Bm_nscaf2210_128	117.49	57.39	27.28	-1.03*	-
*apterous AF*	Bm_nscaf2210_130	31.10	27.52	8.94	-	-1.62*
*apterous Bβ*	Bm_nscaf2210_132	95.88	83.98	16.08	-	-2.38*
*Fringe* ^*1*^	Bm_nscaf2860_94	13.82	40.86	15.17	1.56*	-1.43*
	Bm_nscaf2860_96	45.94	74.37	28.61	+	-1.37*
*delta*	Bm_nscaf2986_021	12.75	6.39	3.16	-	-
*serrate*	Bm_nscaf1705_01	83.35	35.30	18.98	-1.24*	-
*Notch* ^*1*^	Bm_nscaf2888_347	9.04	4.05	3.31	-	-
	Bm_nscaf2888_348	5.91	4.25	9.16	-	+
	Bm_nscaf2888_349	17.65	14.18	7.54	-	-
*Wingless*(precursor)	Bm_nscaf2847_167	17.20	5.51	0.86	-1.64*	-
*vestigial*	Bm_nscaf3056_42	17.08	7.54	7.70	-	+
*scalloped*	Bm_nscaf1898_116	52.42	25.22	14.10	-1.06*	-
*Scalloped isoform 2*	Bm_nscaf1898_117	44.05	35.26	8.31	-	-
*achaete-scute 2*	Bm_nscaf1898_167	2.04	7.54	40.06	+	2.41*
*urbain*	Bm_nscaf2902_362	45.40	11497.96	161.44	7.98*	-6.15*

^1^These genes have more than one RNA-Seq sequences. The gene expression level was calculated by RPKM method. "+" represents up-regulation, and "-" represents down-regulation. Asterisks indicate that the gene, whose expression level had more than two folds difference between two stages and False Discovery Rate (FDR) was less than 0.001, was identified as differentially expressed at this developmental stage. And the values in the last two columns were calculated by Log_2_(PP-RPKM/L5D6-RPKM) or Log_2_(P0-RPKM/PP-RPKM) and only those differentially expressed genes are given.

## Discussion

Insect metamorphosis is mediated by 20E and JH signals. During larval stage, JH exhibits its "status quo" function probably through two transcription factors, E75A and Kr-h1, to prevent the activation of *BR-C*, a 20E signal transduction gene for larvae-pupae transition [7, 47]. Though a relative high titer of JH was present in hemolymph of *B. mori* at the end of the final instar larvae [48], the JH does not inhibit the wing disc transformation. Some studies implied that the success of this transition was attributed to the loss of sensitivity of JH signal in *B. mori* wing disc at the end of the instar larvae [19, 20]. However, the mechanism for the JH insensitiveness in the wing disc is not revealed yet. In this study, during the larva-to-pupa metamorphosis, 20E signal transduction pathway related genes, such as *E74, E75* and *HR3*, were significantly up-regulated in *B. mori* wing disc. However JH signal transduction pathway related genes, such as *Met, SRC* and *Kr-h1*, were significantly down-regulated and their expression levels were relatively low. High expression of *BR-C* was detected in *B. mori* wing disc, while the expression of *E75A* was not detectible before pupation (Tables 1 and 2). These results implied that the loss of sensitivity to JH is probably due to the low expression of JH-related genes during larvae-to-pupae metamorphosis, resulting that the *B. mori* wing disc can be transformed to pupal wing despite high JH titer in the hemolymph before pupation. However what causes the inactive expression of transmitters of JH signaling during metamorphosis has not been revealed yet.

Out of the 17 up-regulated transcription factors, 10 were significantly up-regulated at the PP stage (Table 3). These genes may be involved in positive regulation of the transition of wing disc to pupal wing. In *Drosophila*, HR38 was involved in regulating the expression of cuticle protein genes during pupal stage [49]. Cuticle protein genes were actively transcribed in *B. mori* wing at P0 stage (Figure 5B), so HR38 might be involved in modulating their expression. Previous study indicated that GRF might be a cofactor of βFTZ-F1 and HR39 [50]. βFTZ-F1 was reported to have an essential role in regulating the expression of cuticle protein [51, 52]. In this way, the transcription factor GRF may be positively involved in regulating the expression of cuticle protein, which induces the growth of the wing disc during the larva-to-pupa transformation. Transcription factors HR3 and E75 were involved in 20E signal transduction and they were found to be capable of binding with the response element of many cuticle protein genes [12, 53]. Therefore, they may also be involved in regulating the expression of cuticle proteins in the wing disc during metamorphosis. In *Drosophila*, *gonad,* also known as *Eagle*, is important for the development of nervous system, and *eg*^*MZ360*^/*eg*^*MZ360*^ (a partial loss-of-function allele) *Drosophila* showed a held-out wing phenotype and lost the ability of flying [54, 55], implying that *Eagle* may be involved in the normal development of wing. C/EBP transcription factor was reported to have a role in regulating chorion gene expression in silkmoth, however, its role in wing disc development has not been studied yet [56]. *Delilah* is a marker gene for epidermal muscle attachment site in *Drosophila* and may play a role in epidermal muscle attachment site development [57, 58], but its role for *B. mori* wing development has not been clarified yet.

Out of the 17 transcription factors, except for the PP up-regulated transcription factors, six transcription factors were up-regulated only at the P0 stage (Table 3) which may play a role in pupal wing development in *B. mori*. For example, ASH2 is required for normal formation of wing scales in *B. mori* during pupal stage [45]. Transcription factor Pokkuri is also known as Yan and Aop in *Drosophila*. Yan is regulated by epidermal growth factor (EGF). Ectopic expression of Yan in the A-P boundary of *Drosophila* wing disc can lead to the mis-expression of *Vein*, a gene involved in normal development of wing vein and the small wing, and result in the loss of bristles phenotype [59, 60]. This implies that Pokkuri is involved in wing development, but the exact role of Pokkuri in *B. mori* wing development is still uncovered. SOX-4, a HMG-box transcription factor, is an activator in regulating the expression of neuronal gene and necessary for neuronal maturation [61]. Nervous system can be found in wing vein and the up-regulated *SOX-4* at P0 stage may be involved in regulating the neuronal development in *B. mori* wing vein. Pupal wing of *B. mori* has reached to the proper size of its adult wing. Krueppel-like factor 10 is an anti-proliferative factor in mammals [62]. It is possible that when *B. mori* wing disc transforms to pupal wing, cell proliferation is negatively regulated by Krueppel-like factor 10, resulting in the arrest of wing growth when it reaches the proper size. E74 is an early response gene in 20E signaling pathway and is involved in the expression regulation of many cuticle protein genes [12, 52]. Its high level of expression in the wing disc suggests its potential role in regulating the expression of cuticle protein during the wing development. Study in *Drosophila* suggests that Enhancer of split mbeta [E(spl)mβ] has a function to limit the expression of *Rho* (*Rhomboid*), a vein marker gene, in the *Drosophila* wing vein [59, 63]. In *B. mori*, wing veins are optimized during pupal stage. Therefore, E(spl)mβ may play similar role as in *Drosophila*. Functions of the other up-regulated transcription factors remain unknown.

GO classification analyses indicated that the significantly up-regulated transcripts are dominant in structural molecule GO item (Figure 3A and 3B). Actually, cuticle protein genes have high expression levels during the transition of from larval wing disc to pupal wing (Figure 5B and Table 4). Cuticle protein genes were highly expressed at the P0 stage and their RPKM value accounted for about 45.5% of RPKM from all genes (see Additional file 10). These results implied that the large amount of cuticle proteins are synthesized, which is one of the main events related to morphological changes in wing, during larva-to-pupa metamorphosis. In addition, among the 13 *WCPs* genes identified in *B. mori*, *WCP 1–9* were highly expressed around pupation, while *WCP10* and *WCP11* have high expression levels before pupation and then significantly down-regulated during pupation [29–31]. In this study, 10 WCPs can be found and their expression patterns are similar to previous studies (Table 4). These results implied that WCP1-9 are the main cuticle proteins for pupal wing, while WCP10 and WCP11 are main cuticle proteins for wing disc in *B. mori*.

As insects metamorphosize from larvae to pupae, old integument of larvae is degraded and replaced by pupal integument. Wing disc undergoes rapid growth and differentiation during metamorphosis, evaginating to form pupal wing. No cuticle is degraded in *B. mori* wing disc during the transition from the wing disc to pupal wing. In this study, relevant genes for both chitin degradation and *de novo* synthesis were significantly up-regulated (Tables 5 and 6 and Figures 6 and 7). This implied that the pupal wing chitin may come from both chitin degradation and *de novo* synthesis. Chitin deacetylases can catalyze chitin to form chitosan and had a function in organizing tracheal tube length in *Drosophila* [64, 65]. Chitin deacetylases had higher expression in wing disc at PP stage in *B. mori*. Whether it involves in extending of tracheal tube in wing is still unknown. In *B. mori*, there are two kinds of chitin synthase, A and B. While synthase A is responsible for the chitin synthesis in the epidermis, synthase B is responsible for the chitin synthesis in the gut [28]. However it is not clear which synthase is involved in chitin synthesis in insect wing. In this study, synthase A was significantly up-regulated in wing disc during metamorphosis, while the expression of synthase B was almost undetectable (Table 6 and Figure 7D, 7E). This result suggested that the chitin synthesis in pupal wing is majorly performed by synthase A. In *Drosophila* and *Tribolium*, Knickkopf (Knk) and Retroactive (Rtv) are essential for chitin synthesis and always along with the expression of chitin synthase A [66, 67]. In Lepidopterans, these two factors had not been studied so far. But Bm_nscaf3003_024 (protein Skeletor, isoforms D/E-like), the homolog of Knk, was found to be significantly up-regulated at PP stage in our data (Additional file 1), whose expression pattern was consistent with that of chitin synthase A (Figure 7D), implying that Protein Skeletor may have similar function as Knk did in *Drosophila* and *Tribolium*. The homolog of Rtv was not detected in our data.

In addition, D-fructose can be transformed to D-fructose 6-phosphate by hexokinase, which can be used by chitin synthesis pathway (KEGG pathway map: ko00520). Trehalose, D-glucose and D-fructose were detected in *B. mori* hemolymph, and their amounts rapidly decreased from L5D6 to PP (Table 7). This suggests that the rapid *de novo* synthesis of chitin might be taken place in the wing during transition from L5D6 to PP, and both trehalose and D-fructose may be used in *de novo* synthesis of chitin in the wing of *B. mori*.

Early gene *Fng* was also necessary for the *B. mori* wing development during larva-to-pupa metamorphosis [5, 21, 68]. To investigate other early function genes, expression patterns of these genes were analyzed during metamorphosis in *B. mori* (Table 8). Results showed that most of them, including *Ap* and *En*, which were reported to play important roles in cell fate determination in *Drosophila* wing disc during early developmental stages, were significantly down-regulated in *B. mori* wing during metamorphosis, implying that cell fate determination of *B. mori* wing disc may be decided before pupation.

## Conclusion

In this work, total 12,254 assembled transcripts were obtained from the wing disc at L5D6, PP and P0 stages. Totally 5,287 differentially expressed assembled transcripts were identified. From L5D6 to PP (L5D6 vs PP) 2,778 transcripts were differentially expressed and from PP to P0 (PP vs P0) 3,864 transcripts were differentially expressed. More down-regulated transcripts than up-regulated transcripts were identified during the transition from L5D6 to PP and from PP to P0. The transition of wing disc to pupal wing appeared under the control of the 20E signal transduction pathway because high levels of the transcription factors related to 20E signal transduction pathway were found during larva-to-pupa metamorphosis. Seventeen up-regulated transcription factors were screened out, and their functions on *B. mori* wing development were predicated. High levels of the expression of cuticle proteins and chitin related genes reflect the active chitin metabolism related to the morphological and structural changes during the metamorphosis from the wing disc to pupal wing. Chitin source of pupal wing may come from both degradation of componential chitin and *de novo* synthesis. Chitin synthase A might be responsible for the chitin synthesis in *B. mori* pupal wing. *De novo* synthesis of chitin initiated from D-fructose may exist in *B. mori* wing.

## Methods

### Experimental animal and RNA isolation

Silkworm, *B. mori*, were reared with mulberry leaves at 27°C under a 12 h light/12 h dark cycle. Under this condition, the fifth instar stage lasted about 7 days; the wandering stage (W) lasted about 3 days and pupal stage lasted about 10 days. The stage between the end of larval spinning and the onset of pupation was defined as prepupae (PP), which lasted about 12 hours, equal to the period from 2.5-day-old wandering to 3-day-old wandering. P0 was defined as the stage when *B. mori* just finished pupation. *B. mori* wing discs were clearly dissected from larvae or pupae at stage of L5D6, PP and P0. Total RNA was isolated by using Rneasy Kit (QIAGEN) according to the manufacturer’s instructions.

### RNA-seq sequencing and *de novo*assembly of sequences

Five μg of total RNA from L5D6, PP and P0 wing was prepared, respectively, for performing RNA-seq sequencing analysis by using Ilumina HiSeq™ 2000 platform. The clean reads of each gene detected in RNA-seq sequencing analysis was assembled by using Trinity software (*de novo* assembly) [40] and the assemble results were provided in supplemental files.

### Calculation of gene expression level and annotation of predicted proteins

Expression level of genes is calculated by using RPKM (Reads Per kb per Million reads) method [69], and the formula is:


Here C means the number of reads that uniquely aligned to target gene; N means the total number of reads that uniquely aligned to all genes; L means the number of base on target gene.

Genes were annotated by using Blast2go software ( http://www.blast2go.com/b2ghome) according to the translated nucleotide sequences in Blastp.

### Isolation and analysis of differentially expressed genes

To compare the differentially expressed genes, the transcripts of PP were compared with those of L5D6 (L5D6 as control), and the transcripts of P0 were compared with those of PP (PP as control). Those genes that had two fold difference between two stages and False Discovery Rate (FDR) was less than 0.001 [70] were identified as differentially expressed genes. The detailed information of their expression patterns can be obtained in the supplemental files (Additional files 1 and 2).

The Cluster 3.0 and Treeview software ( http://www.eisenlab.org/eisen/) were used to analyze the expression pattern of differentially expressed genes. Kyoto Encyclopedia of Genes and Genomes (KEGG: http://www.kegg.jp/) [71] was used to identify transcription factors. BGI Web Gene Ontology Annotation Plotting (WEGO) ( http://wego.genomics.org.cn/cgi-bin/wego/index.pl) [72] were used to classify genes to different GO catalogs and Pearson Chi-Square test was used to justify if the gene numbers of the two input dataset were significantly different.

### Quantitative real time -PCR (qRT-PCR)

RNAiso Plus (Takara Dalian, China) was used to isolate total RNA from *B. mori* wing disc from 3-day-old fifth instar larvae (L5D3), L5D6, 1-day-old wandering (W1), PP, P0 and 3-day-old pupae (P3), respectively. Two μg of total RNAs from each stage was used to synthesize cDNA using reverse transcriptase (Takara) and oligo d (T) primer (Takara). The total volume of real-time quantitative PCR reactions was 20 μl, which contained 10 μl of 2 × SYBR^®^ Premix EX Taq™ (Takara), 0.4 μl of 50 × ROX Reference Dye (Takara) and 0.4 μl of specific primers (10 μM). Detail information of qRT-PCR Primers were provided in Additional file 4. qRT-PCR was performed with an ABI7300 real-time PCR system (Applied Biosystems, Foster City, CA), using the following condition: 95°C for 30 s followed by 40 cycles in 95°C for 5 s, 60°C for 30 s and 72°C for 31 s. The mRNA quantity of each gene was calculated with the 2^-△△Ct^ method and normalized to the abundance of a house-keeping gene, *Bmβ-actin* (GenBank NO: EU780706.1). The relative mRNA levels of each gene were represented as folds over the expression levels of *Bmβ-actin*.

### Hemolymph collection and sugar detection

The hemolymph of *B. mori* was collected by cutting its proleg (L5D6 and PP stages) or pupal wing (P0 stage), and quickly transformed to centrifuge tube by pipette. All hemolymph samples were stored at -80°C, until performing the sugar detection. The sugar detection was performed by China National Analytical Center (Guangzhou) by HPLC method.

### Availability of supporting data

The assembled unigenes (larger than 200 bp) were deposited in the Transcriptome Shotgun Assembly Sequence Database (TSA) at DDBJ/EMBL/GenBank under the accession GBJR00000000. The version described in this paper is the first version, GBJR01000000.

## Electronic supplementary material

Additional file 1: Table S1: Differentially expressed transcripts in L5D6-VS-PP. The criteria applied for significance difference were FDR ≤ 0.001, and estimated absolute |log2Ratio (PP/L5D6)| ≥ 1. L5D6- and PP-RPKM: reads per kb per million reads for each transcript in stages L5D6 and PP, respectively. Log2 Ratio (PP/L5D6): the ratio between the RPKM in PP and the RPKM in L5D6. Up-Down (PP/L5D6) and FDR of each gene are also shown. GO Component, GO Function and Go Process: ontology information of Cellular Components, Molecular Function and Biological Processes of Gene-corresponding GO terms. "-": no hit. Blast nr: identification of homologues in GenBank; E-values are also shown. (XLS 980 KB)

Additional file 2: Table S2: Differentially expressed transcripts in PP-VS-P0. Description of data is similar to Table S1. Log2 Ratio (P0/PP): the ratio between the RPKM in P0 and the RPKM in PP. (XLS 1 MB)

Additional file 3: **Assembled nucleotide sequences of transcripts in Table 1.** (DOC 36 KB)

Additional file 4: **Assembled nucleotide sequences of transcripts in Table 2.** (DOC 32 KB)

Additional file 5: **Assembled nucleotide sequences of transcripts in Table 3.** (DOC 42 KB)

Additional file 6: Table S3: Primers for qRT-PCR. (XLS 21 KB)

Additional file 7: **Assembled nucleotide sequences of transcripts in Table 5.** (DOC 34 KB)

Additional file 8: **Assembled nucleotide sequences of transcripts in Table 6.** (DOC 44 KB)

Additional file 9: **Assembled nucleotide sequences of transcripts in Table 8.** (DOC 44 KB)

Additional file 10: Table S4: All transcripts at P0 stage. All transcripts at stage P0 were listed. Description of data is similar to Table S1. (XLS 3 MB)

## Abbreviations

L5D3: 3-day-old fifth instar larvae
W1: 1-day-old wandering
P3: 3-day-old pupae
L5D6: 6-day-old fifth instar larvae
PP: Prepupae
P0: Pupae
20E: 20-hydroxyecdysone
JH: Juvenile hormone
EcR: Ecdysone receptor
USP: Ultraspiracle
BR-C: Broad complex
Met: Methoprene-tolerant
Kr-h1: Krüppel homolog 1
SRC: Steroid receptor coactivator
En: Engrailed
Ap: Apterous
Sd: Scalloped
Vg: Vestigial
Hh: Hedgehog
Dpp: Decapentaplegic
Wg: Wingless
Fng: Fringe
Egfr: Epidermal growth factor receptor
N: Notch
Ser: Serrate
Dl: Delta
WCP: Wing cuticle protein
GO: Gene ontology
RPKM: Reads Per kb per Million reads
FDR: False Discovery Rate
KEGG: Kyoto Encyclopedia of Genes and Genomes
WEGO: BGI Web Gene Ontology Annotation Plotting.
