# Supplementary material for: Transcriptomic analysis of developmental features of Bombyx mori wing disc during metamorphosis
Source: BMC Genomics. 2014 Sep 27;15(1):820. doi: 10.1186/1471-2164-15-820 (PMC4196006; doi:10.1186/1471-2164-15-820)
Supplement: Supplementary file 3 — Additional file 3: Assembled nucleotide sequences of transcripts in Table 1. (DOC 36 KB) [file 12864_2014_6525_MOESM3_ESM.doc]

**>Bm_nscaf2847_250**

ATGGGTGTCGTGTTTCAAAGCGGGAGACGGCATTTGCATGACAAGGGCTTGGCTCTGGCTTATCACCAGGAGAGTCGTGAGCTTGTTGAAATTTACGGCAACAATAGAGATAGGGGTTGTGAAGGCTGCAAAGGGTTCTTCAAGAGGACAGTCAGGAAGGATCTCACATACGCGTGCCGGGAGGACAAGAATTGTATAATAGATAAACGCCAGCGGAATCGTTGCCAGTACTGCCGATACCAGAAGTGTCTCGCTTGCGGCATGAAGAGGGAGGCTGTGCAAGAGGAGAGACAGCGAGCCGCGAGGGGTACAGAAGACGCTCATCCCAGCAGCTCTGTACAGGAGCTATCGATCGAGCGGCTGCTGGAATTGGAGGCGTTAGTTGCGGATTCAGCTGAGGAGTTACAGATCCTACGCGTCGGTCCCGAAAGCGGCGTGCCGGCCAAGTACCGAGCTCCCGTTTCGAGTCTTTGTCAAATAGCTATTTTGGGAGTATGGACTATAAATTTGGAGTTTCGAGCATGGTGTAAACTGAATTGA

**>Bm_nscaf2847_251**

ATGTTTTCAGGCAACAAACAGATAGCCGCTCTCATTGTTTGGGCGCGTGACATTCCACACTTCGGGCAGCTAGAAATCGACGATCAGATCCTTCTAATCAAGGGCTCCTGGAACGAACTGCTGCTGTTCGCTATCGCATGGCGGTCTATGGAGTTCCTCAATGATGAACGAGAGAACGTAGACTCGCGGAATACGGCGCCGCCTCAACTCATTTGTTTAATGCCAGGCATGACGCTGCACCGCAACTCCGCGCTGCAGGCCGGCGTGGGGCAGATCTTCGACCGCGTGCTCTCCGAGCTGTCGCTCAAGATGCGCTCCCTCCGCATGGACCAGGCCGAGTACGTCGCGCTCAAGGCCATCATACTCCTCAATCCTGACGTAAAAGGATTGAAGAATAAACAAGAAGTGGACGTTCTTCGAGAAAAGATGTTCTTATGCCTGGACGAGTACTGCCGGCGCTCGCGCGGCGGGGAGGAGGGTCGGTTCGCGGCGCTGCTGCTGCGGCTGCCGGCGCTGCGCTCCATCTCGCTCAAGAGCTTCGAGCACCTCTACCTGTTCCACCTCGTGGCCGAGGGCAGCGTGAGCTCGTACATCCGCGACGCGCTCTGCAACCACGCGCCGCCCATCGACACCAACATCATGTAG

**>Bm_nscaf2855_215**

ATGATGATCAAGCGCGTAATGATGATGTACAAGGCGCCGGCAGAAGTGTGGAGTGCGGGCGGCGGGGCCGGCGGTGGGACAGCATGCGGTAGCACCGGCATGGAGCTGAAACACGAGGTGGCGTACCGCGGCGTGCTGCCCGGCCAGGTGAAGGCCGAGCCTGGCGTCAGTCACAACGGCCATCCGGTCAACGGACACGTCCGGGACTGGATGGCGGGGGGAGCTGCGGGCGGGGGCTCGCCCTCCCCAGGCGCGCCGGGACAACCGCAGCCCAGCAACGGATATTCGTCGCCACTATCCTCAGGCAGCTACGGTCCGTACAGCCCTAATGGAAAAATAGCGGGGAATCGGAACAACAACAGTAATCTCATAGCGAATGCACGACCCACATGCTGA

**>Bm_nscaf2855_217**

ATGAGAGTCGAGAACGTGGATAACGTATCGTTTGCTTTGAACGGACGCGCTGACGAGTGGTGTATGTCTGTAGAGACGCGTTTAGATAGTTTAGTGCGAGAAAAAAGTGAAGTGAAAGCCTACGTCGGAGGATGTCCCTCGGTAATCACGGATGCTGGAGCGTATGACGCGCTCTTCGACATGAGACGCCGCTGGTCTAATAACGGTGGCTTCCCGCTGCGAATGCTTGAAGAGAGCTCTTCAGAAGTGACATCGTCTTCGGCACTGGGTTTGCCACCGGCCATGGTTATGTCGCCGGAATCCTTGGCGTCGCCCGAGTATGGAGCCCTCGAGCTATGGAGCTACGATGACGGAATCACTTATAATACAGCCCAGTCTCTGCTGGGTGCATGCAATATGCAACAGCAACAGCTACAACCTCAGCAACCACATCCAGCACCACCGACGCTCCCCACGATGCCTTTACCAATGCCTCCCACAACACCGAAATCAGAAAATGAATCGATGTCATCAGGTTAG

**>Bm_nscaf2855_219**

ATGACAAAGGGTAGACCAGTCTCTAAAAATTCAATATACCAGCCGGGTCGAGAGGAACTTTCGCCGGCTTCAAGCATAAATGGCTGCAGTGCTGATGCTGACGCCAGACGGCAGAAGAAAGGTCCTGCACCTCGACAGCAAGAGGAGCTATGTCTTGTCTGCGGCGACAGAGCCTCCGGATACCACTACAACGCACTGACGTGTGAAGGATGCAAAGGATTCTTCAGGCGGAGTGTCACCAAAAACGCAGTATATATTTGTAAATTTGGACATGCCTGTGAAATGGATATGTACATGAGGAGGAAATGTCAAGAGTGTCGATTAAAGAAATGTCTAGCGGTAGGAATGAGGCCTGAATGTGTCATACAGGAGCCCAGTAAAAATAAAGACAGGCAAAGACAAAAGAAAGACAAAGGAATATTATTACCTGTTAGTACGACCACAGTCGAAGACCACATGCCCCCGATCATGCAATGTGATCCACCTCCGCCCGAGGCCGCCAGGATTCACGAAGTCGTCCCGAGGTATCTTTCGGAGAAGCTGATGGAGCAGAACAGGCAGAAGAACATACCACCATTGTCGGCGAATCAGAAGTCTCTGATCGCGAGGCTCGTGTGGTACCAGGAGGGATATGAGCAGCCCTCCGACGAGGATCTCAAAAGAGTAACGCAGACTTGGCAGTCGGATGAAGAGGACGAGGAATCCGATCTACCCTTCCGCCAGATCACGGAGATGACGATCTTAACGGTCCAGTTGATCGTCGAGTTCGCCAAGGGTCTACCGGGCTTTTCGAAGATATCACAGTCTGATCAAATCACCTTATTAAAAGCCTCGTCCAGCGAGGTGATGATGCTGCGGGTGGCGAGGCGATACGACGCCGCGTCCGACAGCGTGCTGTTCGCCAACAACAAGGCGTACACGCGCGACAACTACCGCAAGGCGGGCATGGCCTACGTCATCGAAGACCTCCTACACTTCTGCCGGTGCATGTTCGCGATGGGCATGGACAATGTGCACTTTGCACTGCTCACGGCCATCGTTATATTCTCAGATCGGCCCGGGCTCGAGCAGCCGTCGCTGGTAGAAGAGATCCAGAGATACTACCTGAACACGTTGCGAATTTACATCATCAACCAGAACAGCGCGTCGTCGCGCTGCGCCGTGATCTACGGCAGGATCCTGAGCGTGCTGACCGAGCTACGCACGCTCGGCACGCAAAACTCCAACATGTGCATCTCGCTGAAGCTGAAGAACAGGAAGCTGCCGCCGTTCCTCGAGGAGATCTGGGACGTGGCGGAGGTGGCCACGACGCATCCCACGGTGCTGCCGCCCACCAACCCGGTGGTGCTATAG

**>Bm_nscaf2888_430**

ATGCCATTTATTGAAGACGAGTGGTGGTCCGCCGAGAATGAGGGCAGAATGGTCGATCTCTCAAATTGCCTTCAGGGACAGTTTCAGGACGCGGTAGTGGCTGCCGGCGGTCAGGCGGCCGCCCAGCTTCAGCAGATGGCCTCGTCACTAGGCGAGCTGTCGCAGGCCGAGCTGTCCAACATCGTGGGCGGCCTCACCCTGGAGCCGGAGAGCTCTGAAGCGGCAGACCCCGACGACATCCTGAAGCAGCTCGGGGAGACCGCGTTCGACAACTTCGATACGTTTTTCACGGATCTCACTAACGCCACCGCTTCGGGCGCACCCCCTATAGAGATAAAGCAAGAAGAGAACAACAACATATCGTCGCCAGCGTCCAGCAGTCAACTGCAAGGGTACTACCCTCAAAGTCAATTACATTTACAAAACAACGGGCAGCAAAGATTGCAGCAACTCCTTAGATCGGGCACCAACGCCATTAACAATGCCGTTAATAATAACATTAACAATGGAAGATATAATATTGCGTCGCAAAATCCGTTGCTCGCCGAGAAATTATCTTCAACGCCCAGCGGCATCAAGCAAGAACCAGTCAGTTCAGAGTACACGGGGACGAGTATGAACTATGGAAGTGCGTCGCCGTTGCAGCGAGTGCCGAGCGGGAAACCGCAAGCTCACGACGCTGGTGGAGGTAAAGGTTCGTTTTATTAA

**>Bm_nscaf2888_433**

ATGGCCGTGGGAGCGTTGAGCGATCGTGACGCATATGGATATGGTTACGGCGGCGGGGGAGGCGGGACTCACCACTTCGCAGCGCCTGCGCCCCCGCCGCTGCCGCACGACGAGCTGCCATACTCCGTATTCGACTTCGGCGACTACCAGCGCCACCATCATCACAACAAACTGAAGCCGAAGAAAAGGCCTCGCTCCGATGCACCTCCCACGCCGGGCGTCAAGCGCAAGAGCCGGGAGGGCTCCACCACGTACCTGTGGGAGTTCCTGCTGAAGCTGCTGCAGGACCGCGAGTACTGCCCGCGCTTCATCAAGTGGACGAACCGCGAGAAGGGCGTGTTCAAGCTGGTCGACTCGAAGTCGGTGTCGCGACTCTGGGGCCTGCACAAGAACAAGCCAGACATGAACTACGAGACCATGGGCCGCGCCCTGCGCTATTACTACCAGCGCGGGATCCTCGCTAAGGTGGACGGCCAGCGCCTCGTCTACCAGTTCGTGGACGTGCCCAAGGACATCGTCGAGATCGACTGTTCGTTGGCGTAG

**>Bm_nscaf2859_001**

ATGTCGTGTGGCGCTGAGTTGCGCGAGCGGCACTCGGTGCTAGTGAGCATGCTGGAGGCTCGCCGCGAGTCCAGTGACTCGGGCTGCTCGAGCGACGACAGCTCCGACGTCGAAAGAGACTCTACTAAATGTAGCTGTGACCCGCAGGGCTTCTTCCGGCGATCTATACAACAGAAGATACAATACCGGCCCTGCACGAAGAACCAGCAGTGTAGTATCCTCAGGATTAACAGGAATCGGTGCCAGTATTGCCGACTGAAAAAATGCATCGCCGTCGGAATGAGCAGAGATGTTGCGAATAAAAGGGGAAGTACAATGATCGACGAGAAACAATGGGATCTCGCATCTGGTGCGGCCCACATTGAGCAGCGAAAGTTAAGAGAAATGGCGAGAGGGCGCTCGCTCTCTGCTTTCGTTTTCAACCCTGTGCGATTCGGTCGTGTACCCAAACGCGAGAAAGCGCGTATCCTCGCAGCGATGCAACAGTCGTCGTCGTCTCGTGCACACGAGCAAGCAGCTGCCGCTGAACTTGATGACGCTCCTCGGTTGCTGGCGCGAGTGGTGCGCGCTCATCTCGACACGTGCGAGTTCACGCGTGATCGCGTCGCTTCCATGCGAGCCAGAGCTCGCGACTGTCCCACCTACTCGCAGCCTACTCTGGCTTGCCCACTAAACCCGGCGCCAGAGCTGCAATCTGAAAAGGAATTTTCGCAACGTTTCGCCCATGTGATACGTGGCGTGATTGACTTTGCCGGCCTCATCCCTGGCTTCCAGCTGCTGACCCAAGATGACAAATTCACGCTGCTCAAAAGTGGTCTGTTCGATGCATTGTTCGTGCGACTCATCTGTATGTTTGACGCTCCTCTTAATAGTATCATCTGTCTCAATGGGCAACTGATGAAGAGAGACTCCATCCAGAGCGGTGCCAATGCAAGGTTTCTCGTTGATTCTACTTTCAAGTTTGCGGAACGTATGAATTCCATGAATTTGACGGACGCGGAAATAGGACTTTTCTGTGCTATAGTCCTCATCACTCCGGATCGGCCTGGCCTGCGAAACATAGAGCTAGTGGAAAGAATGCACTCGCGACTGAAGGCGTGCTTGCAAACTGTCATTGCACAGAACAGGCCAGAGAGACCTGGGTTTTTAAGAGAATTAATGGATACATTACCTGATTTACGCACTTTAAGCACGCTTCACACAGAAAAACTTGTTGTTTTCCGAACGGAACATAAGGAGTTATTGCGTCAACAAATGTGGAATGAAGAAGAAGGTGTTTCCTGGGCCGATTCCGTAGTGGAGGAATCAGCTCGTAGCCCCATCGGGTCTGTATCCAGCAGCGAGTCCGGGGAGGTGCCGAGTGACTGTGGCACTCCTTTACTGGCAGCAACGTTGGCCGGTCGCCGGCGACTTGACTCTCGGGGCTCTGTCGATGAAGAAGCTCTCGGCGTCGCACATCTGGCTCACAACGGACTCACCGTGACGCCCGTCCGCCCTCCTCCCCGGTATCGGAAGTTGGATTCCCCGACTGATTCGGGTATTGAATCTGGCAACGAGAAACACGAGAGGATAATCGGACCCGGGTCGGGTTGTTCTAGTCCGCGGTCGTCCTTAGAAGAGCACACCGAGGACAGACGGCCCACCGCGCCCGCCGATGACATGCCCGTGCTCAAACGTGTGCTTCAGGCTCCACCTTTGTACGGCGGAACTTCTACATTGATGGATGAAACCTACAAACCACACAAGAAATTCCGCGCTATGAGGCGCGACACCGGAGAAGCAGAGGCTCGTCCAGTGCAGCCGACGCCGTCGCCACAGCCGCTGCACCCGCACCCGGCCAGTCCGGCTCATCCGGCTCATTCGCCGCGACCACCGCGCATTTCTCTGTCATCCACGCATTCGGTGCTCGCTAAAAGTTTAATGGAGGGGCCGAGAATGACTCCTGAACAATTAAAACGTACCGATATGATCCAGCAGTACATGCGGCGAAATGAAGCTGGTTCTAGTGTGGAAGGATGTCCGCTGCGAACTGGAGGGCTGCTGACCTGCTACCGCGGTGCGTCTCCGGCTCCGCCGCCAGTGCTGGCGCTGCAGGTGGACGTGACGGACGCGCCGCTGAACCTCTCCAAGAAGTCGCCGTCGCCGCCTCGTTCGTACATGCCGCAGATGTTAGAGGCGTGA

**>Bm_nscaf2859_003**

ATGCAGTGTTATCCGAAGTTGTCACCTAAGCGTGAGCCTCCGGAAGGATTATACGAGATCGAGATGTTACCCGGGGCGCGAAGATTAGAACTGCCGGCTCCTCCGGGAAAGGAGTTCCGCGCGCCCGTGCTATTGGCAGGGCCCTCTCTTGCTCCGACGCACTCCGTCATTCAATGCATGCGACCACCACCGCCGCCACCGCCACCACCTCCACCACGTCTCCTCAAGCCTCCATCATTCGAAGAACCGTCGAGCTCCATCCCCGATTTAGGCAAACCACGTCATTTATTTTGGGCAAGTCCATAA

**>Bm_nscaf2964_066**

ATGGAGGGTAGATCCCAAATCGAGATAATACCGTGCAAGGTATGCGGAGATAAATCGTCGGGGGTGCACTATGGCGTGATCACCTGCGAGGGATGCAAAGGATTCTTCAGACGATCCCAGAGCACAGTGGTGAACTACCAGTGTCCTCGCAACAAGGCCTGCGTCGTGGACAGGGTCAACCGCAACCGATGCCAGTACTGCAGACTACAGAAGTGCCTCAAACTCGGCATGAGTCGTGATGCCGTCAAATTCGGTCGCATGTCGAAGAAGCAGCGGGAGAAGGTCGAGGACGAGGTCAGATACCACAAGGCGCAGATGCGGGTGCAGGCTGATGCGGCGCCGGACTCCGTCTACCCAGGATACGGGTCGCCGTTGTCTTCGTATGGCTACAACAACGCCGGGCCAGCGCTACCCTCGAACATGAGCGGGATGCAGCCGCAGCCCCCAGCTCAGCCCCCGTACGAAGTCTCAGGCGACTACGTGGACTCCACAACGACATACGAGCCCAAACAGACAGGGTTCTTGGACGCAGACTTCATAAGTCACGAGGAGCGCCAGAAATCGACCATCGTCCGACCGTCGACCGCGACCACGACCGCCACCACGACCACGATCCGACCATCGGCCATAAACGAGCTGCCCAGATCACGGCTGCAGGAGTACGACCGGTACGACGACCGGATTCAGTCGCCGTCAGGGGTCATCAGCATTAAGCAGGAGATCAAGCCTGAGACTTCAATGGGCGTCGATAACTTGGTGGCGAGCTACGTCGACTCGACAACGTTCCTACACAGTCCTTCTAACATGCAAAACAGCCCGATGGACATACAGAACACGGTGCTCGTCAGCGGCCAGAGCTCCGTCTCATTGACCAGCGAGGACTTGAGTCCTGATGATTTGACTTCGAGCAGCGGCCACGAGAGGCTAATGGACCCCATGAACATGAACATGTCCGCCATGGGAATGGTGAACCCCAATATTGTGTCAACGAGAAGACACCACGGCGCTAATAATTCTAATGACGATATGCCTTTGGAGGGTGACATTAGCAAGGTGCTAGTGAAAAGTTTGACAGAGGCGCACGCGAATACAAATCCGAAGTTGGATTACATACATGAGATGTTCGGCAAGCCCCAGGATGTTTCTAAGCTCTTGTTCTATAACTCCATGACCTACGAGGAGATGTGGTTGGACTGCGCCGACAAGCTCACCGCGATGATCCAGAACATCATTGAGTTCGCGAAACTCATACCTGGTTTCATGAAGCTCACCCAGGACGATCAAATACTGCTGCTTAAATCAGGTTCGTTCGAGTTGGCGATCGTCCGTTTGTCGCGGCTAATCGACGTGAACCGCGACCAGGTGCTCTACGGAGACGTGGTGCTACCCGTGCGGGAATGCGTGCACGCGCGCGATCCCAGAGACGTAGCTCTGGTGCAAGGAATCTTTGAGGCTGCCAAGAGCATCGCTCGACTGAAGCTGACCGAGACTGAACTGGCTCTATACCAGAGCCTTGTGCTCCTGTGGCCAGAGCGCCACGGCGTGATGGGCAACTCGGAGATCAGGTGTCTCTTCAATATGTCCATGTCGGCGATGCGGCATGAGATCGAGGTCAACCACGCGCCGCTCAAGGGTGACGTCACCGTGCTGGATACACTCCTGGCCAAGATACCCACTTTCAGAGATCTCTCCCTGATGCACCTCGGAGCGCTGAGCCGTTTCAAAGCGACGCATCCGCATCACGTTTTCCCAGCTTTATACAAAGAATTGTTCTCTTTAGACAGTGTTTTAGATTACACGCACGGATAA

**>Bm_nscaf2970_078**

ATGGTGGACAGTCAGACGCAACACTTCTGTCTCCGATGGAACAACTACCAGCGCAGTATTACCAGCGCCTTCGAGAACCTTCGCGACGACGAGGACTTCGTCGACGTCACGCTCGCCTGCGACGGGAAAAGCCTCAAAGCTCATAGAGTCGTGCTGTCAGCTTGCAGCCCTTATTTCAGAGAATTATTGAAGTCCACGCCGTGCAAGCATCCAGTGATCGTGCTTCAAGACGTGGCGTACACAGACCTTCACGCCCTGGTCGAGTTCATCTACCACGGCGAGGTCAACGTTCACCAGCACAGCTTGTCCTCATTCCTGAAGACAGCTGAGGTGCTCCGTGTCTCCGGTCTCACACACAATGACGCTGCCCAGGGGCCCCTGCTGCAGACCATGCGGTCGTCAGCAGCTCCGTCACCCCACACGCCACCCCACCCGGCCCACTCTACCCATATACCGCAACCCTACCCTGACAAACTCGAGGAAGCTCTTCTTCAACCCTCATCCAGCATCCCGCCCATGATGCGCCGCATTCCTCTTCCTCCACCGCGACGCATGAGCTCCACTGACAACTCGCCTGACGTCATAAAGCGTCCACGTCACGACAACAACAACACAGAATCCCCACAAATGCACGCCGCCGACTTCTCGACCAAGAACCACTCGATTCTGAACAGCAGGGGTCACGAGCAGGGGAACAACGGAAACGGCATCTCCAATAGCAGCTCGTCACCTTCCCCGAGGCTGATGGACGAAGTGAAAAACGAACCGGTCGACATGATATGCCCTTCCAACCCTGATATAGATCGCTGCACGGATGACACACCGCCGCATACTCATCACCGACCAATCAAAAACAAACGGTTGTAG

**>Bm_nscaf2970_079**

ATGGCGAGCACATCTCAAGTGGGTCCCCTGGGCGCGGGCCACCGGTGCGAGGTGTGCGGCAAGCTGCTGTCCACGCGGCTGACCCTCAAGCGCCACACGGAGCAGCAACACCTCCAGCCGCTGCACTCGGCGCGCTGCACGCTCTGCCACAAGGTCTTCCGCACGCTCAACTCGCTCAACAACCACAAGAGCATCTACCACCGTCGCCAGCGCAACCCGCCGCCCCTCTCCCAGCCGCAGAACCTCTCCACTGCGCCGGAGCCCAAACTCAACCCTCCCCACCACAACATCGACTTCTACAAATTCAAAGACCAATTCAATGTCTAA

**>Bm_nscaf1690_227**

ATGACGATGGACCAGCAAACAAGCCTCATGTCCCTTAATATGTCCCCATTTGATTTAAGTCCTGGTCCAGAAGGATCGGCTTCGGGTGGTGGACCTTCCAGTGCTTCCCAACAATATGTGCCTCAAGGCGCAGCATACCAATGCCCCCCTGAACAACAATCATTTGGATATGCCAATCTGGATGCTTCATATCTATTTCCGACAGGCACTGGAGGTGAACCAGGAGCGTATCTGCCGACAGCAGGAACAGTTTGCGACCAAACTGATACCAAGGATGTAATAGAAGAATTGTGTCCCGTCTGTGGAGACAAAGTCAGCGGCTACCACTATGGATTGCTGACGTGCGAATCCTGCAAAGGTTTCTTCAAAAGAACCGTTCAGAACAAGAAGGTTTATACATGCGTCGCCGAAAGAGCCTGCCACATAGACAAAACTCAACGGAAACGCTGTCCCTTTTGCCGTTTCCAGAAATGTCTTGATGTGGGCATGAAACTTGAAGGCCAAACGTAA

**>Bm_nscaf1690_228**

ATGTACAAACGAGACCGTGCCCGTAAACTACAAATGATGCGTCAGCGACAAATCGCCGTTCAGACTCTGCGCGGTTCTCTAGGGGATGGTGGATTAGTCCTTGGTTTTGGTTCTCCGTACACAGCTGTATCCGTTAAACAAGAGATACAGATTCCGCAAGTATCATCATTGACGTCCTCGCCTGAGTCGTCGCCGGGACCAGCGCTCCTTGGGGCTCAGCCACAGCCGCCGCAGCCACCTCCACCACCAACTCACGACAAGTGGGAAGCCCACTCACCACACTCGGCGTCGCCGGATGCTTTCACGTTCGATACACAATCGAACACCGCCGCTACACCATCCAGCACAGCCGAAGCTACTAGCACTGAAACTTTACGAGTTTCTCCAATGATCAGAGAATTCGTACAAACCGTCGATGACCGCGAGTGGCAGAATGCACTGTTCGGACTCTTACAAAGCCAAACATATAACCAGTGCGAAGTAGATCTCTTCGAGTTAATGTGCAAAGTGCTGGACCAAAATTTATTCTCTCAAGTGGATTGGGCAAGAAACACAGTGTTCTTTAAGTATTTAAAGGTTGATGACCAAATGAAACTTCTACAGGACTCATGGTCTGTTATGCTGGTTTTGGATCATTTACACCAGAGAATGCACAATGGTCTGCCAGACGAGACCACACTCCACAACGGGCAGAAGTTTGACCTGCTGTGTTTGGGGCTACTTGGAGTTCCTTCATTAGCCGACCACTTCAATGAATTACAGAATAAACTAGCAGAATTGAAATTCGACGTTCCAGATTACATATGCGTTAAATTCATGCTTCTTCTCAATCCCGAGGTTAGGGGTATCGTAAACGTGAAGTGCGTTCGTGAAGGTTACCAAACAGTACAAGCCGCCCTTCTTGACTACACTCTTACCTGCTATCCAACGATACAGGATAAGTTTGGAAAACTTGTAATGGTAGTGCCAGAGATACACGCTTTAGCGGCTCGGGGAGAAGAGCACCTGTACCAGCGGCATTGTGCAGGCCAGGCACCTACCCAGACTCTTCTCATGGAAATGCTGCACGCAAAACGCAAGTGA
