# Supplementary material for: Transcriptomic analysis of developmental features of Bombyx mori wing disc during metamorphosis
Source: BMC Genomics. 2014 Sep 27;15(1):820. doi: 10.1186/1471-2164-15-820 (PMC4196006; doi:10.1186/1471-2164-15-820)
Supplement: Supplementary file 4 — Additional file 4: Assembled nucleotide sequences of transcripts in Table 2. (DOC 32 KB) [file 12864_2014_6525_MOESM4_ESM.doc]

**>Bm_nscaf2828_039**

ATGAACTCGTCTATTGGGACAGGATCGAATTACGAGAAATGCTCTAAGATGGCAACTCGGGAGCCGGCAGAGTTTCTGCCCGTGGCAGTGACAACAAATTGCGTTTACTATGATTCACAAGCTGCAAGCTGCGGAGACTCTTCTTCAGATTCACGTTCGCCAGAGACTACTTCAGCAAAGGCTAGCGATCGTGCTTCTCGAATCATTGCAGAAAAAACTCGTCGCAGCCAGTACAATGCATTAATCCATCAAATGAAGTCTCTCCTATCGGACATTGCGCATTCCCAACGGAAAGTTGACAAAACTAGTATTTTGAGACACGCCGTCAACAAACTGCGTAACGAACACGTCTTTGGCGATACCATTAAATGCTGCCATCTAGAAACATGGTCCCCTGCATTTTTAAAGTTTTTTGATCTCATAGGAGGTATCATGTTTGCTGTCACTTGTCGGGGTCGCATTTTTATAGTTTCACCAAATATCCAAGAAAAATTAGGTTATTGCCATATAGATTTAGTAGGCCAGGATTTTTACAAATATGTTCATGAGGAAGACAGAGAAACCCTACGTTGTCACATTTACCCACCTGAACTTCAAACAGGCTGTGATGATCAACTTTTTGAACATAACCATAACTTTCATATTCGTCTGATGAGAGCAGGAGCAAGGTCTGATCATCCCAGATATGAAAAATGTCGTATTAGTGGTATGTTAAGAAAGTCTGATAGAGCTACTGGGAATGAAGTTCAAGATGAGCATGTAGTTAGAAGGCAAAGAGTACGGAATAATCGCACATTTTCCTCCAGTGGAAATGACTTTGTTTTTATTGGTATGATACGTGTGCTTTCTAGCAACTTGCCAGCTCGAATCTTGCCACCAACAGCTTATTCTGAATATTGGACGAGACATTTGATAGATGGCCGGATAGTACAATGTGATCAAAGCATATCATTGGCAGCTGGTTACATGACTGATGAAGTAACAGGTACTTCAGCTTTTGTTTTTATGCACAAAGAAGATGTGCGTTGGGTTATCTGTGTACTTCGTCAAATGAATGATCAGAGTCGAGAGTTTGGGGAATCATATTACAGGTTAATATCTCGTTCTGGTCACTTCATATATATGAGAACTAAAGGATATCTTGAAATTGATAAAAAAACTAAAAAAGTTCAAAGTTTTGTCTGTGTTAATACTGTTATTGGTGAGGAGTTTGGAAAAAGAATGATGGAAGAAATGAAACGAAAGTACTCAGTTATCGTAGGAATGGATAAACAACAACAAGAAAGAGTGTTGACTTATGATGATGCTCCGGTTGAACATCCAAAGTGTCTTGAGCGGATTGTAATGCATCTTGTGGACCTTCCGTAA

**>Bm_nscaf1690_114**

ATGGCTGATTGGTCTCTGCTGCACCGCGAGTGGGCCGACTATTACAACCCCTATCTATACCAAGGGTACCCGCCGTACCAGCAACAAGGGGTGGCGGTTCCACAGAATGCGCCCCCGCCGCAAGCGCAACCGTATAATATGCGGCAGAATTCGCCGGCTCTGGCATTGCTGCTGAATACGCAGCGTAACCAGATGCCTCTAGATCCTATTATCTGTCCCCAACCCAGCGGAAGTGATGAGCAGCCACCCAAAGTGTAA

**>Bm_nscaf1690_115**

ATGGTGCATACGAATCCCGATTTCAACCCGGAGTTCACCGACGCCGTGCTGAAATTATTCAACGGATTTTTGATTACTACTACATATAGAGGCATAATTGTCGTGGTGTCAAAAAATGTACACCAATACTTGGGTTTCCCCGAGCTTGACCTCCTGGGGCAGAACCTGGTGAATCTGACTCATCCGAGAGATCGCCAAATGCTGCTTGAGAAGCTGAAGCCAAGAAGCCAAGTTTTAGGACCAAACGGAGAGCTGTTGATTCCAAATGAGCCCGATGGTGTTTACAAAGTTGTTGAGGGGTTGCGTCGAGAAAAAAGAAGCTTCACCATCAGGTTGAAGAAGCAGGGACCACGTTCCGAACCAACGCAGTATGTAATGTGCCACATCGAAGGCTCATTCAGGAAGGCGGACGGTGCCAACCACACACTTAGCCGCTGTTGTCAGGTGGTGCGTCGCTCTCGTACACGTGGCGAAGCTCCTGAATGCAGCGGAAACGATATAGTGTTCATTGGCGTCGTGCGTCCGTCAGTCGAAACCTTCCATTCAGAGAGCCGAATGGAATCGTTTTGTATGGAGTACCGCACTCGCCACTCTGTCGATGGACAGATCGTCCAATGCGAACAGCGTATCTCGTTGGTCACCGGCTATATGACCCACGAAGTCAAGGGCGTTAATGCCATGAATTTTATGCATCGCGATGACGTGCGCTGGGTCGCCACAGCTCTACGAGATATGTATGATCAACATCGATTGTTCGGAGAGTCCTGTTACCGTCTGATCACCAAAAATGGTCAATTCATTTACATGAGGACACGTGGCCATCTTGATATTGAAAAAGACTCTAAGGCTGTAACCACTTTTGTTTGCACAAACACCGTTATTGGAGAAGAGGAGGGAAAGCGTTTGATCAAAATGATGAAAAAAAGGATCGCCTTATTGACAAAAACAAATGACAAACTCCTCAAATATGATGAGGGTACTTCAAATCAATTGGTTCCAGTCGAAGATCCCAAGCAGCTTGTGAACGTCGTGTTACATATGGTCACAGATTTGCCAACATCAAAGCCAGGCATAGCTTTGAAACAAAATAATCCTGCTTCTCCATCTCATAACTTGAGCATAATTCCACCAAAGAAAGAGCGTATTGTAAGTGGGGTGGAAAAAATTTACACCATTTTCAAAAACATGATGGGTAATACCCCTCCAGTACAGCAGACGCCAACTTGGACAGTGGATGAACCACAAGATGCTATTTTGGATATTAATATGATAAACCAGCCATTATTTGCGACTGAGAATTCTTCTCGTATCCAAGAAATTGATGAATCGAACACGTTTGAAATTTTCGATATGCCGTCGACATCGACCGCTTTGTGCCAAGTGGAACCAAACTACTTTGAAGAAGGGCAGCTAAATGTCACATCTAACAATTTAATGTTTTCCGAAGCAGTGGCCGTCGAACAATATAATCCCGAATTTGGATTGACGGCTACTTCACCTGACGTAACATATCATGATTACTTAAATGTACAGGAAAATGAGATTACATTGGATGACTTCATATTTCCTGAATTAATTGACGAGCCACAGGGAATACAGAGTCCTACTCAAATTAAATACCATTTGGTAATCGATTCTGAGCAAGATTTAAACGAAGCTTTCCAACAAGCCAACAAAAATTCTGCTGCGAACCTGGAATCTGACCTCAACAAAATTGGCATGAAGCGTCCAAATAACTTTTCTGAAGTAGCTTCAAGTAACAAGAAAATATCTAACCCCAATATCGTTGCTGAGAACGACTTCTCCAGTGAATTTGCGTGTCTCGAAAGTTTCTTGGACGATGTCACTCTGAACACTCAGATTGAAACGGCCATTAAATCCTTGGAGCAAACCATCGATCCTAGTTTCCCGGAATTGTTGATTTCATCCGAGGTTCAAGAAATTCTTGGCAAAATAGAAGAGGAACAGAAAAACCAACAACAATAA

**>Bm_nscaf3078_04**

ATGCTGCCCGTGGTTCAATCCGAGCCCATGCTTTTCAACTGCGGTGTTCACTCTGTGAATGGCGCCGCAACTCCTTCGATATCGTTGCCACCGTCGTTAACACCAGAATCGGACATTGGCGAATTAGTTGACATCTTCTTCGATGTTGATGTTGCTGTTAATGACCATTCAAGTGTCGATGCCGGTATTCACGTTGCGAGACCTGGTGGACTATGA

**>Bm_nscaf3078_05**

ATGTGCATATCTCATAGTAATAAGTGCCAGAATGAGAAAGAGCGACGGAAACTTGAAAACGAAACTATAAATCAACTTGAGGAGCTTCTTGGTACTTGTCTCGCCGAAGTAAAGCAACCGGACAAGAACGGTATTGTCCGAGAGGCCACGCGTCAGATCCAGGAGGTATTGAAACGGCGACGCGAGTGTCCCAGCGAGTGTCCCCTACGATCGCCCCAGTGTCTATCACCAGTACAGGCCGGGGAGATCAGCTCAACACAGCCGCAGTTGCCCTGCACCGGATTACATTATTCTGAAGTCACAACTTTAATTGAGGCACTCAAACATTACACCAATAACCTCGGATGGGTTTTATTGGAAATTAATTCGAAAGGTGAAATAGAGTGTGTATCAGATAATATCAAGGAGTTTATTCTTCACGATAGAACAGAACTATATAGGAAATCGATATTTTCGATTTTACATGAGAAAGATCATGCGAAACTAAGACCGTTACTTAGGAACATACAATCTTTCAACTGGGACTCGGCAGATATTGATAAATTCCACTTTGTGAAAGCAAGGCTCCTCGTTAAAAATTCAAATGGGACTGACTGTGGCGTTTACGTGGAGACAGTGATACACGCTGCGCCAGTGCGCGGCTCTTCTTCGGAGGAGGCCGGCTCCGTCATGTGCGTCATCCGGCGCTGCGACGACGCGTCGGCCGTTCTTATTCCCGACGACGGCGGCCCGCCCGCCATCACCGCCAAGCAGTCCGATCATATCGTCTTCAGATTAGACTGCAATTTTAATATTTTGTCTTGTGACTTGAGTGCAGTGGACAGTATAGTAAACTCACCAGTTTCTCTTGTGGGCACTCGATATTTGGACTTGGTTGACAGTGTTGATCGTCTACGTGTTGCTGCACATCTGCTGGAAGCAGCATCAGCGCCTGCGCCGCCTGCAGTCAGTGAACCGTTTCGACTGCGCGTCACCCCCGACCATCCGTGGTTGCGTGTCAGCGCCCGATCGCGGCTGTTCAGGTCTCAGGCCACTTCTGGTGAACCTGACTTCATAATGTCAACGCACAGCGTTCTTTGTGATGAGGAGATAGATATGTTGGAATCTGAAAGCCCTCGCCCTGCGGTCGGCGGCCCGCTCATGCCCTCCGTGACCAACGGAGAGTCTTCCATGTGCGAGTCGCGGTATCGATCTCCCGTCAGTCCGGCCACGAATCCGTTTTCTATAAACGATTTCGAGTTTGAACCTTGGGCTTCTTCTCTCCTTGGTGAAATGTCAAATGAAGATTCCAAGGAACCCAAGGATGGGTCCGTGGAGGGACCGCCTTCAACCCCGCTCACGCCTCGGGCGCCTTCCACTCCTGGTGAAAGCGCTCACGTCGTACAGCCTCCTGAAGAGCCAAATCGGTTACGAACGCTGTTGAGCAAGAAGCCTAATTCGTCTTCCGAAGCAAACTCCAACTCGAACAATCGTATTCTGAAAGATTTATTGAAGCAAGAAGACGAAGAGGCGACAGGAAGCGAGACGTCGGCGCCGCACACCCCGCACACGCCGATGACACCGCACACCCCGCACACGCCCGGCGCGGCGCTCTCCCCGCTGCACTCCGCGCCGTCGCACGCGCGTCCGCAGGCGCACTTGCAGCCGCACGCCTCGCACTCCGCGGGACAGCACTCCATGCAACAGCTTCATCACAATAATTCCGACGTCCTCCTTAAGATATTAAACGATAAATCAGACGAAGATACGGAGGAAGGGAGAAGAAGCGCTTCGGACAGTAATCGCAGTATGTCCCAGCCCAGCGCGCTCCTCTCCCAGCTGTTGTCGAGCAGCAACGGCCCGGCGGGTAACGGACGCTCGCAGGACGGAAGCGACAACTACCTGGATAGGATCGCCGGTGTCAAACGCAAATTCGAAGACGCCAAAGCGATGAGCGGTAATATGAAGAGAGCGACCCCAGAGAATCAACAGGTGACGTCCAGTGCTGTATCGGTAGCGTCATCGACGGCGACGTCTACGGCCGGGAGCCCGGCGACGAGCGGCCCGGGCATGAGTCCGCTGTGCAAAAAGAACCAGATCCTGGTGTCGTTATTGGCGCGCCAGCAGCCCACGCCCACCACCCCGCTGCCGCTCCCCAACCCCAGCCTGCGCCCCTACGGACCCACCAACCGACCGCGAGCGCCGCCTCCGCCTCACCCGGCTCAGCATCATCACCACCACGCCATGCAACACCACCAGCGGCACCATTCGACGCTCTCCAACATACTCACCGGAGTCAACCATCGGGCGAGCAACGTGAACGGCGGCGGCGGCGGCGGCCCGACGGCGGAGTGCTCGTCTGCGCAGAGCCACCTGCAGATGGTGCTGCAGGGCGGCGCGCGCTACCCGCCCGCCTCCGCGCCCGCGCCCCTGCACTACACCAACACCACGCCGCACCACACCTACAACAGCCAGCCGCCCTCAAGTAGCAGTAGCCTGACTCAAGCCGTTCCCGGTGACGGCGAGGTCCCCAGTGATCTGATGCTGTCCGACATATTGGACGAGTTCATAGAGAGCATGCCCGACTCGGATCGTTCCGCTTCTGATGTTAGCATGCGCCAAAGACAGGGCATGAAAGAGAAGACTGCGATAGTGAACGCCATCCGACAGAGACTGATGCACGAGTGTGAGACGGTCACGAAGAATACAAATGTCACGAGTCCCAGTGCACTGCCGCCATTCTCTGTGCAGAGTCCGGTGTGCAGCATGTACCCGGGCGGCGCCAGCCCAGCGGCCGGCGGCTCGCGCCCGCACTCCATCGCCGAGCAGCGCGCGCGCCTGCTACAGATGCAGCGCTCGCAGCAGATGCTCGTCTCGCCAGAGGCCGCCGATCAGCCCCAGCAGGATCTTGGTTCGACCATTAATGCTCTCGTTTCCGCGACTCCGCCCAACGTGGCTCTGACTCGAACGGATTATCATCATAATCTATATCAAACGAGTCAAATAGGGTCAAATTATGGTACAAATAAAATGACTACCACTAATCAGAACCCCATGTTAAGCAGACAGCTTAGTAGCGAGCTGACTTGTCTCGGCACGGTCAGCGATAGTTATGTCTTGCCCGTCGATCGAATTGCGAAGCAGAGAAGCCGTTTCCCATTTAGCAATCAAGTGCGGCGGCTGAGGTGCTGTGTGCCCACAGGTGCGGGAGGCGGCGGCGGGACGAGCGGCGGGGCGGGCGGCGGCGCGGGGGGAGGCGCGGGCGGCACGTCGGAGTACGTGCGCAACGAGCTGCGCGCAGTCGTGGGGGCGCGCTCGGCCCGCCCAGACTTGCATACGCTGCAGCCGCCGGACCTCGACCCGCTCGTCTCCTTCGACATGCCCGCGCCAGGTGGCGGCAGTACCGCACTGGGAGGCCGGGCCGCGGCGGCGACAAGCTCGTGGGAGTCGCAACAGTGCACCCCGAATACTACGGAGGCGGGCTCGGCGGAGGCGGCGGACGGTGATGAGACGCAGACGGGCGGGTCGGCGGGCGCGGGCAGTAAGGCGTCGCTGCTGCAGAAGCTGTTGTCGCAGTGA

**>Bm_nscaf2589_179**

ATGATAGGTGACGAGGAGCGAGTTCATCAGTGCGGCGAGTGTGGTTTGACATTGTCCACCCGCAGTGCGCTTACAGCACACGCACGATCCCATCGTTCTACTGCTGATGCACATCGCTGCGACGTGTGCCATAAGACTTTTGCTGTGCCTGCACGACTCGTGCGCCACTACCGAACCCATACTGGCGAGCGACCATTTGAATGTGAATATTGCCATAAAATGTTTAGTGTGAAAGAAAATTTGCAAGTACACCGTCGTATTCACACAAAGGAGAGGCCGTATCGGTGCAATGTTTGTGATGCGGCATTCGAGCATTCCGGAAAACTTCACAGACATGCTAGAATCCATACCGGCGAGAGACCGCACGCATGTCCACATTGTCATAAGACGTTCATACAATCTGGTCAACTGGTCATACATTTAAGGACCCACACAGGTGAGAAACCCTATCGTTGTCCTGCACCTGGATGCGGAAAGGGCTTCACCTGCTCTAAACAGCTTAAAGTACATTCTCGTACCCACACGGGAGAGCGACCCTATACTTGTGAAATTTGTCTCAGGGACTTTGGTTATAATCATGTTCTCAAATTACATCGTTTTCAACATTTTGGCGAGCGCTGTTATCGTTGCACTGTATGCGACGGTACATTTAATACAAAAAAACAAATGGAGGCCCATATTTACAAAGAACATGGCGCGGAAGCCCCCCGCACTGCACCTTTACAGAGTACAACACCAATGGCTGCCGATGGTAAAGTGATGTGTGACTTGGTTGAAGCTGCTTTGCAACAGCTTCCTCCTACTCCACCTAGTTCACCGCCATCACCTAGATGTCCCAGTATTTCAGTGGTTCCAATGATGCCATCGTCAACTCAAGTCAACGATACGCCGACATCATTAGCTGCGGTCCCCGTACATTACATTTCGTTGCCGTCTGATTTACCGCCTAGAAAACGTAAATTTATTCCTCACATAAACGAAGAAATTACTCCGGCTGTGCGTCATACTTCTGTTATACAATTTGCTCCGCCAGCTACAGAATCATAG
