# Supplementary material for: Transcriptomic analysis of developmental features of Bombyx mori wing disc during metamorphosis
Source: BMC Genomics. 2014 Sep 27;15(1):820. doi: 10.1186/1471-2164-15-820 (PMC4196006; doi:10.1186/1471-2164-15-820)
Supplement: Supplementary file 5 — Additional file 5: Assembled nucleotide sequences of transcripts in Table 3. (DOC 42 KB) [file 12864_2014_6525_MOESM5_ESM.doc]

**>Bm_nscaf2589_261**

ATGTTCGCCGAGGGGCCGAGCGCCATAGAGTCGCGCTCGCCAAGCGCCTTCACTCCTAACTCCTCCAGCATGCTACTGCTGCAAACACACAGCAACTACGGTTCGTCCTTCTCTGATCTGCTGTCACCACAATATCAAGAAGATTCCACGGAAATCTTAGAAGAAAACCTAGATCCATTCCCGGACGTAGAGTTTCACGCACCAATCGTTCCGGAATTTAAATCGCAACGGGACACCTCGTTCAGTGAAACATCATCACCGATACCTGGTCCTGCGTTGCCCAGTTTCGAGGAGACATATTCAGTTCGGTATCCGAAACACGAAATGGCAGAATTCGGTATCAAAATGGATGAAGATTGCTACAATGTGAGTGCATATTCCCATCAGGGACATACGTCTACTCAACTTCTTTACCAATATCATCAACCACCGATTCCTTATGTTCCGTCGACGTACTACGCGCCACCTCAGCCTTGTAGTCCTACGTTTGACACCGTAACTCCAAACCAAGATTCTTATTCTTTGCCACCTTTTCCAAGCACTGTTGATTTACATATATCAGCTGAGCAGGCTGCTAGACAACGGCGTTCGTCATTACCTGTGCAAAGATCTGAATCCAACAGCTCCAATGACAGTCCAAAATTGCACGGTACCAGAGTTCACTGTATGCAGCCTTCCACTCCGAATTCAGCATCAAGTTCTCCTGGAGTAGCACCTGCTGATAACACTGGACCACGTGCAGCACCATCTTCGCCCAGCCAGCTCTGTGCTGTTTGCGGAGATACAGCCGCTTGCCAACATTATGGTGTCAGAACTTGTGAAGGATGTAAAGGGTTCTTCAAGAGAACCGTTCAAAAAGGATCCAAGTATGTTTGCTTGGCTGAAAAATCTTGCCCTGTTGACAAAAGAAGACGGAATCGATGCCAATTTTGTCGGTTTCAAAAATGCCTCGCCGTGGGTATGGTGAAAGAAGTGGTTAGAACTGATTCTCTAAAAGGCAGGCGCGGTAGGCTGCCCTCAAAGCCTAAATGTCCACAAGAATCTCCACCAAGCCCACCCATCTCTCTGATAACAGCCCTTGTTCGAGCTCACGTTGATACTTCGCCGGACTTTGCTAATCTAGACTATTCTCAATATCGTGAGCCGAGTCCTCTTGAGCCACCGATGTCTGATCTTGAAGTTATTCATCAGTTTTACTCTCTTCTAACAACATCGATTGATATGATCAAACTTTTCGCTGAAAAAGTACCTGGATATGGTGATCTATGCCCCGAGGACCGAGAGCAACTATTTGCTTCTGCTCGTTTGGAACTGTTCGTTCTACGGTTGGCATATCGTACTCGACTTGAGGATACTAAGCTCACATTCTGCAATGGGCTAGTTCTCGATAAGCGACAGTGTCAGCGATCGTTCGGAGACTGGCTTCACGCAGTTCTGGATTTCAGTAATACGCTTCACTCTATGGATATCGACATTTCTACATTTGCCTGTCTTTGCGCACTGACTTTGATTACAGATAGACACGGCTTAAAAGAGCCGCATCGTGTGGAGCAGCTTCAAATGAAAATTATCGGATGCTTGCGAGCGCACATGCCGGGCGGAGGTGGATCGAGCAGCGGCGCGCCCCACTTTAGCCGCGTGCTGGGTGCTCTGCCCGAATTGCGCTCTTTGTCCGTGCAAGGCCTCCAACGCATCTTCTCCTTGAAGCTAGAAGACTTGGTCCCGGCACCACCTCTTATCGAAAACATGTTTCGTGCCAGCCTTCCCTTTTAG

**>Bm_nscaf1898_501**

ATGCCACAAACCGTGCCGCGGTCCGGGTGCAGCCCGCCTTCAGCATCACAAGGCGAGAGAGCTGTCCCCACGTCTCCAGCTGATCTACAGCACCTGCTCCGTTTATTAGGGGCCTCGTCGCCGCCAGAATCCATGCTCCACTCTCCACCGAACCTACACACAAAACCTCCGCCACCATATCCAGAGGATAATAACTTCTTGGACAGGCTGTACGATTTTGAAGGATATCCTACCCCGTCTCCTTCGTCAGACGAAGGCTCGATTCCCACGGTGGCGCTGCAACCATCTTCCCCGTACAGTTCACTGCACTACACAACACCAGTGTATATCAAAGAAGAACCAAACAGACTGTCTGTGCCTGGATTTGCCTCACCCTACCCTCTGTCGCCTTCTGGATCCTGTGTATCTTATGGAAGCCATAATCAGTATCCTTCGCCTACACCTCAACCCGAAGAATATATTGACATCGAGCAATTATTAAAAGAGAATCAAATATTACAAGACAGCACACAGCATAGTTACATAACCCCTAAAACAGAGATAGAAGAACCAAGAGATCATATATTATTGAGATCAGTACTCGAAGATACCACATTCCAGAAGAGATTCAATCTTAGACCTGTGCCTTTAGAGTTGGGAAACGTGAAAATGGAGGAGAGCAGTGGTGGTGACGACCTGGTGGCACCGGATATTGATCGTGTGCTGTCGATGGCCATTGAACAATCTAAGAGGGATGTGGATAGCACTTGCACAGTCCTCGGAATATCGCCAGATCCAATGCTTTGGAGTACAGCGGATGTCAAAGCTTGGGTGATGTTTACATTGCAACATTTCAACCAGCCATTAGTACCGTCCGAGTATTTCAATATGGACGGGGCTGCACTGGTGGCTCTTACAGAAGAAGAATTCAACCAGAGAGCTCCTCAGTCGGGAAGCACTCTGTATGCTCAGCTGGAAATATGGAAGGCAGCAAGACACGAGTCGTGGAAGACTTCTCAGTGGACTGACCATCAACCTTCGCCTACACCTGTACCGGCCGCGTTACCCTCAGCCGACGATATGAGTGAAGGTGCGTACACTAAAAGATATTTTTAG

**>Bm_nscaf2859_001**

ATGTCGTGTGGCGCTGAGTTGCGCGAGCGGCACTCGGTGCTAGTGAGCATGCTGGAGGCTCGCCGCGAGTCCAGTGACTCGGGCTGCTCGAGCGACGACAGCTCCGACGTCGAAAGAGACTCTACTAAATGTAGCTGTGACCCGCAGGGCTTCTTCCGGCGATCTATACAACAGAAGATACAATACCGGCCCTGCACGAAGAACCAGCAGTGTAGTATCCTCAGGATTAACAGGAATCGGTGCCAGTATTGCCGACTGAAAAAATGCATCGCCGTCGGAATGAGCAGAGATGTTGCGAATAAAAGGGGAAGTACAATGATCGACGAGAAACAATGGGATCTCGCATCTGGTGCGGCCCACATTGAGCAGCGAAAGTTAAGAGAAATGGCGAGAGGGCGCTCGCTCTCTGCTTTCGTTTTCAACCCTGTGCGATTCGGTCGTGTACCCAAACGCGAGAAAGCGCGTATCCTCGCAGCGATGCAACAGTCGTCGTCGTCTCGTGCACACGAGCAAGCAGCTGCCGCTGAACTTGATGACGCTCCTCGGTTGCTGGCGCGAGTGGTGCGCGCTCATCTCGACACGTGCGAGTTCACGCGTGATCGCGTCGCTTCCATGCGAGCCAGAGCTCGCGACTGTCCCACCTACTCGCAGCCTACTCTGGCTTGCCCACTAAACCCGGCGCCAGAGCTGCAATCTGAAAAGGAATTTTCGCAACGTTTCGCCCATGTGATACGTGGCGTGATTGACTTTGCCGGCCTCATCCCTGGCTTCCAGCTGCTGACCCAAGATGACAAATTCACGCTGCTCAAAAGTGGTCTGTTCGATGCATTGTTCGTGCGACTCATCTGTATGTTTGACGCTCCTCTTAATAGTATCATCTGTCTCAATGGGCAACTGATGAAGAGAGACTCCATCCAGAGCGGTGCCAATGCAAGGTTTCTCGTTGATTCTACTTTCAAGTTTGCGGAACGTATGAATTCCATGAATTTGACGGACGCGGAAATAGGACTTTTCTGTGCTATAGTCCTCATCACTCCGGATCGGCCTGGCCTGCGAAACATAGAGCTAGTGGAAAGAATGCACTCGCGACTGAAGGCGTGCTTGCAAACTGTCATTGCACAGAACAGGCCAGAGAGACCTGGGTTTTTAAGAGAATTAATGGATACATTACCTGATTTACGCACTTTAAGCACGCTTCACACAGAAAAACTTGTTGTTTTCCGAACGGAACATAAGGAGTTATTGCGTCAACAAATGTGGAATGAAGAAGAAGGTGTTTCCTGGGCCGATTCCGTAGTGGAGGAATCAGCTCGTAGCCCCATCGGGTCTGTATCCAGCAGCGAGTCCGGGGAGGTGCCGAGTGACTGTGGCACTCCTTTACTGGCAGCAACGTTGGCCGGTCGCCGGCGACTTGACTCTCGGGGCTCTGTCGATGAAGAAGCTCTCGGCGTCGCACATCTGGCTCACAACGGACTCACCGTGACGCCCGTCCGCCCTCCTCCCCGGTATCGGAAGTTGGATTCCCCGACTGATTCGGGTATTGAATCTGGCAACGAGAAACACGAGAGGATAATCGGACCCGGGTCGGGTTGTTCTAGTCCGCGGTCGTCCTTAGAAGAGCACACCGAGGACAGACGGCCCACCGCGCCCGCCGATGACATGCCCGTGCTCAAACGTGTGCTTCAGGCTCCACCTTTGTACGGCGGAACTTCTACATTGATGGATGAAACCTACAAACCACACAAGAAATTCCGCGCTATGAGGCGCGACACCGGAGAAGCAGAGGCTCGTCCAGTGCAGCCGACGCCGTCGCCACAGCCGCTGCACCCGCACCCGGCCAGTCCGGCTCATCCGGCTCATTCGCCGCGACCACCGCGCATTTCTCTGTCATCCACGCATTCGGTGCTCGCTAAAAGTTTAATGGAGGGGCCGAGAATGACTCCTGAACAATTAAAACGTACCGATATGATCCAGCAGTACATGCGGCGAAATGAAGCTGGTTCTAGTGTGGAAGGATGTCCGCTGCGAACTGGAGGGCTGCTGACCTGCTACCGCGGTGCGTCTCCGGCTCCGCCGCCAGTGCTGGCGCTGCAGGTGGACGTGACGGACGCGCCGCTGAACCTCTCCAAGAAGTCGCCGTCGCCGCCTCGTTCGTACATGCCGCAGATGTTAGAGGCGTGA

**>Bm_nscaf2876_46**

ATGCCACACTGGGTAACTGCTGAAGAAGGCACTGACACCGAATCTCCTGACCAAGTTATGTTATATTACGAAGATGACGCTTCAGAATACTATGATAATAAACCCGACGGCCCAGACAATATAGAAGAACAAAATGGAGCCATCAAGAAGCACGAAGCAGATAAGGGCATTACGAAAATCACCCGTAACCATTTTTATGATAGTGGTAGCGGAAGTGATGAGCCAGTGCAGCGCAGGATGCGCCCAAGACGCGCTGTAGTATTAGGGTCTTCATCATCAGCCGGCAGTACGGGACCTCCTTCAGGACCCGGTCGACGGCGGAGGTGTGGCATTTCCGCTCGTGAACGCAACCTCCGTCGTCTCGAGAGCAATGAAAGAGAAAGAATGCGAATGCATTCCCTCAACCGAGCTTTTGAGGATTTACGCCGCGTAATCCCACATGTGAAAAAAGACAACAGAAGTCTTTCAAAGATAGAAACCCTTACCTTAGCCAAAAATTACGTGAAAGCCCTCACGAATGCTATTTGCACAATGAGAGGTGAAGTTGCTCATTATTCATTCAATAGCGACGATGAAAACGTGGAACCCGCATTTGTATTGAATAGGAGGGATCAGGAGCCGAACAACAACGAGACTGTCAGTACGGACCAGCAAGAAATTACGACACGGACGCAAGGTTTCTTCTGA

**>Bm_nscaf2734_12**

ATGACGCTTGTAGAGCGGCGCCGTATGTCGATGTGCACGTTGAGACTAGCGGAGGGTGCGGTTTCAACTGTTATGGCCCGGCCCGGTAACACGACGCCGTCTGCTCCACCGGCAGGCTCCGACACGGACCCGTTTGGCCGACCACTTGCGTATGCACGCGGAATTCAATTGCTATTCGATCGGTTCGTCATGTCTCACTTAGGGAGCAAGTCCGTCAACACTAAGGAGACGCTCGAAGACAAGAAGGATGACAACGACCTCTGGGAGGCGCAGGCCGCCTTCTTGGGTCCCAACCTGTGGGACAAGACCCTGCCCTACGACCCCGATCTCAAGTACGTGGACCTAGATGAGTTCCTGTCGGAGAACGGCATGCCGGGCGAGGGCCTGGGCAGCACGCACCTGGGCGGCTCCGCGTTCGGGCCGGCGCTGGGCCTGCAGACTCCCATCACCAAGCGGGAGCGCTCTCCCTCGCCCTCCGACTGCATGAGCCCGGACACCATCAACCCGCCGCTTTCACCAGCCGATTCCACGTTCTCGATGGCATCGTCGGGCCGTGACTTCGATCCTCGGACGCGCGCCTTCTCCGACGAAGAGCTGAAGCCGCAGCCGATGATCAAGAAGTCCCGGAAACAGATACCAAAATTACCATGA

**>Bm_nscaf2847_349**

ATGCTTGTAGAGGACTCTCAAAATCGTTCAGTGCTGGCGAAGAACTATGCGCACTGTCCGCTGAAGAAGCGACCGGTGTTGGTGCGGGAGGAGCGGCCTGCGACGCCGCCCACTACGCCGCCTCATCCCGCCCACCTTGCCACGAGACTTTATTATGATTATCATTGTGACATGGAAAATGAAGAACCTGAGAATCTTAGCACTAAGCCCGAAGACCTGTCCAAGACAGGAAACTATCCGAGCAAAGCTTCGTCGCCAGTTTCAGCGATGACGGTTAAGGTTGAACCAAGAGAATGGTCTCACCAGCTGCCCGACTATATGTCAGCTTGCCGAACTCGCCTCGAGCCGGCACCAACAGAGCTTGCACGACCTACGCCGCAGTACCCTTACATGCCGACATTGTATTCGCCCTACGCATTTGAAGAATTATACCCAGCGGCACCTGCTCTGTCCCCTCCGATACACCCGCAATTGTATACGCGTTACTCACCCGCATCACCGCCGTCCTCGTGTTCACCTCCACCTTGCCCCGAAGATCTAAGATCACCCGGTTCCGTTTCTTCCGACTCGGGAGTTTCCGTTTCGGGAACACGTCGTCCTCGGTATCAATGTTCAGACTGCGCAAAGTCTTATTCGACGTATTCCGGTCTCTCGAAACATCAGAAATTTCACTGTGCTGCTGCGGAAGGTAGTCTAGCAAGGAAGTCTTTCAGCTGCAAATATTGTGCAAAGGTTTACACTTCTCTTGGTGCACTTAAAATGCACATCCGCACGCATACGCTACCCTGTAAATGCCATTTATGCGGTAAAGCCTTTTCAAGACCCTGGCTTCTTCAGGGTCACATCAGAACGCACACCGGTGAGAAACCATTCTCTTGCCACCACTGCAGGCGCGCATTTGCCGATAGATCGAATCTTCGCGCTCACCTTCAGACCCATTCGGATGTGAAAAAATATTCGTGCTCTGGTTGTGGTAAAACCTTTTCACGTATGTCCCTTCTGAGTAAACATCTGGAAGGCGGATGTGGAGTACCAGGCAGCACGCCTTACGAGTATCGTCCTGAAGCAATGCAAGTTGCGCATGCGCACCCAAACCTCCCACCGCCCGCTACCGTGCACGCCTATTAA

**>Bm_nscaf2888_249**

ATGGTCGTGCGATGCGAGTCGGCGGCCGACACCAGCACTTCGTCACCTGACCCAGGACCGCCTTCGCCGCGTATGTCTGAAGCCGGGTGCAGCACGCCGCCGCACCCGCCGCCCGTGTTCGATGGCGGTGGCTCTCCGTCACCTTCACCCGCTTGCCATCCCACCGTTATACGCTCGGCGCCACCCTACTCAGTCATCAAGTTTGAAGGTGCACAATCCGCTGTCAAAGCAGAAAGTTCGCCTGCGAGTGGAAAAAACCAGCCCACAACGCAGTCTCAGTTGTCTTCAGTGAAGTTGGAAAGCGCACCGCAAGAATCACCACAACCGTTCAGACCACGTACCCTCGTACCGCCTCCGCCTGGAGTTCACGCTAATCTATCACCAGGACATTGGCCGCCGGCCGCTTGTATAAATGGTGTTAAACCTGAACTTATAGGCGGAAATTTTCCACCACAGCCGATTGAAAATAAACCAGGTGCGCGTGGACAAACACAATGGAGAGGTACTCCTGCTGTTATAATGGGCGAATCCGGAGGTGTCCGAACAATGTTCTGGACCTTACCAGCGCCTAGTTCAAGTAGTGAGCCTGCAGCCAGTGCTTCGCATACTTCATCCAGGCCTTCTCCTGATCCAGCTTCATGCAGTGAGGAGTCAGCAGCGAGGCTATTACTCAATTTAGGTGGCGAGTTACGACGACCTAGAGGGCCACCATTGAACATGGAACTACTCTGGGCCGGAGACGTGTCCCAGCTCCCTGCTCACCAACAAATTCATGCGTTAAATCTGAGCGCCGCTGCCGGAAGCATCGCGGGTTCTTCTCCAATGGCAGGTGCTAGTTCCTTAGCGTTACCCAGACCAGAACTGCGTACATATGCACCAGAGACTGAACGCGATGAAGACGAACAACCAATGATATGCATGATTTGCGAAGACAAAGCGACTGGACTGCATTACGGCATAATTACTTGTGAAGGCTGTAAGGGATTTTTCAAACGAACTGTCCAAAACAGAAGGGTTTACACGTGCGTCGCTGACGGTGGCTGTGAAATTACAAAGGCACAAAGAAATAGATGCCAGTATTGTAGATTCAAAAAATGTATTGAACAAGGAATGGTTCTACAAGCTGTACGAGAGGACAGGATGCCTGGTGGGAGGAACAGTGGAGCTGTTTACAATTTATACAAAGTGAAATATAAGAAGAACAAGAAAGCCAACAAAACAGCTACGGCTACAAGTCGAGCTTCGCCACCAGAAAAACCTAAAGAACCCTTACCCCCACTCCCACCGCATTTGGTCAATGGTACCATACTTAAGACTGCACTAACAAATCCCAGCGAGGTTGTTCATTTGAGAGCAAGGCTTGAAAGTGCCGTGTCGTCGTCACGAGATCGAGCCGTCCCTTTGGACAGGGCGCTGCACATGATTCGCGCTCTGATTGACTGTGACGCTATGGAAGACATTGCGACAGTACGACACCTCCCTGACTTGCTTCATGACACTTCGGAAATAAGCGACAAATTGTGTAAGATCGGTGACTCCATCGTGCACAAAATGGTCGCGTGGACGAAAAAACTACCGTTCATTATGGAAATCCCTATGGAAATACACTCAAAATTATTAATGGAAAAGTGGCATGAGATCTCAGTATTAACGACGGCGGCGTATCAAGCGATGCACGGAAAGCAGACCCACGCTCCTCCCTCGTCAGATCACGAACAGGACTTTATGCAAGAGGTAAACGCCAACCTCCGGACATTGCAGAATTGCTTGACGTCACTTATGGGCAGGCCCATTACGCTGGAGCAGTTGCGATTGGATGTAGGACTTGTCGTGGAAAAGATGACGCAGATAACCTGTGTTTTCCGTCGCATACAGCTCCGAATGGAGGAATACGTCTGCCTCAAAGTCTATATACTGCTTAATCAAGGTAAGAATTTCTTAAAATTATAA

**>Bm_nscaf2902_040**

ATGCCCGAGCACGACAAGTACCAGCTGCGGCCGCGGGCTGCGAGGAGCCGCGAGCCTCGCGCCCGACGAACTCCTCAACCCCTCAGTAAATACCGGAGGAAAACAGCGAACGCAAGGGAACGAAGCCGAATGAGGGAAATCAACCGAGCCTTTGAAGCTTTAAGACGCGCTGTACCCGCTTCCGAGATCACGGGAACGCCGGTTCCGTGCGAGAAGCTCACCAAGATCACTACTCTGCGACTGGCTATGAGATACATCACGGCTCTGTCTGCAGCCTTGAGGGACGACGGCCCTGAGACGGAATCAACTCGGCCGGATCGATGGCCTTCGCCTCTTTGCTCGGATCTGTCGGAGTTCTCGACGGAACTGGACCAGGAGACTCCCTCCGAGTTCGCGGAGGACTCGTTGTCTCCATTCGATTCGTTCTTTGCGGAGTTCGACTGTGATTACATCGAAAGGACATTTGCGTGA

**>Bm_nscaf2964_066**

ATGGAGGGTAGATCCCAAATCGAGATAATACCGTGCAAGGTATGCGGAGATAAATCGTCGGGGGTGCACTATGGCGTGATCACCTGCGAGGGATGCAAAGGATTCTTCAGACGATCCCAGAGCACAGTGGTGAACTACCAGTGTCCTCGCAACAAGGCCTGCGTCGTGGACAGGGTCAACCGCAACCGATGCCAGTACTGCAGACTACAGAAGTGCCTCAAACTCGGCATGAGTCGTGATGCCGTCAAATTCGGTCGCATGTCGAAGAAGCAGCGGGAGAAGGTCGAGGACGAGGTCAGATACCACAAGGCGCAGATGCGGGTGCAGGCTGATGCGGCGCCGGACTCCGTCTACCCAGGATACGGGTCGCCGTTGTCTTCGTATGGCTACAACAACGCCGGGCCAGCGCTACCCTCGAACATGAGCGGGATGCAGCCGCAGCCCCCAGCTCAGCCCCCGTACGAAGTCTCAGGCGACTACGTGGACTCCACAACGACATACGAGCCCAAACAGACAGGGTTCTTGGACGCAGACTTCATAAGTCACGAGGAGCGCCAGAAATCGACCATCGTCCGACCGTCGACCGCGACCACGACCGCCACCACGACCACGATCCGACCATCGGCCATAAACGAGCTGCCCAGATCACGGCTGCAGGAGTACGACCGGTACGACGACCGGATTCAGTCGCCGTCAGGGGTCATCAGCATTAAGCAGGAGATCAAGCCTGAGACTTCAATGGGCGTCGATAACTTGGTGGCGAGCTACGTCGACTCGACAACGTTCCTACACAGTCCTTCTAACATGCAAAACAGCCCGATGGACATACAGAACACGGTGCTCGTCAGCGGCCAGAGCTCCGTCTCATTGACCAGCGAGGACTTGAGTCCTGATGATTTGACTTCGAGCAGCGGCCACGAGAGGCTAATGGACCCCATGAACATGAACATGTCCGCCATGGGAATGGTGAACCCCAATATTGTGTCAACGAGAAGACACCACGGCGCTAATAATTCTAATGACGATATGCCTTTGGAGGGTGACATTAGCAAGGTGCTAGTGAAAAGTTTGACAGAGGCGCACGCGAATACAAATCCGAAGTTGGATTACATACATGAGATGTTCGGCAAGCCCCAGGATGTTTCTAAGCTCTTGTTCTATAACTCCATGACCTACGAGGAGATGTGGTTGGACTGCGCCGACAAGCTCACCGCGATGATCCAGAACATCATTGAGTTCGCGAAACTCATACCTGGTTTCATGAAGCTCACCCAGGACGATCAAATACTGCTGCTTAAATCAGGTTCGTTCGAGTTGGCGATCGTCCGTTTGTCGCGGCTAATCGACGTGAACCGCGACCAGGTGCTCTACGGAGACGTGGTGCTACCCGTGCGGGAATGCGTGCACGCGCGCGATCCCAGAGACGTAGCTCTGGTGCAAGGAATCTTTGAGGCTGCCAAGAGCATCGCTCGACTGAAGCTGACCGAGACTGAACTGGCTCTATACCAGAGCCTTGTGCTCCTGTGGCCAGAGCGCCACGGCGTGATGGGCAACTCGGAGATCAGGTGTCTCTTCAATATGTCCATGTCGGCGATGCGGCATGAGATCGAGGTCAACCACGCGCCGCTCAAGGGTGACGTCACCGTGCTGGATACACTCCTGGCCAAGATACCCACTTTCAGAGATCTCTCCCTGATGCACCTCGGAGCGCTGAGCCGTTTCAAAGCGACGCATCCGCATCACGTTTTCCCAGCTTTATACAAAGAATTGTTCTCTTTAGACAGTGTTTTAGATTACACGCACGGATAA

**>Bm_nscaf3090_4**

ATGGAGTCTCCCCAGATGTACGATGCGGCGGCAGCGCCTCCGCCGCCTCCCCAACCAGACCTCAAAAAAGTCGTCGAGGATAAAAGATCGGCTTTCCCACCACCGGAACTCGACGAGCTCAATGGACAGGAGATCAGCTTGGATCTGCAACACCTCATCGAGGATCAGTTCCGGGGCGAGGAAACGATGGCCCTCTTTCAGGAAATCCTACCCGGAGGTCGATCGCCTCAGCCCCGGTTCACGAGGACGACGCTGGCGTACATGCCGCAACCGGTCCACTCCGGAGCGTCCTACGCGCCAGTACAAGCCAGCTCGGCACACGAGCAGGCGCCCCCAATTAAAGAAGAGCCGCCAGAACCGCAGGACTTCCGAAGAACGGTCACATGCTCGCAATACACAGGACAATACAATCCGCAGCCGCCAGTCGGCGTTAACAATCCTTACACAGGAAGTTTCACACCCCTACCGCCTCTGGGAGGACCTCTGCTTCCTCCTCTGCTGAAACACAAACCGGCACCTCCGAGACGATCCTCAGGCAAAGTAATAGACAAGGGAACTGACGAATACAGAAGGCGAAGGGAGCGCAACAACATAGCCGTGAGGAAATCCCGGGAAAAGGCTAAAGTACGTTCCCGCGAAGTCGAAGAAAAAGTGAAAACATTGCTGAGAGAGAAGGAGGCCTTGCTGAAGAGGCTCGAGGCGGTTTCGGGGGAGCTGAGCCTCCACAAGCAGATGTACGTGCACCTGATAAACTTGAACCACCCGGAGATCACGGAGCTGTGCCGGTCGATGCTGCAGCTGGGAGGCCCGCACTCCCAGGACCACACGCTTTGA

**>Bm_scaffold316_2**

ATGTTAAGTTCGTTCTTCGGGCGCACGTACAACAACCTGTCGTCGATCTCCGAGTGCAAGAACAACGGCGAGTGCGTCATCAGCGGGAAGAACAGGACGGCGTGCAAGGCGTGCCGCTTACGGAAGTGCCTTCTAGTCGGAATGTCCAAGTCCGGCTCTCGGTACGGCAGGAGGTCCAACTGGTTCAAAATCCACTGCCTTCTCCAAGAGCAACAGCATCAACAGATGCAGCACATACAAAATAGAAAATCTCCACCGACTTTCAACACCTCCATGAATGCATCTTTCTTGCCCACAAACTTGCTTCCGGCTGCCGCCCTCGCAGAATATTACAAAAATTCAGAAAAGAATCCCTTCACAGAAGATGTGATGCGGCAGAGTGTTTCACCTTCAGATTCCGGAGCATCTTCCGCGGACCCTGAAGATGACAACAGTTCAAGAAGCACTAGCGGCTTGAGCATCTTCCGACCGGCATCGTCACCGCTGAGCGATAAAGATGTCAGATTACAGGCCATTAGGAATCAAAGTAAAGATGTCAAAAAACGAAAACCAGCCTTGCCCTCTCCGTTCAGTTCTATGTCAGCATCACCAAGCTTTTCACCGAGACCTTTCCTTCCAGTGATGCAAAGACCGATTCCACCGGCGGCCACCACGTGGTCGAACAGAAATGGTGGCGACTTGCTCCTACACTCGCCAGCCGTCGCGGGGGTGGCGATAGATCAAGACCAACCCATTGATCTATCCATCAAATCGACCGCAGTACTAATTAGGAGCCCCAAAAACGACGAAGTGAGCGACTCAGAGCCCGAACTGAGCATCGACTTGAGCGAATCAAGCAAAGACATGATGAAGAATCCTCTGGACCTTTCTCTTGTTCCCAAAAGGACTGAAGAGCTTCCATTAACTGGTTGA

**>Bm_nscaf1898_167**

ATGCTCCAAGAAATCCAGCTAGTTCAAGGTCAGACGAATTACGTGGTCGTTTCTTCCGGATATCCTTCGAATACATTGAATAAATCATCTTTAGAGAAACGAAATGTTGCTATAGCTCCAGCGCCTGAGAAAAATTACGTCACACACGACACTCCACCGAATCTACACTACAGGAAAAAGGTGCATTTCAGAACTAATCCGTACACTGGACCACAGGCTGCGTCAATAGCGAGACGTAATGCACGGGAACGAAATCGCGTAAAACAAGTGAATGATGGATTCAACGCACTTCGCCGTCACCTGCCGGCTTCTGTCGTGGCAGCTCTGTCCGGTGGCGCCAGACGAGGTTCGTCAGGGAAGAAACTTAGCAAAGTCGACACATTACGGATGGTTGTTGAATATATAAGGTACCTTCAGCAGTTATTAGACGAAAGTGATGCCGCATTAGGTATTACGCGTGATCAAGAAAATCGGGAAAATATTCCAAGCAATAACTCAGTTCAGCCGATGACTTCTATTGACATGGATGACGGGTTTTTCTACGGAAGTGGATCACCTTGTTCAGAGAAGGCAGATTCGCCAGCTCCTTCGGAATGTTCTTCGGGTGTGTCTTCGGCGTATTCGGCTGTCGATCGTTACGAGGTTACTACGCAGCAACAAATGGGATCAATGGATGAAGAAGAACTCTTAGACGTCATTTCATGGTGGCAACAAAAATAG

**>Bm_nscaf1898_212**

ATGAAAGTTGTAAGCTTACAACTGCCGTCTGGGCCCAGTATGGAGCGGCTACCGCTACCATTCAGTCCAACAGAACTATTATGGCGTTACCCACTACCCTGGGCGCCACCGCCTCCTTCACCGCTTGGTGACACCAAGGCGCAACTGCCAGCCGGTCTTCCGCCAGAACCAAGACTATGGACCAGAGAGGATGTTTCTGTATTTCTGAAATGGTGTGAAAGAGAATTCGATCTACCTAATTTTGATATGGACCTCTTTCAAATGAACGGTAAAGCATTATGCTTGCTTACTAAAACAGATTTGGGTGAAAGGTGTCCTGGAGCAGGCGATGTCTTACATAATGTTCTTCAAATGCTTGTACGCTACGCAGCGCTGTTAGGTAGAGTTCCTTCCTCACCTGTTACTCCCACCGCTCGTGCGGCACCGTATCCGCCCTCTCCCCACTCTCATCCACCGACACCAACTTGGACTGTTGATGGTTTCCACCATTTCCACAGCGCTGCAGCAGCAGCAGCAGCTCAACCTAATTCAGTGACTCTCAGTCCTGCTCCATCAGTTGATAGTTCTGGAAGTCCTCAAAGAGGAGACACTGTAACTTATGCGCCAGCGTATGCACCGTCCGTGCCCCCCACTACTCAAGCTGCCAGTTCCGGAAGTAATCATTCAGATTCCGATGAAGAGGGTCAATACGCTCCACCTCCACGTTCTCCTAAAGAAGCTCCAATAACGAGTCCAGCCCCGCAAAATCATCCAACACAACAACATCCTCATTACCGCGCTCAACATAGGGAATTTTTTCCAAATGATATGCCAGAGTCCAATACAAATGGAAGACTATTGTGGGACTTCTTACAACAACTTCTGAATGACCCAACGCAAAGATACACAAACTATATTGCATGGAAGAACAGAGACACTGGCGTGTTTAAAATTGTTGATCCTGCTGGACTAGCCAAATTGTGGGGAATTCAAAAAAATCACCTATCCGTGAACTACGACAAAATGTCCCGCGCCCTGAGATATTATTACCGTGTTAATATTTTGCGCAAAGTCCAGGGCGAAAGACACTGTTACCAGTTCTTAAGAAACCCAACAGAATTGAAAAATATCAAAAACATATCTCTACTGCGACAACAAATGAGTCCCACTCGCGTTGTACCACAAACTTTGGTAAAAACCGAAATGAAAGAAGAAAGATGTGACGAAGAAACAGTCGATGAAGAAATGCCTACAGATTTGAGTATGTCTGCATCAGAGCCTTGGCGGAAACGCGCACGCTCTGACACAGCGACCGCACCGCCGTCGTCAACGCAACATGACAAACATCGGATCAGTACGCTTATCGGAGATAACATGATAATGAAACGCGAAGTCGACTACAGTGCTGAGCATTATGCCTTAAATTTAAAAAGTGAAAAATGTGAACAGTAA

**>Bm_nscaf2770_58**

ATGAACGCGTTCATGGTCTGGTCGCAGATAGAACGCAGAAAAATATGCGAACAAACACCGGACATGCACAACGCCGAGATATCAAAAAATCTCGGACGCGTTTGGAAAACTCTGAACGACGAAGAACGCCAGCCCTTCATCGATGAAGCCGAACGCTTGAGGCAGCTTCACATGAGGGAATACCCTGATTACAAGTACAGGCCTCGCAAGAAAACCGCCAAGACTGACAGAAGCGGTACGCGAACCGGTTCAGTGCAGAAACCGAAACGGAAGCAAAGAGCCGATTCCAATAACAACAGAGGACTGTCCAGGCGACGACGACCCGTGCCGACTGTCCCGAGCGTCCCCGTGGAAACGCCGGCCCCTCCCCCGCTGCCCGCCTCTCCTGCCGGTGCACCTGATTCCCCGGAATCCGCCTGTCTGTATGATGACCACAACAGGCGCGATCAGGCGGATCTGACGGATCTCTATTCCATCACGGATCTCCTTCCTCTCCCGGCGGACTGCGAAGTCGACTTAGATGCGCTGACGACGGACATCGACTCGTTCGAGACCGCTTCGTCCTCGTCTGGATCCCACTTTGAGTTCTCGTGCACGCCTGACGTCTCGGACATGCTCAGCGAGATCGGCGTAGCGGGCGACTGGGTGGACCACACGTTCTCCTCGTATCTGACGTCATCTTAG

**>Bm_nscaf2847_110**

ATGCAGGCGGCAAGCGGCGACCTTGTCGTGGCTCGCGACGTCACGCGGGGGCGCGCCGATGACGTCACGCTGCGGCCTCGCCGTCCGTCACTCGGTGTGCACGTGAAGAACACGCGCGACCACGAGCCGCCTGCTGCAATGGAAAGTATGACAGCAGGAGAATACGCAGCGCGATCACGCAGCGCGCGCGTGCTCCGCCGTGTCGGTGGACGTTCACTTATGTGCAACTATCTTGTCTCACAGGTCGAGCTGCCTCTCAAAAAGATTGCCGCAAAACGGGCGAGAGAGTCGTCGTCGCCGCCCGCCGGCCTGGTCACGCCCCAGCCCTCGGACTCTGAAGGAGAAGACGAGTTCAGCAAGCGATCCCGATGCGAGCTGGAGCGCTTGCTTCTCACGACGCCGCCCCCGGAGCCGTGCTACAGGCCCTCGGTCATCATGCGGGCCCACAAGGACGGCACCTGCAGTCCGGAGCCTCTGCCGCCCCCGCCCGCGCACGGCATGAACATGCTCAAGACACTTAAATTTAAAATGAACCGAGGACACAGTTATTCACAAACCAAATACATTGCGAGCCAAACGTCTCCTCCCGTTCCGGTCGCCGTTCAACCTCCCAGTCCCCCACCGAAGATTGAAGAAGCTCGCGTACCGTCACCCCACCTGGAATGTCAACCGAAACAAAACACGAGAGACGAACAGCGCGTCCCCCAGGCCCCGTCGCCGGCCGCCACCCCGGGCTGGGTCCCGCTGGCGCCGAGACCGGCTCCGGCGGTCCTAGTCCCGGGGGCGCTGCTGCCCACCGCGGCGCTGTGGCTCGTGGCGCCCGCGCCCGCCGCCCGGCGCCGACTCTACGAGTGCTCGTATCCCGGATGCGGGAAAAATTACTTTAAGTCGTCACACCTGAAAGCGCACGCCCGCACGCACACCGGGGAGCGACCGTTCCGGTGCGCTTGGCCCGGCTGCGAGCGGAGGTTCTCGCGCTCCGACGAGCTGTCCCGGCACAAGCGCACGCACACCGGCGAGAAGAAGTTCGAGTGCCGCGTGTGTAACCGCCGCTTCATGCGCTCCGACCACCTCGCTAAGCACGTCAAGAGGCACGCCAAAGAGAAGGCGCCGCTGACCCCGGTGCTGCGGCTCCTCCCGTCTCCTCCCGTGGCGCTCCCGGTGCGGCCCGTGGTGGCGGCGGCGCAGTGA

**>Bm_nscaf2888_433**

ATGGCCGTGGGAGCGTTGAGCGATCGTGACGCATATGGATATGGTTACGGCGGCGGGGGAGGCGGGACTCACCACTTCGCAGCGCCTGCGCCCCCGCCGCTGCCGCACGACGAGCTGCCATACTCCGTATTCGACTTCGGCGACTACCAGCGCCACCATCATCACAACAAACTGAAGCCGAAGAAAAGGCCTCGCTCCGATGCACCTCCCACGCCGGGCGTCAAGCGCAAGAGCCGGGAGGGCTCCACCACGTACCTGTGGGAGTTCCTGCTGAAGCTGCTGCAGGACCGCGAGTACTGCCCGCGCTTCATCAAGTGGACGAACCGCGAGAAGGGCGTGTTCAAGCTGGTCGACTCGAAGTCGGTGTCGCGACTCTGGGGCCTGCACAAGAACAAGCCAGACATGAACTACGAGACCATGGGCCGCGCCCTGCGCTATTACTACCAGCGCGGGATCCTCGCTAAGGTGGACGGCCAGCGCCTCGTCTACCAGTTCGTGGACGTGCCCAAGGACATCGTCGAGATCGACTGTTCGTTGGCGTAG

**>Bm_nscaf2930_060**

ATGTCATACGAGATCGCGTATTCGGTCGCGGAAGACGGTCCGCAGCCAGTCTCGCGCACTTATCAATATCGCAAAGTGATGAAACCGATGCTTGAGCGCAAGAGGCGCGCTCGCATCAACCGCTGCCTCGACGAACTCAAGGAGCTGATGGTCAGCGCTCTACAATCTGAAGGAGAGAATGTAGCCAAATTGGAAAAAGCTGATATCTTGGAACTGACCGTTCGCCATCTTCACAAGCTGAGACGCCAGCGTCGCTTGTCACTCAACCCTACCGTGGATGTGGATCGCTTCAGAGCTGGATTTACTCACGCCGCCAACGAAGTTTCCCGCTGTCTGGCTTCAATTCCTGGTGTGGACGTGAGGTTGGGCACTCAACTAATGACCCACCTCGGGCACAGACTGAACGAAATGCAGCGAGCAGCCGCTGGCACTGAAACTCCACCAGCAAGCCCTCCCAGCCCTGCTCTCTCGACTGTTTCTTCATCGTCTGGTTACGTGAGCCCATCTCCTCCGGCGTCACCGATGCCTCTACAGACAGCAACTCCATTGGACTGCAGCATGAACACCTCACTGCCCAAAGCATCGGCTGTGTGGCGGCCCTGGTAG
