# Supplementary material for: Transcriptomic analysis of developmental features of Bombyx mori wing disc during metamorphosis
Source: BMC Genomics. 2014 Sep 27;15(1):820. doi: 10.1186/1471-2164-15-820 (PMC4196006; doi:10.1186/1471-2164-15-820)
Supplement: Supplementary file 7 — Additional file 7: Assembled nucleotide sequences of transcripts in Table 5. (DOC 34 KB) [file 12864_2014_6525_MOESM7_ESM.doc]

**>Bm_nscaf2912_25**

ATGATAGCGCTCGTATTCGCGGTGGCCGCGTTCGCCGTCGGCTCCTGCGGCCCGCCAGGGAAACCGAACCTCGGCTGGGGCGAGCGCACGTTCGCGATCGTCGAAGTCAACCAAGCGGCCACAGCTTACAACCAGCTCGTGACCAAGAAGGACGCCGCCGACGTCTCCGTCAACTGGAACGTGTGGACCGGCGACGCGGCCGACAAGTCCAGAGTCCTGCTCGATAAGAAGGAAGTCTGGAGCGGTGCCGGCAGCGCCACATCGGCCGCTTTCAAAGTGAAGAAGGGCGGAAGATACCAGATGCAGGTCGAGCTCTGCAACTCAGACGGATGCAGCTCTAGCGAGGGCGTCGAAATCGTAGTAGCTGACACTGATGGTAGCCATTTGAGGCCTCTCGATTATTCTATTGGTGAGAAAAATAAGCCCTTCAAGCAGACTTCGGGAAAAGTGGTGGGCGCTTACTTCGTCGAATGGGGTGTGTATCCTAGAAAGTTCCCTGTGGACCGCGTTCCGGTTCCGAACCTGACTCATTTGCTCTACGGCTTCATTCCGATTTGTGGTGGTGATGGCATCAATGACAGTTTGAAAGAAATTGAGGGCAGTTTTCAAGCACTACAACGTTCATGCAGCGGCCGTGAAGACTTCAAGGTTTCTATCCACGATCCCTGGGCGGCCCTGCAGAAACCTCAAAAGGGATTATCGTCCTGGAACGAGCCCTACAAAGGTAACTTCGGGCAGCTGATGCAGTTGAAACAAGCGAATACTGGCTTAAAGGTCTTACCTTCAATTGGTGGCTGGACATTGGCCGATCCCTTCTTCTTCTTCACCGACAAAACCAAGCGTGATCGTTTCGTTGCCTCCGTGAAAGACTTCCTCCAAACTTGGAAGTTCTTCGATGGTGTCGACATTGATTGGGAGTTCCCAGGTGGCAAAGGCGCTAACCCTGATCTCGGTAGTCCTAAAGACGGAGATGTATACGTTCAGCTCATGAAGGAATTACGTCAAATGTTGGATGAGCTGGCGGCTGAGACTGGTAGAACTTACGAGTTGACCTCTGCCATCAGCGCCGGTTGGGACAAGATCCAAGTCGTGAACTACAAGGAAGCCCAGAAATACATGGACCATATCTTCGTTATGAGTTACGACTTCAAGGGTGCCTGGTCTAACGACACTCTGGGACACCAGACTGCTCTATATGCTCCATCTTGGAGTCCTAAGGAAACTTACACCACCGACTTCGGTATCAGGTACCTGCTGACCCAAGGTGTCCAGCCCAAAAAGCTGGTTGTCGGTGTTGCTATGTACGGACGTGGTTGGACTGGAGTTCACGACTACAACGACGACATACCCTTCACTGGAGTTGCTAATGGTCCAGTTAAAGGAACTTGGCAGGACGGTGTGGTGGACTATAGGGAGATCGTGAATGGCATCACTTCAGGAACATGGCAGTACTTTTACGATAAAGTTGCTCAAGCTCCATACGTGTGGAATCCTACAACCGGCGATCTGGTGACTTATGATGACGCAAGATCAGTTATTGAGAAGGGGAAATACGTGAGGAACAATAAGCTTGGTGGACTGTTCGCTTGGGAAATAGACGCTGACAATGGCGATATCCTGAACGCTATGAATATGGGCTTAGGAAATTCCGCTTAG

**>Bm_nscaf2986_109**

ATGCGAGCGATATTTGCGACGTTGGCTGTCCTGGCGAGTTGCGCCGCATTAGTTCAGTCGGACAGCAGAGCGCGCATCGTTTGTTACTTCAGTAATTGGGCGGTGTACCGACCCGGCGTTGGACGTTATGGCATCGAAGACATCCCCGTGGATTTGTGTACCCACTTGATTTATTCCTTTATTGGCGTCACCGAGAAATCAAGCGAAGTTCTCATTATCGATCCTGAGTTGGACGTAGATAAGAGTGGTTTCCGTAACTTCACATCCCTCCGCTCCAAACACCCCGATGTCAAGTTCATGGTCGCGGTTGGCGGTTGGGCTGAAGGCGGCTCCAAGTACTCACACATGGTGGCCCAGAAGAGCACTAGGATGTCCTTCATCAGAAGCGTTGTCGACTTCTTGAAGAAATATGACTTCGACGGTTTGGATCTGGACTGGGAGTATCCCGGTGCCGCTGACCGGGGTGGCTCCTTCTCCGATAAAGACAAGTTCTTGTATTTCGTCCAAGAGTTAAAGAGGGCGTTCATCAGAGCCGGTAGAGGATGGGAGCTGACTGCTGCCGTACCGCTTGCTAATTTCAGGCTGATGGAGGGATATCATGTACCAGAGCTGTGTCAGGAACTGGACGCTATCCACGTGATGTCGTATGATCTGCGCGGTAATTGGGCCGGTTTCGCCGATGTGCATTCGCCTTTATACAAACGCCCTCACGACCAATGGGCCTACGAGAAACTTAACGTGAATGATGGTCTTAATTTATGGGAAGAGAAGGGTTGCCCTACCAATAAACTAGTGGTCGGTATTCCGTTCTATGGACGTTCATTCACTTTATCTGCTGGAAACAACAACTATGGCCTTGGAACTTACATCAACAAAGAGGCTGGTGGTGGAGACCCTGCTCCCTATACAAACGCAACTGGATTCTGGGCTTATTATGAAATTTGCACAGAAGTAGATGCAGATGGATCAGGATGGACTAAGAAATGGGACGAGTTCGGGAAATGCCCCTACGCATACAAGGGAACTCAATGGGTGGGCTACGAAGATCCTCGTAGTGTGGAGATCAAGATGAACTGGATCAAGGAGAAGGGCTACCTCGGGGCTATGACATGGGCTATAGATATGGACGATTTTAAGGGACTATGCGGCGAGGAAAATCCCTTGATCAAGCTTCTGCATAAGCATATGAGCACTTACACAGTACCGCCTGCGCGCACTGGACACACAACTCCTACTCCGGAATGGGCGCGTCCACCTTCAACTCCATCAGACCCATCTGAGGGTGATCCGATTCCTACCACAACCACCACCACCGTGAAACCGACGACAACAAGAACCACCGCGAGGCCAACTACTACCACAACGAAAGTACCCCATGGCACCACTGAAGAAGACTTTGACATTAACGTGAGACCGGAAGTCGAGGAACTACCCACGGAAAACGAAGTCGACAATGCGGATGTGTGTAACTCTGAGGACGACTACGTACCAGACAAGAAAGAGTGTAGCAAGTATTGGCGATGTGTGAACGGCGAGGGAGTTCAGTTCTCGTGTCAACCGGGGACAATCTTCAACGTGAAACTTAACGTTTGCGATTGGCCTGAAAATACAGACAGACCGGAATGTTCGTAA

**>Bm_nscaf2829_153**

ATGTGTGAATGTGTTGAACGCGAGCTGTATGCTGTCCGCAGAAGACTCCGGAAGCCATCAAAGGTGTCCACATCGGTTTCGTCCAGCGTCTCTAGATCCACAGATCAAGTTCTCTCAGCTAGCGTGAACAGACCAAAGATTCGTGGTCGTCCTTCAATAGCCAGTAGAAAATCATCCGCGGCTCTGGACAACTCCGTCACCACCGAACATCACAAAGATAAGGATGGATACAAGGTCGGGAAGTTCGTTCCAGAGGACATTCAGCCCGATCTCTGCACCCACATCATCTTCGCTTTCGGCTGGCTGAAGAAAGGAAAACTGAGCAGCTTCGAATCGAACGATGAGACGAAAGACGGGAAGACAGGACTGTACGACAAGATTAATGGGCTGAAGAAAGGGAATCCTAAACTAAAGACTCTATTGGCCATTGAAATATCTTACCAGGGCTATGGAGTCCTCCCCTATACCTTGTATACGATTATACCTCAAAGTATCACAAAACAACCAGCTTTGTAA

**>Bm_nscaf2829_151**

ATGAGTGAAAGAGAGCTCCGTGAGGCCTTCGAAGCGGAGGCTCAAGAAGTGAAGAAACCTCGTTTGCTTCTTACTGCAGCTGTGCCAGTTGGACCTGACAACATCAAGAGCGGATACGACGTTCCTGCTGTAGCCAGCTATCTGGACTTCATCAACCTTATGGCCTACGACTTCCACGGCAAATGGGAGAGGGAGACCGGCCACAACGCCCCGCTGTACGCTCCATCGACAGACTCGGAGTGGAGGAAGCAACTATCAGTAGACCACGCAGCACATCTCTGGGTGAAACTTGGAGCCCCGAAGGACAAACTTATTATTGGTATGCCAACATATGGACGTACATTCACGCTCTCCAACCCTAATAACTTCAAAGTGAACGCGCCGGCCAGTGGTGGAGGAAAAGCGGGAGAGTACACCAAGGAAAGCGGTTTCCTCGCTTACTATGAGGTGTGCGAAATGCTAAGGAACGGGGGTGCCTACGTCTGGGACGATGAAATGAAAGTCCCTTACTGCGTCCACGGGGACCAGTGGGTCGGATTCGACGATGAGAAATCAATCAGGAACAAAATGAGGTGGATCAAAGACAATGGCTTCGGAGGAGCAATGGTGTGGACCGTTGATATGGACGACTTCTCCGGCAACGTCTGCGGCGGCGATGTAAAGTATCCATTGATCGGTGCAATGAGGGAAGAACTCCGAGGTATATCTCGAGGTAAAGACGCCAAAGATGTCGACTGGGCCACGGTTGCTTCCAGCATAGTGATAGAAGAAACTGAGAAACCAGAACCGATACAGGTTCCATTGTCCGAAGTCCTCAAGCGCATCAAGAAGCCTGGTAAAAACGTCATCATAAAGACCAATGGAGCGGCACTTTCAGATAAAAACAAACGCGAGCCCCAAGTACTCTGTTATCTCACATCCTGGTCAGCCAAACGTCCAAGCGCAGGCCGCTTCACACCAGAAAACGTTGACCCCAAACTCTGTACCCACATCATATACGCTTTCGCAACCCTCAAAGACCACAAGCTAGCTGAAGCTGACGACAAAGACGCTGATATGTACGATAAAGTCGTTGCGTTGAGAGAGAAGAACCCCAATTTGAAGATATTGCTCGCCATCGGAGGCTGGGCCTTCGGATCTACTCCGTTCAAAGAATTGACTTCGAACGTGTTCCGCATGAACCAGTTCGTGTATGAAGCCATCGAGTTCCTCCGCGACTATCAGTTCAATGGCTTGGACGTGGATTGGGAATATCCTAGAGGAGCAGATGATCGTGCAGCATTCGTATCTCTGCTCAAGGAATTGCGTCTGGCGTTCGAGGGTGAAGCCAAGACTTCAGGTCAGCCCCGGCTCCTGCTCACTGCTGCTGTACCGGCGTCGTTTGAGGCCATCGCCGCCGGATATGATGTACCGGAAATATCGAAATATTTGGACTTCATAAACGTGATGACGTATGACTTCCACGGCCAGTGGGAGCGCCAGGTCGGCCACAACAGTCCGCTGTTCCCTCTAGAAAGCGCCACGAGCTACCAGAAGAAACTAACTGTGGACTACTCCGCCCGCGAGTGGGTCCGCCAGGGCGCCCCCAAAGAGAAGCTGATGATCGGCATGCCCACGTACGGAAGGTCCTTCACGCTTATCAACGAAACACAGTACGACATCGGCGCTCCGGCTTCAGAGGGCGGAGAAGCCGGACGTTTCACCAACGAAGCCGGCTTCATGTCCTACTACGAAATCTGCGAGTTCCTACGAGAGGACAACACGACGCTCGTCTGGGACAACGAGCAGATGGTGCCATTCGCTTACCGAGGCGACCAGTGGATGGCCTGGCTGAAGGAAGAAGGGTTCGGCGGGATCATGGTCTGGTCTGTCGACATGGACGACTTCCGCGGCTCCTGTGGAACTGGCAAGTTCCCGCTCATCACGACCATGAAGCAGGAACTGTCCGACTACAAGGTCAAGCTGGAATACGACGGGCCCTACGAGACGGTGCTCACCAGCGGACAGTACACCACTAAGGACCCCACTGAGGTGACGTGCGAGGAGGAGGACGGCCACATCTCGTACCACAAGGACCAGGCGGACTGCACCATGTACTACATGTGCGAGGGCGAGCGGAAGCACCACATGCCGTGCCCCTCCAACCTCGTGTTCAACCCCAACGAGAACGTCTGCGATTGGCCGGAGAACGTGGAGGGATGCGCGCACCACACGCAGGCGCCGCCCGCCAGACGATAG

**>Bm_nscaf2993_229**

ATGAATTCAATCTCTCCAACAGCACCGGCGCCTATAACAGAAGTACCGCTCGATTTAGCTACAAAGTGCGAGCCTGCGGAATGCCAATTACCGTACTGCTTCTGTTCCAAAGATGGTACATTGATTCCTGGCGGCCTCGACCCTGCAGAAACACCTCAAATGATAATGTTGACTTTCGATGGAGCCGTAAATTTGAACAACTTCGACAAGTACAAGAAAGTCTTCAAAGGAAAGATTAACAATCCAAACGGATGTCCTATAAAGGGGACATTCTTCTTGTCTCATGAATACAGTAACTACGTGATGGTACAAGCTCTCGCACACGATGGACATGAGATAGCAACAGGTACAGTTTCGCAACAGCAAGGCTTGCAAGACAAAGGGTACGAGGAATGGGCCGGTGAGATGATCGGCATGCGAGAGATCCTTAAGAAGTTTGCGAACATATCGCGGTCGGAGATCGTGGGAGCCCGAGCACCGTTTCTGAAGCCTGGCAGGAATACGCAGTTCAAAGTGTTAGAAGACTTCGGATACATCTACGACAGTTCAGTGGGCGTGCCTCCTTTGCCGATTCCGGTGTGGCCTTACACGCTCGACTACAAGATCGCCCACGAGTGCAAGTCCGGAACATGCCCCACTAAATCGTTCCCGGGACTTTGGGAGATACCATTCAATGCTCACTACGTGGAATCATTCGAGGGAGGTCATTGTCCTTACTTGGACCAATGCGTTCTACATAATCACGATGCTAACGAGGTAAGTGAATAA

**>Bm_nscaf2993_230**

ATGCCGCTTCACACCAATTGGTTCCAAATCAAAGAACTTGAGCTTGGTCTACATAAGTTCTTGAAATGGGCGGCAGATCTGGAAGACGTATGGTTTGTAACAATGACACAAAGTTTAACATGGATGACGGATCCACGACCAGTGAAGGCGCTCAACAACTACGAAGCGTGGCGTTGTGACAACAAAGAGCTGCCTTCCGCTCCCTGCAACTTGCCTAATAAGTGTGCTCTTTCGTTCAAACAACCGGATAGCAATTTCACTGATACAAGGTACATGGAGACTTGCAGCGAGTGCCCTAACCAGTGA

**>Bm_nscaf3031_201**

ATGGTAAAAATATATGATCTGCTCAACGTGCCTGCAGAAACTAGGGAGCTATTAGTGGAAATGACGCAAGTGATAAGCAATAACTTGTTGGCTGAGTGCGGAGGACACGTCACTGAGGTGGTGGACACTCAGGTCGTTGTCATCATCGTGGTGAAGACCGCCATCACTTCGTTGAATTGGAACACTGACGAACAATACATGCTGGACGTACAAACTAGAGGAGGAGAAGTCTCTGTCCATATAGAAGCAGAAACCATTTACGGAGCTCGTCACGGCTTAGAGACCTTCTCTCAATTAATATCATCAGACAAACGTGACTTTTCTGATGTGGAACATTGCGGTTTGGTCCTCGTGTCCGGTGCAAAGATTCGTGACAGACCGATCTACAAACATAGAGGCTTAGTTCTAGATACTTCTAGACATTTCATACCGATGGTCGACATTAAAAGAACTATCGATGGTATGGCGACAACAAAAATGAATGTCTTTCATTGGCATGCCACCGATTCTCACAGTTTTCCTTTGGAAGCGAGTCGGGTGCCTCAGTTTACAAGATATGGCGCCTACTCGGGCAGCGAAATGTACACGACGGAAGAAATACGAGAGTTGATACATTACGCGAAGGTACGCGGCATCCGAGTCGTCATTGAAATAGACGCGCCGGCGCATTCAGGAAACGGATGGCAATGGGGAAGGGAATACGGCTTAGGTGATTTAGCGGTATGCGTTAACGCTTACCCCTGGAGACATCTTTGCATCGAACCTCCTTGTGGTCAACTGAATCCAGCGAACCCTAATATGTACCGGGTGCTGAGGAACTTGTACCAAGATGTTGCGGATCTGTTGAATTCACCGCCATTGCTACACATGGGAGGAGACGAGGTTTACTTCGGGTGCTGGAACTCGAGCCAAGAAATTATCTCGTACATGAAGGACCAAGGATACGATACGACGGAAGAAGGTTTCATGAAGTTGTGGGGTGAATTCCATAACAAAGCCCTTCAAATTTGGGACGAAGAGATTTCCGCGAAAGGACTGGACCCACAGCCCGTGATGTTATGGTCTTCGCAACTGACACAGGCTCAGAGGATCTCACAACACTTGGACAAAGAAAGATACATCATTGAAGTTTGGGAGCCTTTGAACAGCCCATTATTGACTCAACTCTTGAGGCTCGGCTATAGAACTGTTTCAGTACCGAAGGACATTTGGTATTTGGATCATGGCTTCTGGGGCAGAACGGTATATTCCAATTGGAGAAGGATGTATGCTCACACTCTACCACGGGACGAAGGCGTGTTAGGCGGTGAAGTGGCCATGTGGACGGAGTATTGCGACGCTCAGGCCCTAGGTAAGTTGGTTACTAGACAATGTAGCTATTTAGAGGGTAGACACCCGAGTATGGCCGAGAGCGGCTGCAGTGGCGGAGAGGCTGTGGTCAGACCCGACGTCCACAGTGTACAGCGCAGAGCCGAGGCTGCAGAGGCTCCGGACGAGACTGATAGCTCGGGGCCTGAGGCCCGACGCCATGTCCCCGGCGTGGTGCTCCCAGCACGACAGCAAATGTCTTTGATTAGAAAAAAAAATGGTGCACGTGCAACTTGGTACACGTGA

**>Bm_nscaf463_07**

ATGGCCATGAACAAGATGAACGTATTTCACTGGCACATTGTTGACGACCAGAGTTTCCCATATCAGAGCGAAAGGTTTCCCGATTTGAGTCGTCTGGGAGCCTATCACGAGACCTTGATTTACACCAAGAAAGACATTCAGACGGTTATCGACTACGCAAGGAACAGGGGGATCAGAGTTATTCCTGAATTCGATGTACCCGGTCACACAAGGTCCTGGGGTGTAGCCAAACCCGAACTCCTGACACATTGCTACAACGAATACGCTGTCGACATGGGTCTCGGTCCGATGAACCCAATCAAGGACAGCACATACACCTTCCTGCGGGAACTGTTTCATGAGGTCCAGGCCTTGTTTCCGGATAGATACATTCACATCGGAGGTGATGAAGTCGATCTAGATTGCTGGGAGTCGAATCCAGAATTCCAGAGATACATACAAGAGCACAACCTGACTTCGGTAGCTGATTTTCACGCTCTATTCATGCGCAACACGATTCCTCTGCTCAGCGAAAACTCGAGACCGATTGTGTGGCAGGAAGTTTTCGATGAAGGCGTGCCCCTTCCGAAGGACACTATCGTGCAAGTGTGGAAGGGAAACGAAGTTTACGAAATGTTGAATATCCTAAGAGCAAGTCACCAGTTAATCTATTCCTCTGGCTGGTACCTTGACCATCTGAAAACTGGAGGCGATTGGACGGAATTTTTCAACAAGGACCCTCGGGATATGGTCAGCGGCCTCAGCAAAGACATCAATGTTGACAACATTGTAGGTGGCGAAGCTTGCATGTGGACAGAAGTTGTTAACGACATGAACATTATGAGCAGAGTATGGCCACGAGCTAGTGCTGTAGCTGAAAGACTGTGGGGCCACGAATCACAGGCAGCATATCAAGTATACTCTCGCTTAGAGGAGCACACATGCAGAATGAACGCGCGCGGCATACGCGCCCAGCCACCCAGTGGCCCTGGCTTCTGCCTAGGCGCTTAG

**>Bm_nscaf463_08**

ATGGCCGTTCAATACCCAGTAAAACTATGGAGGGTAGAGCAATTGCACATTAACAAAGGTGAAGTTCACGACTTTCCCCGTTACGCTCACAGAGGACTGCTCGTTGACACATCACGTCATTACATCTCAATGTCTAATATCCTACTCATTTTGGACGCTATGGCCATGAACAAGATGAACGTTTTTCACTGGCACATTGTTGACGACCAGAGCTTCCCATATCAGAGCGAAAGGTTTCCGGATTTAAGACAGTCTATAAGAATCGTTGGTCTAGGTCACACAAGGTCCTGGGGTGTAGCCAAACCCGACCTCCTGACACATTGCTACGACCAAGACGGTGACTACGTGGGTCTCGGTCCGATGAACCCAATCAAGGACAGCACATACACCTTCCTGCAGGAACTGTTTCATGAGGTCCAGGCCTTGTTTCCGGATAGATACATTCACATCGGAGGTGATGAAGTCGATCTAGATTGCTGGGAGTCGAATCCAGAATTCCAGAGATACATACAAGAGCACAACCTGACTTCGGTAGCTGATTTTCACGCTCTATTCATGCGCAACACGATTCCTCTGCTCAGCGAAAACTCGAGACCGATAGTGTGGCAGGAAGTTTTCGATGAAGGCGTGCCCCTTCCGAAGGACACTATCGTGCAAGTGTGGAAGGAAAACGAAGCTCCTGAAATGTTGAATATCCTAAGAGCAAGTCACCAGTTAATCTATTCCACTGGCTGGTACCTTGATCATCTGAACACTGGAGGCGACTGGACGGAATTTTTCAACAAGGACCCTCGGGACCTGGTCAACGGCCTCAGCAAAGACATCAATGTTGACAACATTGTAGGTGGCGAAGCTTGCATGTGGGCAGAAGTTGTTAACGACATGAACATTATGAGCAGAGTATGGCCACGAGCTAGTGCTGTAGCTGAAAGACTGTGGGGCCACGAATCACAGGCAACATATCAAGTACACTGTCGCTTAGAGGAGCACACGTGCAGAATGAACGCGCGCGGCATACATGCCCAGCCACCCAGCGGCCCTGGCTTCTGCCTAGGCGTTTAG
