# Supplementary material for: Transcriptomic analysis of developmental features of Bombyx mori wing disc during metamorphosis
Source: BMC Genomics. 2014 Sep 27;15(1):820. doi: 10.1186/1471-2164-15-820 (PMC4196006; doi:10.1186/1471-2164-15-820)
Supplement: Supplementary file 8 — Additional file 8: Assembled nucleotide sequences of transcripts in Table 6. (DOC 44 KB) [file 12864_2014_6525_MOESM8_ESM.doc]

**>Bm_nscaf2800_23**

ATGTTTATGGTGTGGCTTTGGGTCGGTTTTGCGATGGCCGCTAGTGTCGTTGCTGATCGTAGCAAACTACCTCCTGCCTGTGATAGTATGATTTACTGCCACGGACCGTTGTTAAACACGGTCCAAATGGCAGGCCTCTACAATGACTCCAAGACGTTTGTGGATATGAAAATTAAAATGTCACCAAAGATCACCTTGGAGCACTTCTATGACATGATGAGCAGGACAGATTCTAATCCGACTAAAGCGGACATACAGGAGTTCGTGAATCAGAACTTCGATCCTGAGGGTTCGGAATTTGAAGACTGGAGGCCCAGTGATTGGAAGCATAACCCTGGATTTCTTGCTAAAATCAAGGATCCGTTATTGCACAAATGGGCATCAGCCTTGAACGATTTATGGCTCGATCTCGGGAGGAAGATGAAGGAAGCAGTCAAAGAGAGTCCAGACTTATACTCCATTATATACGTGGAGCATCCTTTTATTGTTCCAGGTGGTCGTTTCCGTGAGTTCTACTACTGGGACTCGTACTGGATCGTCAAAGGTCTGCTGCTCTCGGAGATGAGGACCACAGCGAAGGGCATGGTCAACAACTTACTCAGCATAGTCGATAGATACGGTTTCATACCGAATGGCGGCAGAATTTATTATCTTATGAGATCTCAACCTCCCCTTCTAATACCGATGGTGCAGCTACTAATGGAAGACACCGATGACCTGGAATACCTGAGGGAGCACATCCACACCCTGGACAAAGAGTTCGACTACTGGATGACGAATCACACCATCGAAATTAACCACGAAGGGAAGAAATTGAAAATGGCGATGTACATGGATAATTCTCAGGGTCCGAGGCCGGAGAGCTACAAAGAAGACGTCGACTGTGCTAGACATTTTGACACAACTGAGAAGAAAGAAGAATTGTACGCTGAATTGAAGGCTGCAGCTGAATCCGGCTGGGATTTCTCATCAAGATGGTTTATACTGAACGGAACCAATAAAGGTAATCTAACTAATTTGAAGGTGAGGTCGATAATTCCCGTCGACCTCAACGCGATCTTGTGCTGGAACGCGCAGCTCATGATGGAGTACCACACCCGCCTTCAAAATGAGGAGAAAGCATCTTACTATAGGCGGATACACGATGACTTCATGGAAGCTATCGAAGAGCTATTGTGGCACGAAGACGTGGGAGCTTGGTTGGACTACAGCCTCGAGTCTGGTCGCAGGAGAGACTACTTTTACCCATCCAACCTGTCTCCGCTCTGGGCCGGCAGCTTCGATAAAGCGAGAAAGGATTATTTCGTGAATAGGGTCATCAATTATTTGGACAAAGTTAAGATGGACATCTTCGAAGGCGGAATCCCAACAACGTTCGAACATACCGGCGAACAGTGGGACTATCCCAACGCGTGGCCGCCCCTCCAGTACATCGTCATAATGGGACTAGCCAACACCGGGCACCCGGAGGCGATGAGATACGCCACCGAACTCGCCACTAAGTGGGTGCGGAGTAACTTCGAAGTCTGGAAGCAGAAGGCCGCCATGCTGGAGAAGGAAGAAAAATTTGGTAGGGAAATAAAAGCGGGATACTTCAGCTACGAGTCAGATGTGGGAGTGTCTCCAGAAGACTTTAGTTCCACTATACCACGAGATAAACTCGTAGCAAACTGGAATGAGGAAGAATAA

**>Bm_nscaf2800_24**

ATGGGTGTAGGTAAACCTCAAAACTACATATTAATGGTGTACAAGAGACGTAAAGATTACGCCGGCTTCTGTAACGGCGAAGTCGACAAACTCATCTCGAAAAAACCCTACACTGAACTCAAGAGTTTGAACGGCGCGTCGAACCAGAAACAGCGGTGA

**>Bm_nscaf2829_133**

ATGAGGTTGTTTCTACTACTGGTGGGATTGACGACGGTAATCGCGGATGACCTACCGCCGACTTGCATCAGACCAGTTTACTGCAACAGTACGCTGCTTCATTACGTTCAAATGGCCAGACTCTACCCCGATTCTAAAACTTTCGTCGATTTTCAAATGCGTAAAGACGAGAACGCAACACTGTCCGCCTTCCAAGAGCTGTTGGACCGCACGAATCATAATCCAACGAAAGAAGACCTGCAGGAATTTGTCGTTGACTTTTTTGATGAAACCAGCGAACTTGAAGAATGGAAACCGGATGACCATAAAGAAAACCCGCCTTTCCTCGCCAAAATTCGTGATGAAGGTTTCCGGGAATTCGCTAAAGCTTTAAATGATATTTGGCCAACTTTAGCCAGACGCGTTAAGCCCTCGGTCCTTGAAAAACCCGAACAATCGAGTCTCGTGCCTATGACACATGGCTTCATCGTTCCTGGAGGTCGATTTAAAGAAATCTACTATTGGGACGCATATTGGATTATCGAAGGTCTTCTAATCACTGACATGACCGAAACTGCAAAAGGAATGATCGAAAACCTAATCGAGCTTCTGTATAAATTTGGCCACATTCCCAATGGAAGCAGATGGTATTACCAAGAACGTAGCCAGCCTCCTTTATTGGCGGCCATGATAAAGCTTTATTACGAAAAGACTAAGGACATCGAATTCATCAGAAAGTATATTAGTGCTTTAGAAAAGGAATTAGAATATTGGCTGGACACTCATCTTATCGCTTTCAACAAGAACGACAGAGTTTACACTCTCCTGAGGTATTATATTCCAAGCGCTGGTCCTCGACCGGAGTCCTATTACGAAGATTACGAATTGGCTCAGAAATTAAACAAGACTACTGATCCTAATGATATTTACGCCGATTTAAAAAGCGCCGCTGAGAGCGGGTGGGACTTTTCTACGCGCTGGTTTATTTCAGAAAGCGGCGACAACAGTGGTAATCTAACCAATTTAAACACAAAGAACGTTATCCCCGTAGACTTGAATGCTATTTTCGCCGGAGCGTTACAGATTACGGCGAACTTCCAAGCTATATTAAAGAATCCTCGGAGAGCGGCTCACTGGGGCTACATGGCCGAACAATGGAGAAGTTCTATAGAGCAAGCGTTATGGGATGAAGAAGACGGAGTCTGGCACGACTACGACATATTGAATAATAAGCCACGTAGATATTTTTACACTAGCAACCTAGCTCCCCTGTGGATGAACGCAGTCGAAAAACCATTTCTAGCTAAGCATGGCGCTAGAGTCCTGGAGTACCTTCACGAATCGCAAGCGCTCGAGTACCCCGGAGGTGTCCCGGTGTCTCTAGTCAACAGTGGAGAACAATGGGACTTCCCTAACGCTTGGCCTCCAGAAGTCAGCATTGTCGTTACGGCTATCCAAAACATTGGTTCCGAGGAGAGTAGTAAATTGGCAAAAGAACTCGCCCAGGTCTGGGTGAGAGCTTGTAAATCAGGATTTACCGAAAAGAAACAGATGTTTGAGAAGTACGATGCGTTGAACGCTGGTAAATACGGCGGCGGCGGTGAATACACGGTTCAAGATGGATTCGGATGGTCGAACGGCGTTGTCCTGGAGTTCTTAGATAGTTACGGAGCTGTACTGACTTCGGTGGACTCTGTAGCTGCTAGTGCAAATAACGGTCAGTCAAACGAAGAATCTGAGACGGATTCTAAGGAAAAATAA

**>Bm_nscaf2829_134**

ATGTATAAATCACGTTCTATACGCCTGTACCGCGTGATCGGGCTGCTTGGAGCATGCGCTCAAGCTGTGCACATTGTTCCCGCTTGTAACTCCTCAATACATTGCTCCGGTGAACTCCTGCATAAAGTTCAGCTGGCCCGAATCTTTCCCGACTCGAAGACTTTTGTCGACTTGAAACTAAAGTATCCGGAATATCAAACTCTCGCAGATTTCGCTGAATTGATGAAAGAGACACATCATGAACCCAGTCGCGCTAGTCTAGCTCGATTTGTGGACAGTCATTTTATGGAAGGGAATGAACTCGAAGATTGGGATCCACCTGATTTTGATCCCGAGCCGCCAATCCTGGAAAACATCACAGATGTCAGACTCAAGCGATTCGCTAAAGGCATTATTAGCATTTGGGCCAAATTAGGCCGAAAAGTTAGGCCAGATCTCATTCAAGAAGAAGACCAGTATAGTTTCATTTCGGTGCCCAACGGTTTTATTGTTCCTGGTGGAAGATTCAAAGAGCTATATTACTGGGATTCGTTTTGGATGATACGTGGATTAATTATTAGTAACATGATGCAGACAGCAAAAGGGATGATAGAAAATTTGTTACATTTGGTGGAAAAAGTTGGCTATATACCTAATGGCAGCCGAATTTATTACCTAGGCCGCAGCCAACCCCCCCTACTCACAGCAATGGTCGCAAGTTATTTTGCAGCCACCGGCGACATCGTGTGGCTTGAACGACACATCAGCACCGTCGAAAAAGAACTTCAATATTGGCTCGACAAAAAGAAAGTCACTGTTACAGTTGAAGGTAAAAAATATATTCTTCTCCGCTATCTTTCTGACAGAAGAAGTAAAGGACCTCGTCCAGAATCTTATTATGAAGACTATACTAACGCACAAGTGTTACCCACTGAAGAGTTGCGAGAAGATTTCTACAATGAAATGAAAAGTGCAGCAGAAAGTGGATGGGACTTTTCGACTCGATGGTTTGTTACTGCCAAGAATGAAACTGTTGGCTGCTTGACTGACGTTCACGCGACTCGTATCGTGCCTGTCGATCTAAATGCTATCTTCGCTGGGGCCCTTGAACTTGCCGGTGACTTCAGGTATCATCTAAAAGACAGGCGTGAAGCGAAAAAATGGTGGAGTCTTGCCAAATATTGGAGGAACGCGATTGACGCCGTGCTTTGGGATGCCAATGATGGTGTTTGGTACGATTACGACACTCAAGCTAAAGCTTCGAGACGTCACTTTTATCCAAGCTCCGCGACTCCGTTATGGTCAGGAGCCGTGGAAAGCTCCGAAGCTCCACGGTATGCGGCGAAATTCGTAAAATATCTCCTGTCATCTGGCGCGTTAAGCTTCCCTGGTGGGATCCCAGCTTCCGTGCTACATTCAGGTGAACAGTGGGACTATCCAAATGCTTGGCCTCCTTTACAGAGCATCTTGATCGGTGGCTTAGAGATGAGCGGAAATGAAGAGGCAAGGAGGTTGGCGAGAGAACAAGCAAGAATATGGATTCGCGCCAATTATATCGGTTTTACTACATGGAATAAGATGTTTGAGAAGTACAGTGTAGTAGAACCAGGGCATCACGGCAGCGGAGGGGAGTATATAGTGCAGGATGGTTTCGGGTGGACCAATGGGGTCGCCCTAGAACTCTTGCAAATGTACGGGAAGGAGATGACGTTGGATGACAGCCCCGAAAATTTTCCGTACCTAACGGTGGTAAAATAA

**>Bm_nscaf3027_062**

ATGGCGGCGGTGCGCTACGTGTGCGAGTGCGTGTCGCGGCGGGCGGCGCACCTCGTGTCGGCAGGCATCGCGACGCTCCTCAACAAGATGAACGAGCCGAGGGTCACCGTGGGAATCGACGGCTCCGTATACCGCTTCCATCCGCACTTCCACACGCTAATGTGTGAGAAGATCGCTCAACTTGTCCGACCCGGACTACAGGTAATTGTCTGA

**>Bm_nscaf3027_063**

ATGACCGACAAACAAATCAAAGAAATAATGTGCAGGCTTCATAACGATTTGAAGAAAGGGCTTAGCCGCGCTGATCATGACAAAGCTACAGTAAAGTGCTGGATCACATACATACAAGATTTACCCAACGGAAAAGAGAGAGGTAAATTTTTGGCATTAGATTTAGGTGGAACGAATTTCAGAGTTTTAATAATAAATTTAGGGGAAAACCATTTTGATATGCAGTCCAAAATCTATGCCATACCAAAACATATAATGACTGGCACCGGCATTGCTCTATTTGACCACATAGCCGAATGTTTACCAAATTTTATGAAGGAGCACGGAGTTTATGAAGAAAGATTGGCACTTGGATTTACATTCAGTTTTCCACTAAAACAACTGGGTCTTACAAAAGGCGTATTACAAAGATGGACTAAAGGTTTCTCTTGTTCCGGTGTGGTCGGCGAAGATGTTGTACAGGGCCTTAAAGATGCTATCGCCAGAAGAGGGGATGTACAGATTGACATATGCGCTATTCTGAATGATACAACCGGTACATTGATGTCCTGCGCATGGAAAAACCACAACTGCAAAATAGGACTCATCGTTGGTACTGGCAGCAACGCTTGTTACGTCGAGAAAACAGCGAATTGTGAGCTGTTCGATGGCGAACCTGACAAAGAGGAACTGCTCATTAATACCGAGTGGGGGGCGTTTGGGGATGACGGCAGCCTCGACTTCGTGCGGACCGAGTTCGATAGAGAAGTAGACGTCACATCCATAAACCCTGGCAAACAAATTCAAGAGAAGATGATTTCTGGCATGTACCTGGGTGAATTGGTGCGGCTGGCGCTCGTCAAATTCACTCGAATGGGCTTGCTGTTCGGGGGTCAAGGTTCGGATCTTCTGTTCGAAGGGGGCAGCTTCTATACCAAATACGTGTCTGAAATTGAATCCGATAAACCCGGAGACTTCACCAGCTGCATGGAGGTCTTGGAAGAACTCGGTAATATATTTATCTAA

**>Bm_nscaf2780_10**

ATGCTCCAACTTTTCCAACAAGATCGGGAGCGTTTCGAAAAGTTCAGCCTCTGTATCCCAACACCGAACGATGGAGACATCCTACTTGACTATTCCAAGAACCGTATCAACAGTGATGTTTTTAAGTTGCTCCTCGATCTCGCTAAGAGCAGAAATGTTGAACAAGCCAGAGATGCCATGTTCGCAGGTCAAAAGATAAACTTCACAGAAGACAGGGCAGTACTGCATATAGCTCTGCGTAACCGTCAGAACAAGCCTATCCTGGTGAACGGTAAGGATGTCAGCACTGATGTCAATGCGGTACTAGAACACATGAAGGAATTCTCCGATCAAGTAGTAAGCGGGCAATGGAAGGGGTATACGGGAAAAGCTATAACAGACGTGATCAACATTGGTATTGGAGGCTCAGATGTGGGTCCTCTCATGGTCACAGAAGCACTGAAGCCCTATGCTAATCATCTTAAGGTCCATTTCGTATCTAACATCGACGGCACCCACCTGGCTGAGGTCTTGAAGAAGCTGAACCCTGAGACAGCGCTGTTCATCATAGCATCCAAGACTTTCACCACACAAGAGACGATAACCAATGCGAGCTCAGCTAAGACGTGGTTCCTGGAATCCGCTAAGGATCCCTCGGCGGTGGCGAGACACTTCGTCGCTTTATCGACGAACGCCGAGAAGGTCACCGCGTTCGGTATCGACGCTAACAACATGTTCGGCTTCTGGGACTGGGTCGGGGGCAGATATTCGCTGTGGTCCGCGATCGGTCTGTCCATCTCGCTGTACATCGGACACGAAAACTTCGAGAAACTTCTGGACGGAGCACATTTCATGGACCAGCACTTCGTCACCGCACCGCTGGAGAAGAATGCTCCCGTGATCTTAGCTCTGTTAGGAGTCTGGTACCACAATTTCTACGGAGCAGAGACCCATGCGCTGCTGCCTTACGATCAATATCTACACAGATTCGCAGCGTATTTCCAGCAGGGCGATATGGAGAGCAATGGCAAGTACGTGACGCGTTCAGGAGCCGAAGTGGAATACTCCACGGGGCCCATCGTGTGGGGCGAGCCCGGGACCAACGGACAGCACGCCTTCTACCAGCTCATACACCAGGGAACCAGATTGATTCCATGCGATTTCATCGCTCCAGCCCAAACTCACAATCCAATTTCGAATGGTGTTCACCACAAGATCCTTCTAGCTAATTTCTTGGCTCAAACCGAAGCCCTGATGAAGGGCAAGACAGCTGACGAGGCTAAAGCTGAGCTAGAGAAATCTGGTATGGCTCCGGAGGCAATAGACAAGATCCTTCCGCACAAGGTATTCAAAGGAAACCGGCCTACAAACTCTATTGTTCTGAAGAAGATCACTCCATTCACTTTAGGAGCACTTATTGCCATGTACGAACACAAGATCTTCACGCAAGGTGTGATCTGGGATATCAATTCATACGATCAATGGGGAGTTGAACTCGGCAAGCAATTGGCCAAGGCAATCGAGCCTGAGCTGCAGGGGACTGCAGCTGTGACCGGCCACGACGCTTCCACGAATGGACTCATTAACTTCCTCAAGAAGAACTTTTGA

**>Bm_nscaf2887_059**

ATGTGTGGAATATTTGCGTACATTAACCATTTAACACCTAAGACCCGGCGTGAAATTTTAGAACTACTTGTCAATGGACTTAAACGTTTAGAATACCGCGGTTATGATTCAGCCGGTGTAGCTGTGGATTCCGCGGATCAGAAAGACATAGCTGTTGTTAAGCGAAGCGGTAAGGTAGCCGCATTAGAAGAATTGCTGCAGGAACGTAGTATTGAACTATCAGTTGAAGAATGTGTGGACTCTCATTGTGGAATAGCTCACACTCGTTGGGCAACTCATGGGGAACCAAGTGCTGTTAATTCTCATCCACAACGGTCTGGCGAGGACAATGCTTTTGTTGTTATTCATAACGGTATCATTACAAACTACAAGGAAGTCAAGACTTTTCTTGAGAACAAAGGCTACATATTTGAATCACAAACAGATACTGAAGCAATTGCTAAGCTGATTCATCATATTTATAATCAGCACAAAGAAAGCAGCTTCCAAGAATTAGTTGAACAGGTAATTCAACAACTTGAAGGAGCTTTTGCTTTATGTTTTAAGTCACGCTACTTTCCTAATGAGTGTGTTGCGACCCGCCGTGGAAGTCCCTTGCTTGTTGGAATTAAGACACCTCGTAGGTTGTCCAGTAACCATGTTCCAATTATGTACACCAACAATAAAGCTACTAGAGGTGTAGTGCCTCCTGTTCCTAGAGTCAATAGTCATGCTCACTTTGAGCCAGAGGAAGAAAGAGATGTTGAATATTTCTTTGCTTCTGATGCATCTGCTGTTATTGAACATACAAATAGAGTACTTTACTTTGAAGATGATGATGTCGCTCATATCAAGGATGGTGTCCTTAGTATTCACCGAATGTCCGGTAGCAGCAATGATCCCCATGAAAGAGAAATTTTTACTTTGAAGTTGGAACTTCAACAGATTATGAAGGGAAATTATGAATATTTCATGCAAAAAGAAATATTTGAACAGCCAGAGTCCATTGTAAATACAATGAGGGGCAGATTAAACTTTGCAAATGGTACTGTTACTTTAGGAGGTATTAAAGATTATATTCCTGAAATTAAGCGCTGTCGAAGACTTATGTTAATAGGATGTGGTACAAGTTACCACAGTGCTGTAGCTACCCGACAGCTTTTGGAAGAATTAACGGAGCTACCAGTTATGGTGGAGTTAGCTTCTGATTTTCTAGATAGAAACACCCCTGTTTTCAGAGATGATGTATGTTTCTTTATCTCTCAGTCTGGTGAAACTGCAGACACACTTATGGCTCTCCGATACTGTAAACGCCATGGTGCCCTTATTGTTGGAATAACAAATACTGTTGGAAGTTCAATATGCCGTGAATCACATTGTGGTGTTCATATCAATGCTGGTCCTGAGATTGGTGTTGCTTCTACTAAGGCATACACTTCTCAGTTTGTTTCATTGGTCATGTTTGCTTTAGTGATCAGTGAAGATAGAATTTCACTACAAAAAAGACGTGCTGACATTATAGAAGGTTTACATGAGTTGGATTCAAAAATTCGCCAGGTTCTTGCCTTAGATGAAGAAGTCAAAGCTTTGGCACAAGATCTTTACCGACAAAGATCACTCTTAATAATGGGCCGAGGTTACAACTTTGCTACATGCCTAGAAGGTGCACTCAAAGTTAAAGAACTAACCTACATGCACAGCGAAGGAATTATGGCAGGTGAATTGAAACATGGGCCTCTAGCACTCATAGATGATTCAATGCCTGTTATGATGATAGTGATGCGTGACCCAGTTTACATAAAATGCATGAATGCTCTACAACAAGTAACAGCACGTCAAGGTCGTCCAATAGTAGTCTGTGAGGAGGGTGACACAGAAACAATGGCACTCGCATCACAAAGTCTCCAAGTGCCTAAAACAGTTGATTGTTTACAAGGTGTCCTAACAGTTATTCCTATGCAGCTTCTGGCCTACCACATTGCTGTTTTGCGTGATTGCAATGTTGACTGCCCGCGCAATCTCGCAAAATCCGTTACAGTGGAATAA

**>Bm_nscaf2823_132**

ATGGGCATGGAAAAGAACGGAAATGCGGAAGGGAAACTCTCCGAATACCTTTACCCTCCGGAGATATTAAAAAGACTGGATTTCAGTAAGAGTCCGGCAAAGTTTCATCCTAAAATCAGTGCTGTTGACCCCGGCGAGGATTGGATGATTGTGAGGCCTTTACAACGCTCTGATTACGATAAGGGTTTCCTGCAGCTCCTCAGTCAACTAACAAGCGTGGGAAATATTACGAGAAAACAGTATGATGACCGTTTCACTAAGATGAAACACTCGGGCGGTTACTATGTGACCGTAATAGAGGACACGCGCATTAATAAACTCATAGGTGCAGCAACGCTGACCATCGAACAGAAATTCATCCACAACTGCTCTTTGCGCGGCCGTTTAGAAGACGTCGTAGTTAATGACACTTACAGAGGAAAGCAACTTGGTAAATTAATTGTGGTAACCGTATCGCTTCTCGCTCAAGAGCTGGGATGCTATAAAATGTCTCTCGACTGTAAGGATAAATTAATAAAATTTTACGAGACGCTAGGATATAAGATGGAGCCTGGGAATTCTAACGCTATGAACATGAGGAATTCAATTATTATCGCAGACAATAATTGTTTTGAAGACTAA

**>Bm_nscaf3003_158**

ATGCACTTGTTGGACGGCGATCGTATAGCCACATTACTGGCCAGCTACATCACCGAATTGCTGACGGCCAGCGAAGCGAAACATTTGAAACTAGGCCTCGTGCAAACGGCCTACGCTAACGGTGCATCCACTGCTTATATAACGCAGAAGCTGAAAGTCCCGGTGAGCTGCGTGAAGACAGGAGTGAAGCATCTCCATCACGCAGCGCTTTCGTATGACATCGGCGTCTACTTTGAAGCGAACGGGCACGGCACAGTCATATACAGTCACGACGCTAAGAAGACGATCAGCAAAATTGCCGAAGAGGGGGAGTCGGAACAAAGGAAGGCGGCGCAGTTGCTTCTGGACTTCATCGACATGACCAACGAGACGGTCGGAGACGCCATATCGGACCTGTTCCTCGTCGAGACGGTGCTCTGCGCTCGAGGACTGGACGCCGAGCAGTGGCTCTCCACGTACGACGACCTGCCCTGCAGACAAGTAAAAGTAACAGTACAGGATCGTAACGTGATATCTACGGCGGACGCGGAGCGCGTGTGCACGTCGCCCGAGGGACTGCAGACGCGTATCGACGAGCTCGTCGCTGCGTACGCGGACGGACGCGCCTTCGTGCGTCCCTCCGGCACCGAGGACGTGGTGCGAGTGTACGCGGAAGCGGATACGCAGCAGTCAGCAGATAAACTCGCCGCAGAAGTATCACAGGCCGTTTACGATTTGGCCGGCGGCGTTGGAAATCGGCCAGAGTTGCCAGCTTAA

**>Bm_nscaf2176_247**

ATGTATGAAACATTATTACGAAACCTAAAAGATCATGGTCAAGAACACTTAATTAAATACTGGTCGGTATTAAGTGAGGAACAGCGGAAACAGCTGTCGGACGAAATTCTAAAACTTGACTTGACGGAAGTGCATGCAACATTTTCGCGGGCAATTGAATCTACGAAAAAAATATTAGAAAAATTAGACGACGACTTGAAACCAATACCGGATAGTCACTATGAGTCTGTACCGAACTTGACGCCCGATAAGATTGAAGAATACGAAAATATCGGATTTAAAGAAATATGCAATGGGAAAGTTGGTGTGCTCTTGTTAGCTGGAGGTCAGGCGACGCGGCTCGGTTTCGGCCACCCTAAGGGAATGTACGATGTGGGATTGCCATCAAGAAAAACCTTGTTCCAGATACAAGCCGAGAGGATCCTGCGAGTTCAGCAGATGGCTGCCGAAAAATATGGAAACGAAGGTAAAATAACATGGTATATTATGACTTCTGAACACACGAAAGCCCCTACTGCAAATTATTTCAAAAGTCACTCATACTTTGGTTTGAATGAAAACGATGTGGTTTTCTTTGAGCAAGGCACATTACCTTGCTTTGATTTTGAAGGGAAAATATTCTTGGATGAGAAATATCATTTGTCTGCTGCCCCTGATGGTAATGGAGGTCTGTATCGAGCATTAAAAACCCAGGGAATATTGGACGATATCTCAGTAAGAGGAATCCAACATCTTCATGCTCATTCAGTTGACAACATACTTATAAAGGTTGCAGATCCGGTATTTATTGGTTACTGTAAATCTAAAAATGCTGATTGTGCTGCAAAAGTCGTTCAGAAATCTAGTCCCAGTGAGCCTGTTGGTGTCGTCTGCAGAGTGAATGGACACTACAAGGTAGTGGAGTACTCCGAACTCACAGACGAGGCATCTGAAAGACGTAATCCTGATGGTCGTCTCACATTCTCAGCAGGAAACATCTGTAATCATTATTTTTCAGCTGATTTCTTAAGAAAAATATCAAACTTTGAGACAAAGCTAAAATTGCATATAGCCAAAAAGAAAATACCGTATATAGATGAGAATGGTGTGCGGCAGAAACCGAATGAACCAAACGGTATTAAGATGGAGAAATTTATTTTTGATGTTTTTGAGTTTGCTGAGAATTTCATTTGCTTAGAGGTGGCTCGAGACACTGAGTTTTCAGCCTTAAAGAATGCTGACACAGCCAAAAAAGACTGTCCTTCGACTGCCCGAGAAGATTTGCTTCAGCTCCATAAAAAATACATTAGACAAGCTGGTGGAGAGGTGGCAGACGATGCTGATATAGAAATCTCTCCGTTATTGTCCTACGGGGGCGAGAATTTGGACAGCATTGTTAACGGTAAAGTGTTTACCGCTGGTCCATTCCATTTGAAGAGTCCACAGGAATTATCAAGTAATGGTGTTAATGGTAATCATTGA

**>Bm_nscaf2589_266**

ATGTATAGTCAGCGCACAGTACAAGAAACGAAAGGATGGGACGTGTTCAGAGAATTTCCGCCGAAACAGGACAGTGTGTCCATGGAGACTCAGAAATGTTTGGAATTCACAGTGCGAATGTTAAAAGTGGTCGCTTACCTGGTCACCTTCATCGTGGTCCTTGGATCAGGAGTTATCGCAAAGGGGACAATTTTGTTCATGACGTCACAAATTAGGAAAGACAAGCGATTAGAATTCTGCAATAAAAACTTAGGTCGAGATAAGCAGTTTGTGGTGAGTTTACCAGACGAGGAGCGTGTTGCCTGGATGTGGGCTATCCTAGCTGCCTTTGCTATTCCGGAATTAGGAACGATGATCAGGTCTATAAGAATATGCTTCTTCAAAACATCAAAGAAGCCGAGTTTTGTACAATTTTCTGTGGTCTTTATCGCTGAGTCCTTACATACCATAGGAATGGGTTTACTATTCTTTAAAATCCTGCCTGAATTAGATGTAGTCAAAGGAGCTATGATAACGAACTGTCTCTGTATAATTCCCGCGGTGCTCGGTCTGCTGTCAAGAAACTCTCGAGACTCGAAACGATTTGTCAAAGTCATCGTCGATATGGCCGCTATAGTTGCGCAAGTGACCGGTTTTATAGTTTGGCCCCTGTTGGAAAACAAAGCTGTTTTGTGGTTGATCCCGATCTCTGCTTTATGTATTTCGTTAGGTTGGTGGGAAAACTATGTGACACGACAAAGTCCAATAGGAATAATCAAAAGCCTGGGGAGACTAAAAGAGGAATTAAATTTTACTCGCTACTTTACGTATCGATTCATCTCCGTGTGGAAGATTTTACTGTTTCTTTTCTGCAATCTATTCTTCATGTGGTTGGACGGAGATGAACCGGCAATGTTTTTCCAACTTTTCAATCCTGGATTTGGGCCACATAGCATTGTCGTTGAAGAGGTTCAAATCCAACTCGGGGGAACGGTTATTCCAGATTTGGCAAACATAACCCTTACAGGAGACTCCGTCGAAATCGCGGCTGTACATAAGTCCGCCGTCTACGTCATGATGATCCAAATATTTGCAGCCTACATCTGCTACATCTTTGGGAAATTTGCTTGCAAAATTGTTATTCAAGGGTTCAGTTACGCTTTTCCGATAAACTTGGTCATACCTTTGGTGGTGAACTTTTTGATTGCTGCTTGTGGTATCAGAAATGGCGACACCTGTTTCTTCCACGGAACGATACCGGACTACCTATTCTTCGAAAGCCCTCCAGTGTTTACGTTGAGCGATTTCATCTCTCGACAAATGGCATGGGTTTGGTTGTTATGGCTGCTATCACAAACCTGGATCACTATTCATATTTGGACCCCCAAAGCAGAACGTTTAGCTTCCACTGAGAAATTGTTTGTGTTGCCTATGTACAATGGTCTACTCATAGACCAAAGTATGGCACTGAATAGGAAAAGAGATGACCATAAGGATGTCAAAACAGAAGATCTTGCCGAAATCGAAAAGGAAAAAGGTGATGAATACTATGAAACAATATCAGTACATACAGACAATACTGGATCTTCCCCAAAGACTGTGAAATCATCAGACCAGATCACGAGGATTTATGCTTGTGCTACTATGTGGCACGAGACCAAAGATGAGATGATGGAGTTCTTGAAATCTATTCTTCGTCTCGATGAAGATCAGTGTGCTCGCCGCGTCGCTCAGAAGTATCTTCGTGTAGTAGATCCTGACTATTACGAATTCGAAACTCACATTTTCTTGGACGACGCTTTCGAAATTTCGGATCATAGTGACGACGAATCTCAAGTAAACCGGTTTGTCAAGCTTCTGGTAGATACCATCGACGAAGCTGCTTCTAATGTACATCAAACGAACATTCGTATACGTCCACCGAAGAGATATCCTGCGCCATACGGAGGTAGACTGACATGGGTGTTACCAGGAAAGACTAAAATGATCTGTCACTTGAAGGATAAGGCAAAGATTCGACACAGGAAACGTTGGTCTCAGGTGATGTACATGTACTACCTTCTCGGTCATCGTCTAATGGAATTGGCTATTTCGGTTGATCGTAAAGAAGTTATGGCTGAGAATACTTACCTGCTTACACTCGATGGAGATATCGATTTCCAACCTCACGCTGTACGATTACTCATTGATTTAATGAAGAAAAACAAGAACCTCGGAGCTGCCTGCGGGCGTATTCACCCTGTTGGATCTGGACCTATGGTGTGGTATCAATTATTCGAGTATGCTATCGGACATTGGCTGCAAAAGGCCACCGAACACATGATCGGCTGCGTGCTCTGTAGTCCCGGATGCTTCTCGCTGTTCAGAGGAAAGGCTCTGATGGACGACAACGTTATGAAGAAATATACTTTGAAATCTGACGAAGCCCGTCACTACGTACAATACGATCAGGGAGAAGATCGATGGCTATGCACTCTGCTACTTCAACGTGGCTATCGTGTAGAATATTCAGCTGCATCGGACGCCTACACCCATTGCCCTGAAGGTTTTAGCGAGTTCTACAATCAACGACGTAGATGGGTGCCTTCGACCATTGCTAACATTATGGACTTGCTTGTGGATTATAAACATACCATTAAAATCAATGACAATATTTCCTCACCGTACATCGCATACCAGATGATGTTGATGGGCGGTACGATTCTGGGACCTGGAACTATATTTCTTATGTTGGTGGGTGCTTTTGTGGCCGCTTTTAGAATTGATAATTGGACATCCTTCGAATACAATCTATATCCTATAATGTTGTTTATGTTTGTTTGTTTCACAATGAAGTCAGAATATCAAGAAATCGAGCAAGAAAAGAAAGATGCCGAAGAGGCGAAAAAGAAGGCGAAACAAAAGTCCCTTCTTGGTTTCCTGCAGGGTGTCAATTCTAACGAAGAGGAAGGCTCCATAGAACTATCGTTTGCTGGTCTTTTTAAATGTTTACTGTGTACTCATCCTAAAGGAAACGAAGAAAAAGTACAGCTCATGCATATCGCTTCAACACTTGAGAAATTGGAAAAGAAAATTGAAAACGTCGAGAAGGCCGTGGATCCGCACGGTCTATCCAGAAGTCGCAAACTATCGCTAGGCCCTCGTGGTAGCACTAACGGAGACCACCAGTTGGACGCTCTTAATGAGGACCCCGAAGAAGAAAACGATTCCGATTCCGACACCGGCACTCTATCCACTGAACCAAGGGAACGACGAGATGATCTTATAAATCCATATTGGATTGAGGATCCCGGCTTGAAAAAAGGCGAAGTAGATTTCTTGAGCCCGCCCGAGGTACAATTCTGGAAAGACCTTATTGACAAGTATTTATATCCTATTGACGAAGACAAGGACGAGAAGGCTCGCATAGCAGGCGATCTACTAGAACTTCGCAATAAATCAGTTTTCGCATTTGTTATGTTCAATGCTTTATTTATATTGATAGTATTTTTATTACAACTCAATAAAGATCAGCTTCACGTGGTCTGGCCTTTGGGAGTTAAGACAAACATTACGTACATGGAGGAAACAGGCCAGCTTCTCATCTCGAAGGAATACTTGCAATTAGAGCCTATCGGTCTGGTATTTGTGTTCTTCTTTGCCTTGATTTTGGTCATACAATTTTCGGCCATGTTGTTCCATCGATTCGGAACTATTTCGCATATTCTATCATCGACGGATCTCAACTGGTTCTGTTCAAAGAAATCGGAAGATTTGTCACAGGATGCGCTTTTGGATAAGAATGCAATAGCAATAGTGAAAGACCTTCAGAAATTAAACGGTCTGGACGATGATTATGATAACGATTCAGGATCTGGACCACACAACGTCGGCCGAAGGAAGACAATACACAATTTAGAGAAAGCTAGACAGAAGAAACGTAACATAGGAACACTAGATGTGGCTTTCAAGAAACGATTCTTCAATATGAATGCTAATGATGGTCCGGGTACCCCAGTTTTAAATCGTAAAATGACATTGAGAAGGGAGACATTGAAAGCTTTAGAGACCCGTCGTAACTCTGTAATGGCAGAAAGAAGGAAGTCGCAAATGCAGACACTTGGCGCTAATAATGAATACGGTGTTACTGGAATGCTCAATAATAACGTTGGACCTCGTCATAGAGCATCGAATGCTAATATTTCAGTGAAGGATGTATTCTCGGAGCCCAACGGAGGACAGATCAACAGGGCCTACGAAGCCTCACTCGGGGACGACGATGACTCAAATTCCATGCGACTTCAGCCAAGACAAAACCAAGTTTCATTCCAAGGGAGGTTTTAA

**>Bm_nscaf2589_269**

ATGTACTACATCCTCGGTCACCGTTTGATGGACCTGCCGATATCCGTTAACCGCAAGGAGGTTATAGCCGAGAACACGTATCTACTAGCCCTGGATGGCGACATCGATTTCAAGCCGAGCGCAGTGACGTTGCTCATCGACTTGATGAAGAAAGACAAGAACTTGGGAGCCGCGTGCGGCCGCATCCATCCAGTCGGCTCAGGTTTCATGACGTGGTATCAGAAGTTTGAATACGCGATCGGTCACTGGCTTCAAAAGGCCACCGAACACATGATCGGCTGCGTGCTCTGTAGTCCCGGATGCTTCTCGCTGTTCAGAGGAAAGGCTCTGATGGACGACAATGTCATGAAGAAATACACACTGACTTCCCATGAAGCGAGGCATTACGTGCAATACGATCAAGGTGAGGACCGTTGGCTTTGCACACTGCTCCTTCAGCGCGGGTACCGGGTTGAATATTCAGCCGCATCTGACGCGTACACGCATTGTCCAGAGCTTTTCGGAGAATTTTATAACCAGCGACGACGATGGGTTCCCTCGACCCTGGCGAACATCTTTGATTTGCTTGCAGAATATAAGCGCACCGTTAAAGTGAATGATAATATCTCCACACTGTATATCGTTTATCAGACCTTACTTATGATCGGCACTATACTGGGTCCCGGTACCATCTTCCTCATGATGGTCGGTGCAATGAACTCTGTGACCGGTATGAGTAACATGCAAGCTTTAATACTGAACCTAATACCGATAGGCATATACGTTGCGGTCTGTTTGACTTGCAAATCTGAAATACAGCTCATGTTCGCTAATATGATCACATGCGTGTATGCAATGATAATGATGTTAGTGATAATTGGTATAGCGCTACAGATCGTCGAAGATGGTTGGTTAGCGCCATCTAGTATCTTCACGGTTGCAACGTTTGGTCTCTTCTTCGTAACGGCCGCGCTTCATCCCCAAGAGATCAGTTGTTTGATGTACATTTGCATCTACTACATTACAATTCCGAGCATGTATTTGTTGTTGATTGTTTACTCTTTGTGCAATCTCAACAATGTTTCGTGGGGTACTCGAGAAATCGAACAAAAGAAAACTCCTAAGATGTTAGAGATGGAGAAGAAACTTGCGGAGGAAGCAAAGAAGAAAATGGACAGCCAGAATATATTGAAAAAGTTTGGACTTGCAGACGAGACGTCGGGTACATTGGAGTTCGGTATCGCCGGTTTGTTCCGGTGCATGTGCTGCACCAACCCGAAGGATCACAAGGACGACCTGCACTTACTGCAGATCGCCAACTCCATTGAGAAAATTGAAAAGAAATTGTTGGCACTGTATGTAAAGTGA

**>Bm_nscaf2589_270**

ATGCTTTTGGTCCAAGTGCTACTCAGCGATGCCAATGACGGCTTAGACTATACCTTGACCGGCAAGACGTTAGAGCTGCCAGTTTATTGGTCTACATCTCTCTGGGTGGCCGCTATTCAAGTTACAGCAGCGTATTTCTGTTTTGGAGCCGCCAAATTTGCTTGTAAAATTCTTATTCAAAATTTTAGCTTCACATTTGCTCTGACACTCGTCGGACCGGTTGCTATAAATCTCCTTATATTCCTTTGCGGCTTGAGAAACGCGGACCCGTGTGCGTTTTACGGAACAATTCCCGATTATTTGTTCTACGAAATTCCTCCAGTGTACTTCTTACGTGAGTACATCGGACGTCAAATGGCGTGGGTGTGGTTGCTATGGCTCATGTCCCAAGCGTGGATCGTTGGGCATACTTGGCTGCCACGATGTGAGCGTGTCGCTGCTACCGACAAATTGTTCGCCAAACCTTTGTACAACGGGGTTTTGATAGATCAATCGCTCCTATTGAATAGAACCAAAGATGATGATGCCGATATTGGGTTTGAGAATTCTAAAGACGCCGACAATGTATCCATAGCCAGTTCGGAAAAAGACATGAGTGACATGACTGATATTAAACCAAGCGACAGCATCAGCAGAATTTACATATGTGCGACCATGTGGCACGAAACAAAAGATGAAATGGTTGATTTCTTAAAGTCTATTTTGAGACTCGACGAGGACCAGAGTGCTCGCAGAGTTGCTCAGAAGTACTTAGGAATCATTGATCCTGATTACTATGAGTTGGAGTGTAACATTTTCTTTGACGATGGTTTTGAAATATCTGATCACAGCGCGGACGACTCTCAAGTGAATCGTTTTGTGAAGTGTCTAGTGGACACTATAGACGAAGCGGCCTCAGAAGTCCACCTCACGAACGTTAAGCTTCGTCCTCCTGTGAAAATACCGACTCCATATGGCGGCAGATTGGTCTGGACTATGCCAGGAAAAAATAAAATTATTTGTCATTTGAAAGACAAATCGAAAGTTCGACACAAAAAACGTTGGTCGCAGAGGGATGGTATTTTTGTCTTAGGGCTTATTGGGTAG
