# Supplementary material for: Transcriptomic analysis of developmental features of Bombyx mori wing disc during metamorphosis
Source: BMC Genomics. 2014 Sep 27;15(1):820. doi: 10.1186/1471-2164-15-820 (PMC4196006; doi:10.1186/1471-2164-15-820)
Supplement: Supplementary file 9 — Additional file 9: Assembled nucleotide sequences of transcripts in Table 8. (DOC 44 KB) [file 12864_2014_6525_MOESM9_ESM.doc]

**>Bm_nscaf2964_172**

ATGGCCTTCGAGGACCGCTGCAGCCCTAGCCAGGCTAACAGTCCGGGACCGGTGACGGGGCGAGTCCCGGCGCCGCACGCCGAGACCCTCGCATACAGCCCGCAGAGCCAGTACACTTGCACCACAATAGAATCGAAGTACGAACGAGGCTCTCCGAACATGACAATTGTGAAGGTGCAGCCTGACTCCCCGCCTCCCAGCCCGGGACGCGGTCAGAACGAGATGGAATACCAGGACTACTATCGCCCTGAAACGCCCGACGTAAAGCCACACTTCAGCCGGGAGGAGCAGAGGTTTGAACTGGACAGATCGAGGGGGCAGCGGCTGCAACCCACCACGCCGGTCGCCTTCTCCATAAACAACATCCTGCACCCTGAGTTCGGCTTGAACGCCATCAGGAAAACGAACAAAATCGAAGGTCCCAAGCCCATTGGACCGAACCACAGTATCCTCTATAAGCCTTACGATTTATCCAAACCGGACTTATCGAAATACGGCTTTGATTATTTGAAGAGTAAGGAAACGAGTGATTGCAACGCTTTGCCGCCTTTAGGAGGGTTGAGGGAGACGGTATCGCAGATCGGCGAACGCTTGTCCAGAGACAGGGAGCCTCCGAAGAGTCTGGAGCAGCAGAAGAGGCCCGACTCCGCGAGTTCCATCGTCTCGTCGACGTCAAGCGGCGCGGTTTCCACCTGCGGAAGCTCGGACGCGAGCTCCATCCAGTCCCAGAGCAACCCTGGCCAGCTGTGGCCAGCCTGGGTATACTGCACCAGATACAGCGACCGACCTAGTTCCGGTGAGTGCAAATTTTAA

**>Bm_nscaf3048_40**

ATGCTTGCTCATGGGTGTCGGGCGGCAAGCAAGGGATGCAAGTTCAGGCTCGGTGGTGGACGTGCAGTTGAGTCGGGTTGTGGCAGTTGCTCTCAGTGTCGCTCAAGTCGCGGCGCGCGGTGGTCGTGCGTCGAGCGCGTGTGGAGTGATGTCTGCGAGATGAGGCTCTCGCTGGTGCTTCTGTGGTTGGGTGCGGCGGCGGCATGCGGCCCGGGCCGCGGTTTCAACCGTCGCCACGGACCGCGCCGCATCACCCCCCTTGTCTTTGGCCAGCACGATCCGAATGTTAGCGAAAATAAGAACACCGCCAGTGGTCCTCCCGAGGGCCGCATCACCAGAGATGACGAGAGATTTAAAGACTTAGTGCCTAATTATAATCCGGATATAGACTTTAGGGACGACGAAGGCACTGGCGCTGATCGTCTAATGACTCAGGCTAAGATATTTCCCTTGTAA

**>Bm_nscaf2993_221**

ATGTCATCTGCCAGGACAGGTGGGTCGAGGAGAATTATGCGTGGGGCGTGCGCATGCGCGGTGGTGTGCGCGTTGGTGGCGCTGTGCGCAGCCGCGGGCCTCGACGAAGCGACGCGTGTTGCTGCAGAGAAGCAGTTATTGGCGTTGTTGGGTCTGCCGAAGAGACCGTCGCGTCGATCCGCTCCAGTACCGCCTATACCACGCGCCATGCGAATGCTTTACGAGGCAAGCGGAGCCATACCGGCCGCTGCGGCAAACACGGCCCGTTCATATCAGCACGTACCGACGGAGCTCGATGCGAGGTTCCCAGGCGAACATCGTTTTCGCCTATTCTTCAACCTAAGTGGAGTACCCTCTGATGAAGTAGCTCGTGGTGCTGATCTCAAATTTCATCGCGCGACTGAAGAGACGGGTCCTCAGCGCCTATTACTATATGACGTTGTACGTCCTGGTCGTCGAGGGAAAACGACTCCAATTCTAAGACTTCTCGATTCCGTGACATTATTGCCAGGCGAGGGCACAGTGACAGCAGACGCCATTGACGCAGTGCGACGGTGGCTCCTTGAAACTGATCAAAACCATGGACTATTAGTGCGTGTTATTGAAGAAGGCCAACACAACGTTGATGCAAAACGGCCACACGTAAGAGTTCGAAGACGAGCGACCGAAGACGAAGAAGAATGGCGCTCTCAGCAACCCTTGCTGTTGCTGTACACTGAAGATGCGCGAGCCAGAGAAGCACGCGAGAATGGGGAGTCGCGTCTAACTCGAAATAAAAGAGCAACACAACGGCGTGGTCATCGACCTCACCACCGTCGTAAAGAAGCTCGTGAAATCTGCCAGCGGCGGCCACTGTTTGTCGACTTCGCGGAAGTCGGCTGGAGCGACTGGATTGTCGCGCCTCCTGGTTACGAAGCTTATTTCTGTCAGGGTGACTGCCCGTTCCCGCTCGCAGATCATCTAAATGGCACTAATCACGCAATAGTGCAAACTTTAGTGAATTCAGTGGACCCAGCCTTAGTGCCTAAAGCGTGTTGTATACCAACACAACTATCGCCTATTTCTATGTTATATATGGACGAACATAATCAAGTGGTGCTTAAAAACTATCAGGATATGATGGTGATGGGTTGCGGTTGCCGATGA

**>Bm_nscaf2210_128**

ATGCCAGAATACGGACCACATATGGCCGTGCCGGGTCCCCCGCAAATGTGTCCTGGTACGTACGCGGGACCGCCTCCGGGGGCCCACTATCCCCCGTACCCGTCGCCGGACTTCACCCGTGTCGACCCGGATGTGCAGAAAGGCCCGTATTTTAACGGCGCCGCAGCGCCCCCTCCGCGACAAAAAGGACGCCCACGAAAAAAAAAGCCAAAGGATCAAGACATCATGACCGCAAACCTGGGTTTATTTATTTGCCGTGGATTTGTTTTAAGTTGTGAAATGACAAAGGCACAATATTTAAGGCGTGAATGGTTGTGGGAACTTCTTGTTGTCTCCCTCGCACACCTGCATGGTGGTTGTCAATGA

**>Bm_nscaf2210_130**

ATGTCTCTAACGGGCGAGACGTTGACGAGACGAGTGACGAACAAGCGTAGAAAAACAACATCAGTATTTGTAAATAGAGATCGTTGGTTCCAAATTCGGGCCACCGCGACGCTGTTATCAGCAGGAGACAGTCGCGGACGTGCTACCATCAATCGTGCTGTTGACTGTCCGACGTTCATTTTTCATTCAGACGAACGATTAAATCTGCATTCTGGAACGCAACCCACATCGGCGTACGAGGAACGCTTTCACGCTCTTCGCCAAATGAAGCGCGTCGTGGAGGTATTCAAGGAGGATGGAGCTTCCGCGTCCCGATGTCGCCGCGGGGCCAAACCACAGAGCCGCGAGCCGGCGACCGCCGCACAATTTAATGTATGGTTCCAGAACGCACGGGCGAAATGGCGGCGCATGGTCACGAAACAGGAGAACAAGATGTCGGACAAGTGCTCACCGGACGGCTCGCTCGAGATGGACATGTACCACGGGCCCATGGGCTCCATCCAGTCGTTGCCCCCGCACAGCCCGCCGTACAGTGTGATGGGAGGCCCGCCCAGTCCCAACTCCATGGAGTGTCCATAG

**>Bm_nscaf2210_132**

ATGGAGTCTCTGGCTTATGACTCTTCAGTGGCCTCTCCAGGCAGCGTTTCTAGCCACACGCAAAGGACCAAGCGAATGCGAACCAGCTTCAAGCATCACCAGCTACGGACCATGAAATCTTACTTCGCAATCAACCAGAACCCTGATGCGAAAGACTTAAAGCAATTGGCCCAGAAGACTGGACTCTCGAAAAGGGTCTTACAGGTCTGGTTCCAAAATGCGCGCGCAAAATGGCGACGTAACATGATGAGGCAAGAAACGAACGGGGTTAATGGTCATACTTCGATTGTCCCTGGTAACGGTGGAACACCAAGTATAGTTCCCACCGCGCTGATCCTCTCGGAGCCTCTCCAGCCCCTTGAAGATTTGAGGGTACATACTCCCCATCCGATGGCGTTCAATGAACTTTACTGA

**>Bm_nscaf2860_94**

ATGTCGGTAAATTATACCGCAAAGCTAATTATATTGGCAGCAGCACTTCCACAATGGTTCTGTCACTTTGACGACGACAACTACGTAAATGTTCCTCGTTTGGTGAGCGTGCTACAAACCTACAAGCACCAAGAAGATTGGTATCTAGGTAGAACGTCTGTTTACGAGCCGGTCAAAATATACAAGAAACCAACGAATAAGTTAATGTTTTCGTTCTGGTTCGCCACCGGGGGCGCTGGATTCTGCATAAGCAGAAGTTTAGCTCTTAAAATGCTACCTGTTGCAAGCGGTGGAAGATTTATAAGTATTTGTGAAGGCATACGGTTACCGGATGACGTGTCTATAGGATTTATTATAGAGCATCTAATGAAGAAGAATTTGACTCTGGTCCCGGAGTTTCATTCTCACTTGGAGCAGATGAAGCTTCTTCCTCCGGAGACATTTAGGGATCAGATCTCCTTTAGCTATGCCAAAGCGAAGGACGAATGGAATGTCGTGAACGTTCCTGGATTTGACACTAGATACGATCCTACGAGATGGAATCTATCGGCTCACTTTTTATCAATACGAGGTAGAGTAAATTAA

**>Bm_nscaf2860_96**

ATGCTTAAGGCCGCAGCTGTGCTGTTAGCCTTAGGATACTGCAGCTTATTAGTATACCAAGGAGGAGTCAACTTCAACTTCCAAGAAAGTCGGGCAGGTGTTGTGCAAGTGGCCGATCTATCAATTGAATCCATTACTAAAACAAGTGTTGACGATATTGAACTAAACAAAAACATTACCTTAAACGATATTTTTATTAGTGTTAAAACAACTAAACATTATCAGTACACAAGATTACCGATCATCTTGAAGACTTGGTTCCAGTTAGCAAAAGAACAGGTACTACTTCCAATTTCATTTTCGCCCCTTAAATGCAACCGTCATTCGTACAATTAA

**>Bm_nscaf2986_021**

ATGACGAAGCAAAGTGTGGCCGATGTCGGAGGTCCGTGGATAGAGGAGGAGCAGCGTTGGGGAGGTCCGTCCGAAGCCCACTTAAGGGTTTCGTTCCGAGTCACCTGCGCTCCCCATTACTACGGTGCCGGCTGCGCTGTGCTGTGCCGGCCCCGGGACGACAGCTTCGGCCACTACACCTGCTCGTCTGCCGGGGAAATCGTGTGCCAGGATGGATGGACCGGAGACTATTGTAGCAAACCAAAATGCCTGGCCGGCTGCGACTCCGAGCACGGTTACTGCACCAAGCCCGACGAATGCATATGTCACTCGGGGTGGGTGGGCAAACGCTGCGATAAGTGCGAGCCGCATCCGGGATGCGTCCACGGAACCTGCTCGAGGCCCTGGGATTGCATCTGCAAAGAAGGTTGGGGTGGACTTTTCTGCAACCAAGATCTGAACTACTGCACCAATCACCGACCGTGCAGAAATGGAGGCACTTGTCTTAACACCGGTCAAGGCAGTTATAATTGCGTGTGTCCACCTGAGTATACGGGATCGGACTGCGAGAAGTCTCTACACTCATGCGCCGTACGCCCTTGTCTCAATGGAGGCCTTTGCGTACCAGATGAAGGTGGCGAACTTTCCTGTACTTGTCCCCAAGGATACGAAGGAGCGCGCTGTGAGACGCGGCGACTTACCTGCTTTGATCGACCTTGTCACAACGGAGGAACCTGTGAACCGAAATCCTCCGGCTACGTGTGTGTATGTCCGGTAGGGTTCGCGGGCACGGACTGCGCCCTCGAAGCCGACCCATGCGCCGCCAACCCCTGCCGCAACGGAGCCACCTGCTCCCGAGCAGGCAACGGCTTCAAATGCACGTGCAGAACAGGCTTTAGAGGCAACCGATGCGAGATTGACATAGACGACTGCGCCGGCATCTTGTGCGAACACGGCGGGACCTGCGTCGACCTTGTTAACGGACAGAAATGTCAGTGTGCTCCCGGATTCCTCGGCCCCCGCTGTGAAACTCGAGTAGACATGTGCTTGACAAAACCGTGCGCAAACGGCGGCGAGTGTCTAGTTCTAGACAACGACTACGGGTGCCGGTGCCGGCCTGGCTTCACCGGCAAGGATTGCAGCATCGACATCGACGAATGCGCCTCTTCGCCGTGCCGGAACGGAGGAACGTGTCGCGACCGAGTGGACGGGTACAGATGCGTGTGTCCGCACGGATGGGGAGGGCGCTCTTGTACCGTGTCGCTGTCGGAGTTGGCAGCGAAACAGGCAGGCGGACACTTGCCGCGCGTAGGTGATTCCGACGAAGAGGAGCGGCTGTCGGCGCAGCAGGTGGCGTGGATAGCGGCGCTGGGCGCTCTGGTGCCGGCGGGTGCAGGTGCGGCTGCGCTGGCCGTGGTTTGCGTCCGGCGCCGGAGAGCGCGGGCGGCCGCCGCTGCCGACGCGGAGGCGCGCGCCCAGAACGCGGCCAACGCGGGCGGCGGTCACGTGATCCGCAACACGTGGGGCAAGTGCGACGCGCCGCCCCCTGAGTGTCAGAACGCGCACAACGCTGCCGCCGAGGAGTGCAAGCGCAAGACGCTCAACACCGAGAGCGCGCGCCTGCTCGCCGCGCTTGATCCCCGACTCTCCAGGCTCTCCGCAGACTCGGCGTATTGCGCGAATAGCGATACGTCGTTAGTGAAGCGGGCGCTGGAGGGCGGGGGGGTGTACGTGCTGGACGACCACTGCTTGCCGCCGACGTTCGCCACGCAAGTGTAG

**>Bm_nscaf1705_01**

ATGAGCACCTACAACGTCAAGTTTTGCGCGGCGGGGGCGGGCGTGTTCGAGCTTCAAATACTGGAATTTAGCAACTATCGGCTGGAGGTGGGGGCGGGCGGATGCTGCGGGGGCGGCGGTCCGAGGGGCGCGGGAGCGGCGGCCTGCTCGCATCCCTGCCGCACCAGGTTTACGCTCTGCCTCAAGGAGTACCAGTCGGCGGCCGCGCCCGGCGGGAGCTGCTCCTTCGGGCGCGCCGCCTCCCCCGTCCTCGGCACTGACTCCTTCACTCTGGCTGAGCCTCTCTACACCCTCGCACTGCCCTTCAGCTTCCGATGGACGCGTTCGTTCACGTTGATCCTGCAGGCGTATGACGACTACGAATACTCCGAGCCCGGTGAGTGGCAGCCGGTGTCGGCTGACGGGGGCGGACCAGAGTGTTGGCTGTCGGGCTGTTGTGATGGATCCGGATGGTGTGTTGCAGAAGCGGGGCTCATCGAGGAGGCGTGGTGGTCCGGGATCGTGGAGCCGAGCGCGGAGTGGCACGCGCTGCGCCACGCGGGCGCCGCGGCTGCCGTCGCGTACCGCGTGCGGGTGCTCTGTCAGCCCAACTACTACAATACGACCTGCACCACGTTTTGCCGGCCCAGGGACGACAAGTTCGGCCACTACTCGTGCACGCCCGACGGCGACAAGCACTGCCTGCCGGGCTGGCAGGGGGACAACTGCGAGAAACCTGTTTGTAAGGAAGGCTGTCACCCGACTCACGGACGTTGCGATCGGCCCGGGGACTGCGATTGTCGACCGGGATGGAGAGGTGAACTGTGTTCTCAGTGTCAGCCGTATCCGGGGTGTAAACACGGCTACTGCAACGGTTCCTCCTGGGACTGCACCTGCGACACGAACTGGGGCGGGATACTTTGTGACCAAGATTTAAACTACTGCGGCACCCACGAGCCATGTCAGCACGGAGGCACCTGTGAGAACACCGCGCCGGATCAGTACTTCTGTCGGTGCGCCGAGGGCTTCTCCGGGGTGGACTGCGAGCGCGTCGACAACCCGTGCGCGCCGCAGCCCTGCGCTCATGGCACGTGCTCCCTCGCCGGCACGACGCGAGGCTTCACGTGCACGTGCGACCGCGGCTGGGGCGGGCTGCTCTGCGACACCGACCTCGACGACTGCGCCAGCGGGCCCTGCCTGCACGGCGCGTCCTGCAGGGACCACCTCGACGGCTTCACCTGCGAATGCGCAGACGGCTGGACCGGGCCAGCCTGCGCCGAAGACGTCGACGAGTGCTCGGGTCGAAGCATGACGGAGGGCGCGCTGGGTCCGTGCGTGAACGCGGCGGCCTGCAACAACACGGCGGGCGGGTACTCGTGCGCCTGTCTGGCGGGCTGGACCGGCCGGGACTGCGAGACCAACGTGGACGACTGCACGGGACAGTGCCTGCACGGCGCCACCTGCATCGACCTGGTGGACGACTTCCACTGCGCCTGCGCGGCGGGCTACGCGGGCCGCACGTGTTCGCTGGACGTGGACGACTGCGCCCCCCGCCCTTGCACCAACGGCGGCGAGTGCGTCGACCTGCTCAACGCTTACCGCTGCATCTGCCCCGTCGGCTTCTCCGGCACCAACTGCGAGGACGACCGCGACCACTGCGCCGGCTCTCCGTGCGGCAACGGCGCCGCCTGCTACACCGCGCAGAGCGACTACTACTGCCACTGCGCGCCCGGCTGGACCGGCAAGAACTGCACGCAGCGAGCTGCCAGGGACCGTAAAACCTGCTCAGTACTTGTAGAAGAGAGCGAGCGCTGTGCGGAAGGAATGTGCGCGAACGGAGGCACTTGTGTGCGCGAGGAGGGGTCCTGGCGCTGTCTGTGCGCTGCGGGTTGGGGGGGCGGCGCCTGTGACTCCCTGCTGCCGCTCGCGCCCGCAGCTCCTCCGCCGTGCCCGTGCCAGGCGGGGGGGACGTGTCTGCCGTCCGCACTGCCTCCCGGCTGGGCTTGCGGGTGCAGAGAAGGTCGTACCGGAGCCTTGTGCGAACTGTCCCTGGATCTCTGTGGCTCTTCGCCTTGTCGTAACGGCGGTCGGTGCGTCGGTGGCTCGGGGTGGTGGATGTGCGAATGTGTGGCGGGCTGGACCGGAGAGACTTGTGCAGTGCGAGTGTCCGCCCCGCCCCTCCTGTGCTCCCCGACCTGTCCCGCCCCCGCTGCCTGCGTCGCCGACCCCGCACGTCCCTCTTCCACCCGGTGTCTCTGTCCGGCGCCGCCGCAGCGTGCTGCTAGACGCTGCCTCGAGACTGTTGCCGGCTTGGAAGCAGAGGGCGATGACGTAGACGTTCTACAAAACGACAACCATGGAGTGTACAACCGACCAGACCCGAACGTGGGCGACGGACCGGGAGCGTGCGGCGTCGCTAACGGCACCTGGTGGTGGGGATGTAACGCATGTTGGTGTAGTGGGGGGGTGCCAACCTGCACTCGACTGTGGTGCGGCCTGCCGGACTGCCTAGCTCCTAACGCACAACCCTGCCGAACCGATGAATGGATCGGAGACGAAGTGGGTTCAGACGGAGCCTCCGGTGCAGCGGCGCTTGGAACTGCAGTGCGGGCTCTCGGAGAGCTTGTGTCGCGTCGGCGGCTGGCCCGTCACGCCCTGCTCGGGGCAGTGCTGCGACTCCGCATGCCGCCAATAGCGCCGCCGGCCTCAGAGCCATCGACAGCGCCGGCGACCACTATCGCGGTCGGTCTACCGGTGGTGCTGCTAGTAGTGGCCGCGGCCATTGGCGCCTTCCTGTTCGTGCGGAGGAGACGTGTGGCGGCAGCGGAACGGTCACGGCGCTGCGACGAAGAGAAGTCTAACAACCTTCAAAACGAGGAGAACTTGCGGCGGTACGCAAACCCCCTGCGCGAGGAGCGTGGCGGGACCCGCGGCGAGGACCTGCCTCGGGCCCATTCGCTGTACAAGGCGCAGAACGCGGACGCGCGGAACGACACGCCGCCGCGGGACAAGGAGCTCACCCTCCGCGCCCTGCCGGCCCCCGAGCCGCCGCCCCCCGCGCGCCACCCGCCGCCCGAGCGACTCACTGTACTCGTGTGA

**>Bm_nscaf2888_347**

ATGGCAGCAGGTGTCGAATGCTCGCACGAAGACCATTGTGCATCTCAGCCATGCCGTAACGGAGGACGTTGTGTAGCTGACAACACGACCGCGGCCGGCTACTCCTGCGTCTGTCCACCTGGCTTTACTGGCTCTCGATGCACGGAAGACGTTGTCGAATGCTCAAGCGGCTCTGGTCCATGTCACCACGGTAGATGCTTCAACACCCACGGCTCATATACGTGCGTTTGTGAACCTGGGTATACGGGAAGAGACTGTGATGCGGAGTATGTTCCTTGTGAGCCGTCTCCCTGTCTTCATGATGGAAGGTGCACGCCGTTGGATCAACTCCGATACGAATGTGATTGTCCGCCAGTCGCAGTTCAGAAAGTCGACCATGCCCTAAGGCGACCACGACTATCCAGTTCGAAAGTCGCAGCCCGCGGGTCTTGTGATATTTATGGCTGA

**>Bm_nscaf2888_348**

ATGCGTCCTGTCTTTCCAGGTCTCCTGTGTCACTTGGACGACGCTTGCACTTCGAATCCTTGTCACGCGGACGCCATATGTGACACAAGCCCCATCAACGGCTCCTACACGTGCTCCTGCGCCTCCGGATACAAAGGTCTGGACTGCTCCGAGGACATCGACGAGTGCGAACAAGGTTCTCCCTGCGAGCACGACGGGATCTGCGTCAACACCCCGGGATCCTTCGCCTGTAACTGCTCGGTCGGTTTCACCGGACCCCGCTGCGAGACAAACGTCAATGAGTGCGAGAGTCACCCATGTCGGAACGACGGCTCCTGCCTGGACGACCCTGGCACGTTCAGATGCGTCTGCATGCCTGGAGAACGGAAAGCGCATATTAGTTTCAGCGGAGACAATCGAAAAACATTATTGGGCGTTGTCGTGAACCGTAACTAG

**>Bm_nscaf2888_349**

ATGTCGTGTGAGACGAACATCAATGACTGTTTGTCGGCGCCGTGTCACCGGGGCGAGTGCATCGACGGGGACAACAGCTTCACCTGCAACTGTCATCCGGGGTACACGGGCAGGGTCTGTCAGACCCAAATCAACGAGTGCGAGTCGAATCCCTGCCAGTTCGGAGGGCACTGCGAAGATTTGATCGGTGGATACCAGTGTCGCTGTAAGCCCGGTACTTCAGGGAGGAACTGTGAAATCAATGTGAACGAGTGCTACTCCAACCCTTGCAGGAACGGGGCTACGTGTATTGATGGCATCAACAGATATACCTGCGAATGTATTCCCGGTTTCACTGGACAACATTGCGAGACGAACATCAATGAATGTTTGTCAAATCCTTGCGCTAATGGCGGCAAATGTATCGATCGAATTAACGGATTCAGATGCGAATGTCCAAGAGGATACTACGATGCTAGATGCTTGTCCGATGTTAACGAGTGTGCGTCGAATCCGTGCACCAATGGCGGCTCCTGCGAGGATGGCGTCAACCAGTTCATATGCCACTGCCTTCCTGGTTACGGAGGACAACGTTGTGAACGCGATATAGATGAGTGCAGCTCGAACCCTTGTCAACACGGCGGCACTTGTCATGATCGACTGAACGCTTACAAATGCGACTGTATTCTTGGATTTACCGGCGTGAACTGCGAGACGAACATCGACGACTGCGCCGGCAACCCTTGTCTGCACGGCGGCTCGTGCATCGACCTGGTGAACGGGTACCGCTGCGTCTGCGCCCCCCCACACTCCGGACGAAACTGCGAAAACACCCTCGACCCTTGCATGCCGAACCAATGTCGTCACGGCGGGCGGTGCGTGGCGGAGGCGTCGTACGCGGAGTTCACGTGCCAGTGCCCGGTCGGGTGGACGGGCGCGCTGTGCGAGCGCGACGTGGACGAGTGCGCGGTGACGGCGCCCTGCCACAACGAGGCGACCTGCATCAACACGGAGGGCTCGTACGCGTGCCTCTGCGCCAGGGGATACGAGGGCAAGGACTGCGCCATCAACACGGACGACTGCGCATCGTTCCCGTGTCAGAACGGAGCGACATGTCTGGACAGCATCGGCGACTACAACTGTGTTTGCGCCAGCGGCTTCGCGGGCAAGCACTGCGAGGTGGACATCGACGAGTGCCAGTCGAGACCCTGCATGAACGGCGCCACTTGTAACCAGTATGTTGCGTCATACACGTGCACGTGCCCGCTTGGCTTCTCCGGCATCAACTGTCAGACCAACGACGAGGACTGCACCGAATCGAGCTGCATGAACGGAGGGACCTGTATCGACGGCATCAACTCTTACAACTGCTCCTGTCCGCCGGGGTACACCGGCTCCAATTGTCAGTTCCGCATCAACATGTGCGACAGTTCACCGTGCGACAACGGCGCGACATGTCACGATCACATCACCTACTACACATGTCACTGCCCGTATGGCTACACCGGAAAGCACTGCGAAGACTTTGTTGACTGGTGTGAGAACAATCCGTGCGAGAACGGAGCGACATGTTCGCAGAAAGGTCCCCAGTACACGTGCACCTGCGCCCCGGGATGGTCCGGGAAACTGTGCGACGTCGAAATGGTGTCTTGTAAAGATGCTTCCATCAGAAAAGGCGTAAAACTAAAGCAGCTCTGCAACAACGGCACCTGCGAAGACATCGGCAACTCTCATCGTTGTCACTGTCAGGACGGCTACACCGGCTCCTACTGCCAGAAGGATATTAACGAATGCGAATCCGCTCCTTGTCAGAACGGAGCTCTCTGCAAGGATCTCGTCGGAACTTACCAGTGTCAATGCGCCAAAGGTTTCCAAGGACAGAACTGCGAGCTCAACGTCAACGACTGCCTGCCGAACCCGTGCCAAAACGGAGGAACATGCCACGATCTCATCAACAACTTCTCATGCTCCTGCCCTTTCGGCACTTTGGGGAAGATATGCGAAATAAACGTTAACGATTGCAAACAAGATGCGTGTCATAACAACGGCACTTGCATCGACAGAGTCGGCGGCTTCGAGTGCAAGTGTCCGCCCGGCTTCGTCGGACCGAGGTGTGAAGGCGACATCAACGAGTGCCTCTCGAACCCTTGCTCGCTGCCCGGTACACAAGACTGCGTGCAACTCATCAATGACTATCACTGCAACTGCAAACCCGGGTACATGGGAAGGCACTGCGATGCCAAAGTCAACTTCTGCGCTAACTCGCCCTGTCAAAATGGCGGCATATGCACCGCTATACAGGGTGGCCACGAATGCTTGTGTAACGAAGGCTTTTACGGGAATAACTGCGAATATTCAGGCTACGCTTGCGATTCGAATCCGTGTCAAAACGGTGGATACTGCCACACTTCCCAAATAGGAGGATACGCGTGTGAATGTCCGTTGGGATTGTCCGGTGTCAGCTGCGAAATAGATTCAATGAACGAATGCTTGAGTAACCCGTGCAAGCATCCGGAGGCACGGTGCATAGACAAGCCCGGAGACTACATATGCTATTGTCCTAGACAATGGACAGGGAAGAACTGTGATATTCACGATCACAACGCCAAAGGCGGATACGGTAGTCCGATAACCGGCATTTTTAGCAACAAAAATCCGGGACTGACTCTAGAGGAACTTGATTTAGCCTTCCAAAGAGAGCAGTGCGTTAAAAAGGGCTGCAAAGAGAAACAGGGTGACCACCATTGCGACGAGGAATGCAACACGTACGCGTGTGAATTCGACGGCAACGATTGCTCGTTGGGCATCAATCCGTGGGCAAACTGTACGGCTCCGATCAACTGTTGGGAGGTGTTCATGAATGGTGAATGTAACGAGGTCTGCAACACCCAAGCTTGTCTGTTCGATGGAAGGGACTGTCAAAAGTCCCTACAGAGATGCAACCCTATATATGACGCGTACTGCCAGAAACATTACGCCAACGGTCATTGCGACTACGGCTGTAACAACGCCGAATGTAATTGGGACGGTCTTGACTGCGAAAACGAACCGCCTGATTTAGCCGAAGGAGTGATGTCCGTCATCCTGCTGATGGACATGAAGACCTTTAAGGAGAACTCGGTGGCCTTCCTCAGAGATCTCGGTCATCAGTTACGGACCACCGTTCTGATAAAGAAGGATCATTTAGGTAATGACATGGTGTTGCCTTGGAAGGGCTCCACTGACGTTGGACTGGAAGAGACCGAATTCGGAAAGAAACATCATATTGTTTACACGGAAAGAGGACAGTCCGGTGTGCAGGTGTACTTAGAACTTGACAACAGAAAATGCACTACAATGTTAAGTTCAGAGTGCTTCTTCTCAGCCAGAGAAGCCGCCGATTTTCTTGCAGCCACGGCCTCCAAACATTCGCTGTCACCGGACTTTCCGATTTACCAAGTCAAGGGCGTAAACCCTCCTATTACTGACGAAGTTCCTACAAACTCTAAATACGTCTTCATTGGAGTCATTTTGGTTCTGCTAGCCGGTCTCCTGATAGGAGTTTTAGTAACCGCTCAGAGGAAACGCGCCGCCGGCATAACATGGTTCCCAGAAGGATTTATCCGTTCGAACTCCTCGACACGGCGTCGGTCACGCCGCCGTGGACCAGACGGCCAGGAGATGCGGAATCTCAATAAAGGATCCATAGGCTGTATCGACGTCGATATCAATGGAGGCCACATGGGTCCACCGCACTCTTGGTCTGACGAAGACGACGATGGCTCGGCCCCTCCCAGAGCCAAGAGAGCCAGAGGACCAGGTGACGCGAATGGGGCTCCAGGAGGCTATGCCTCAGATCACACGGCCATCACTGATTATGAGGAAGCCGGCCATGAACCAAGAGTGTGGACTCAGCAACATTTGGATGCGGCAGATATCAGAGTACCGCCCAGTATGATGACCCCGCCTGCAATCCACGACAGTCACGTGGATGTTGACGTCCGTGGACCGCTGGGTATGACGCCGCTGATGGTAGCCGCAGTGAGGGGAGGAGGTCTGGACACCGGCTCCGACGTAGAAGATGAACAGACAGCCCACATTATATCCGAACTAGTCGCTCAGGGCGCTCAGCTCAACGCGGCCATGGATAAGACCGGGGAAACGAGTCTGCATTTAGCCGCGAGGTACGCCCGTGCGGACGCAGCCAAGCGCCTGCTGGACGCGGGCGCGGACGCCAACTCGCAGGACAACACCGGGCGGACCCCGCTGCACGCCGCCGTCGCCGCCGACGCGATGGGCGTCTTCCAGATCCTACTGCGGAATAGGGCCACCAACTTGAACGCTAGAATGCACGACGGAACCACGCCTCTGATTCTGGCAGCTCGGCTTGCCATCGAGGGCATGGTGGAAGATCTAATCAACGCCGACGCGGATATAAATGCAGCAGACAACAGCGGCAAGACAGCCCTGCACTGGGCGGCGGCTGTCAATAACGTTGACGCTGTCAATGTACTACTCGCACACGGCGCCAATAGGGATGCGCAAGATGACAAGGACGAGACTCCACTATTCCTGGGAGCGAGGGAGGGCTCGTACGGCGCCTGCAGGGCTCTCCTCGACGCGATGGCGAACAGGGAGATCACCGACCACATGGACCGGCTGCCGAGGGACGTGGCACAGGAGCGGATGCATGACGACATCGTGAGGCTCCTCGACGAACACTGTCCGAGGCCGCCTCCGCAGCATCCGCATCTCATGACCTCGCCGAACCCGCATCACCAGCTGATAAGTCAGCCGACAGTGATCGCGTCTGCGGCTAAAGGCAAGCCCAAGAAGCCCAGAGCGAAGGCCGGCCCAGACAGTCCTCAGGATCAAGTTTACGACAACAATAATCTACAACAGACACAGATAAGACGGAAACCAAGCGTAAAAAAGAGCAATAAAAAGATAGCGCAGGAAGTGCCGCAGAGCGTTGAGAGTCTCGGCTCCAGCCTCAGCCCAGTTGAATCGCCGCTCCAAAATTTACAGGACTTGCCGTCGCCATACGACGCAACGTCACTGTACTCGAACACGATGGCGCAGTTCGTCGGCATGGAGCAGCTACTGCACCACAAGCAGCCGCCAAGCTATGAGGATTGCGTCAAGACGGGTCAGACGCTGCAACAGTCGTACGGCGGCGGCGCGATGGGCGCGTCCTTGTCGCCGCCCTACTCCAACCACTCGCCCACACACAGCAACCAGACCACATCGCCGCACGCTTACATGGGCTCGCCGTCGCCAGGAAAGTCGCGACCGTCGCTGCCGACGTCACCGGCGCACATGGCGGCGTTACGTCACTCGCACCAGCACCAGCTGGACGCGTACTCAGCACTCGCACATCTGAGCGCGAGCAGTCAGCACAACGCGCAGCTGCAGGCGGTGATGACGCACGCGGCCGCACTGGGCCAGGTCCAAGGGGCGCAGCATCCGGCTCTCACCAATCTTATGTCGGGCTTGTACAGTTGGCACACCGGTATGGGTGACACGTTTCCGACGCCGTCTCCCGAGTCCCCGGACCACTGGTCGACCCCCTCCCCGCAGACACCCCTCACGCAGTCCCCGCACTCCGACTGGTCCGACCGCGCCGCCCTCTCGCCCAACGACATCCAACAAACGAACAAAGGCGCCGAAGGCATATACATTTAG

**>Bm_nscaf2847_167**

ATGATCGAATTGATAATCTCTCGTGTAGGTTGCCGTGAGACCGCCTTCATCTACGCTATAACGAGCGCCGGAGTAACCCACTCCGTGGCCCGCGCCTGCGCCGAGGGCTCCATAGAATCGTGCACGTGCGACTATTCGCACATCGACCGCGTGCCGCACAGAACCCGGTCGGCGGCCGCGGCCAACGTGCGCGTCTGGAAGTGGGGCGGCTGCAGCGACAACATCGGCTTCGGATTTCGCTTCAGCAGGGAGTTCGTTGATACCGGAGAACGAGGCAAGACTCTAAGGGAGAAAATGAACCTGCACAACAACGAAGCCGGCAGAGCGCACGTGCAAACGGAGATGAAGCAGGAGTGCAAGTGCCACGGCATGTCAGGCTCGTGCACCGTGAAGACTTGCTGGATGAGGCTGCCGAGCTTCAGGTCGGTGGGTGACTCGCTGAAGGACCGGTTCGATGGCGCGTCACGCGTGATGCTGTCGAAGGCGGAGCTCGAGACGCCGGCGCAGCGCAACGAGGCGGCGCCGCACCGGGTGCCGCGCAAAGACCGGTACCGGTTCCAGTTGCGGCCGCACAATCCGGACCACAAGTCGCCCGGGGTCAAAGACCTCGTCTACCTGGAATCGTCGCCGGGATTCTGCGAAAAGAATCCGCGTCTAGGCATCCCCGGCACGCACGGCCGCGCCTGCAACGACACTAGCATCGGCGTGGACGGCTGCGACCTGATGTGCTGCGGCCGCGGCTACAAGACAAACACGATGTTCGTGGTTGAGAGATGCAACTGCACGTTCCATTGGTGTTGCGAGGTCAAGTGCAAACTGTGTCGTACGGAAAAAGTGGTGCACACGTGTTTATAG

**>Bm_nscaf3056_42**

ATGGAGAGGTGTCGTGTGTTGGTACAGTTGAACGTGCAGAGCGGCGGCGTCAATGCGGGCAGCAGCGAGGCGTTCGTGTCGGGGGCGCAGTCCCCGGGCTCTCCGCCGCACGCGCTCCCGGCGCACCGTCTGCGCACCAAGGAGGAGGATCTCTCTGCACACGGCCGAGCCAGCGCTGACGGAGGCGGTTCGTCCGAGTCGGAGGGCGAGTGCGGCGGCGCGGGTCCGCGAGCGCGCGCGCAGTACGTCAGCGCCAACTGCGTCGTGTTCACGCACTACTCCGGGGACGTCGCCGCGGTCGTCGACGAGCACTTCGCCAGGGCGCTCTCGCTCGACAAGACTAAAGATTACAAGCTGTATTGTATAAATGCTGTATTGAGCACGGAAATATTATTCACGTTTGCTCTGAGTGATCGGAAACATAGAATCGAGACGTGTCGCTGCGTATCAACATTCTCTAGGAACGTAACGAGACAAAGGGCGCGCAGTGTACGCGCAGCGGCCGGCCATGTAGCCCCAACAAATCAATATGCAATTAAAATAGCTAACAAGTTTGTAACGATCGGTAACGGAGCTGGCGCGGCGGGGCAGGGCGCGGCGGGGCGGGGCGCGGTGCGCTCCTAA

**>Bm_nscaf1898_116**

ATGTTCGACTTAACAGTTTTCTACGGAAGGAACGAGTTAATAGCGAGGTATATTAAACTAAGGACAGGCAAAACGCGTACGAGAAAACAAGTCTCGTCACACATACAGGTGCTAGCTAGGCGAAAACTACGAGAAATTCAAGCCAAACTTAAAGTGGACGGCGGAGTGATGAAGGAAAAGGCCATGCAGTCAATGAGCACGCTATCTAGCGCTCAGATCGTTGCGGGCCTACCGCACCCAGCGTACCACCATACGCAATTTTGGCAGCCAGGCCTACAAGCCGGCACATCACAGGATGTGAAGCCTTTCCCCGGTGCGGGCTACAAAGGCGTCCCCGGTGTCGGAGGCGTCGGAGTGCCGAGCGGCACCGACGTGGCGCCGCCGCCGCCCTGGGAGGGACGCGCCATCGCCACACACAAACTGAGACTCGTAGAGTTCTCCGCCTTCGTCGAGCATCCCCGGGATCCTGATACGACAAACATCATGTCGCCTTTCCCCAAATTAGCATTCTCTACGCCCTGTGAATCAGATACTGATATACAATATGAGTCGTCTATTATCAAAGGCGGCAATCTGTACATTTGGACAAAAAATTTTTATTGA

**>Bm_nscaf1898_117**

ATGACGATAACGTGTAGCACGAAAGTGTGTTCGTTCGGGAAGCAGGTGGTCGAAAAGGTGGAAACTGAATACGCCCGGTTCGAGGGCGGTCGCTTCGTGTACCGCATCCACAGGTCGCCGATGTGCGAGTACATGGTCAACTTCATACACAAACTGAAACATCTGCCCGAGAAGTACATGATGAACAGCGTACTAGAAAACTTCACTATACTACAGGTGATTGTCCGATACTTGAGTAATGATGCTAAATATTTTCGCGTTCAGTGA

**>Bm_nscaf1898_167**

ATGCTCCAAGAAATCCAGCTAGTTCAAGGTCAGACGAATTACGTGGTCGTTTCTTCCGGATATCCTTCGAATACATTGAATAAATCATCTTTAGAGAAACGAAATGTTGCTATAGCTCCAGCGCCTGAGAAAAATTACGTCACACACGACACTCCACCGAATCTACACTACAGGAAAAAGGTGCATTTCAGAACTAATCCGTACACTGGACCACAGGCTGCGTCAATAGCGAGACGTAATGCACGGGAACGAAATCGCGTAAAACAAGTGAATGATGGATTCAACGCACTTCGCCGTCACCTGCCGGCTTCTGTCGTGGCAGCTCTGTCCGGTGGCGCCAGACGAGGTTCGTCAGGGAAGAAACTTAGCAAAGTCGACACATTACGGATGGTTGTTGAATATATAAGGTACCTTCAGCAGTTATTAGACGAAAGTGATGCCGCATTAGGTATTACGCGTGATCAAGAAAATCGGGAAAATATTCCAAGCAATAACTCAGTTCAGCCGATGACTTCTATTGACATGGATGACGGGTTTTTCTACGGAAGTGGATCACCTTGTTCAGAGAAGGCAGATTCGCCAGCTCCTTCGGAATGTTCTTCGGGTGTGTCTTCGGCGTATTCGGCTGTCGATCGTTACGAGGTTACTACGCAGCAACAAATGGGATCAATGGATGAAGAAGAACTCTTAGACGTCATTTCATGGTGGCAACAAAAATAG‍

**>Bm_nscaf2902_362**

ATGACCATGAAACTACACGCGGCCCTGTTGCTAACTACATTCGTGCTCGCCGCACGCGCAGCCTCCATCCCGGACAAGGTCCCCGAGGCCGAAGATAAACCTTTAAATGTCGTTGAGAACTTATCTAGTGAGCAGGAGCTCATCGACCAGGCTAATACGATTAAGGACATCGATAACAGCCTCCGCGCGAACAAAAAAGAAGTCGTCGACATCCCCGTTAAAGTTATCGTTGAAGAAATCAAACCGTCGTTGAAGAGTGATTTAGAAAACGTTGAAGTGCCGGATGAAAATGAGGAAATCAAGAGGCCTCTAGTCGATTTAAGAAATCCCGGGCCCCCGCAGCATCAAGAGCACGAAACACAGAATCCTGAACACCACGAAGATGCTGAAAAAATCGTTTCTTCCGTCAAAAATGACATTAACACAGCGGAAATCGCTCTTCGTCAAGGTTCCAGGAAGTGTCAGACGGTATTGGAAAATGCTGACACCATCGCGGCCCCTTCTGTCGAGGAAACGCAAAATAAAGCTTCTTTTGAAACAATCGAATCGGGTCTCAAGTCTTTAGAGACAAATTTCAATAGCGGTCTTAATCAGCTATCTGAAGGTATTCAAATTGTGGCTACGTTCAAAGCCGACGGAGAGGCTGCAGCTGAAAGTTCCAGTACCGCCCCTGCTCAAAGCACAACAGCTTCTACAGTAACAAGCACCAATGGCCCTACAAATCCTTTAATTCAAATGGTGACCAACCTCCAGAATTCATTCCTGTCCGGAATGGCTAATCTCACTCAAGCAATCAACAACTGGAACTCGAACCAAGCATGGAGTGTTCCAAATATTTTTGGCGGAGCTAGCACTGCAGCCCCTCAGTCAGATGTTCAAGGCGACGCAACCACCACAACGCAGAGACCTGCGCCGTGGCAAAATCTGCCGTCGCAAATAAGCAACTTTTTTAATCCCCAGGGACAAAACAGTAACCAACAAAACCAGAGCGGTCAACAGTCGCAGGCGCCTTCTGGTCTCTTTTCTGGTGTACAAAATTTTCCATTTAACTTCCTAAACCTCTTACAACCAAATAGGCCTGGTGCTCAGTCTACTGAGAAACCCGCTGAAGCGACTAGTACGAACGGAGCCGCGAGTGCCGCTCCAGACATTGCGAAACCATCGGAGTCAAATCTACCTACTGAAACAAAACCAGAACAACCCGCAGCCGGGCCGTTAAAACAAATTTTCGAGAACAGCCCAGTTCTGCAAGGCATCGCAGGAGCAGTTAAAAAGATCCAAACAACAGTCAATAATCCAGTGAAGCCAAGAGATTCGGAAGTGGTTGAAGAGACTAAATCTGATCAAGAGAAGGGAGGCGTCATTTTGTTGCCAGTACACGGTCACGGCGGTCACGGTGGAAATGGTGGGGATAATAACAACGTCAGTGACGGGTTGAAAGCGGAAGCCGAAGAAATCAAGGTATCCACAGAAGAAAAACAGGAAGAGATAAAAGAGAAAGAAATAATAGTAGAAAACAAGACTGAATGA
